# Bayesian Disease Mapping to Identify High-Risk Population for Oral Cancer: A Retrospective Spatiotemporal Analysis

**DOI:** 10.1155/2023/3243373

**Published:** 2023-11-02

**Authors:** Poornima Ramamurthy, Dileep Sharma, John Adeoye, Siu-Wai Choi, Peter Thomson

**Affiliations:** ^1^College of Medicine and Dentistry, James Cook University, Cairns, Queensland 4878, Australia; ^2^School of Health Sciences, College of Health, Medicine and Wellbeing, The University of Newcastle, Ourimbah, NSW 2258, Australia; ^3^Faculty of Dentistry, Oral and Maxillofacial Surgery, University of Hong Kong, Hong Kong

## Abstract

**Objectives:**

Bayesian mapping is an effective spatiotemporal approach to identify high-risk geographic areas for diseases and has not been used to identify oral cancer hotspots in Australia previously. This retrospective disease mapping study was undertaken to identify the oral cancer trends and patterns within the Queensland state in Australia.

**Methods:**

This study included data obtained from Queensland state Cancer Registry from 1982 to 2018. Domains mapped included the oral cancer incidence and mortality in Queensland (QLD). Local government areas (LGAs) and suburbs were utilized as geographical units for the estimation using Bayesian mapping approach.

**Results:**

Of the 78 LGAs, 21 showed high-oral cancer incidence as measured using higher median smoothed incidence risk (SIR), above the state average. Specifically, nine LGAs within predominantly rural areas had SIR above 100% of the state average. Of these, only one LGA (Mount Isa City) had a median smoothed SIR and 95% CI of 2.61 (2.14–3.15) which was constantly above 100% of the state average. Furthermore, mortality risk estimated using smoothed mortality risk (SMR), were significantly higher than the state average in 31 LGAs. Seventeen LGAs had a median SMR above 100% of the state average while three LGAs had the highest overall, 3- and 5-year mortality risks. Considering the 95% credible interval which is indicative of the uncertainty around the estimates, three LGAs had the highest overall mortality risks—Yarrabah Aboriginal Shire (3.80 (2.16–6.39)), Cook Shire (3.37 (2.21–5.06)), and Mount Isa City (3.04 (2.40–3.80)).

**Conclusion:**

Bayesian disease mapping approach identified multiple incidence and mortality hotspots within regional areas of the Queensland. Findings from our study can aid in designing targeted public health screening and interventions for primary prevention of oral cancer in regional and remote communities.

## 1. Introduction

Cancer is one of the fast-growing chronic diseases affecting people worldwide. An estimated 47% increase in new cancer cases has been projected to be diagnosed with incidence reaching 28.4 million cases worldwide by 2040 [1]. In Australia, cancer incidence is estimated to grow around 22% with 1.7 million new cases expected to be diagnosed as early as 2031 [2]. This is particularly concerning since cancer was recently identified as the greatest disease burden (18%) in Australia and responsible for approximately 881,000 disability-adjusted life years (DALY) [2, 3]. Furthermore, of the various risk factors implicated in cancer, use of tobacco has been noted to have the highest impact, being responsible for one-fifth of the cancer burden in Australia [2]. Tobacco and alcohol remain the leading etiological factors implicated in various types of malignancies in humans, specifically oral cancers while human papilloma viruses (HPV) are implicated in oropharyngeal cancers [4, 5].

Oral cavity cancer and oropharyngeal cancer commonly present as squamous cell carcinoma (termed OSCC or OPSCC, respectively) and have been recognized as the 14th most common malignancy worldwide [1, 6]. The term oral cancer generally includes oral cavity cancers affecting the anterior part of the tongue, floor of the mouth, buccal or labial mucosa, gingiva, hard palate, and retromolar area, while tumors involving the tongue base, lingual and palatal tonsils, soft palate, and oropharyngeal walls are categorized as oropharyngeal cancers. Although the overall incidence and mortality rates of head and neck cancer (including lip) have stabilized over the last two decades in Australia, the most concerning trends noted are a drastic increase in both incidence and mortality in population aged 50 years or over [2]. Oral cancer 5-year survival rates have improved marginally from 55% (1992) to 64% (2017) for head and neck cancer but the lowest improvement has been recorded in males for the oral cancer in the same time period (44.3% to 53.7%) [2]. This is particularly challenging health issue in light of recent WHO report predicting that by 2050, one-fifth of world population will be composed of people aged 60 or over [7]. Furthermore, over the past two decades, the population aged 85 years and over increased by 110%, compared with the total population growth of 35% in Australia increasing the probability and proportion of population affected by chronic health conditions such as oral cancer [8].

Cancer incidence is further compounded by low-socioeconomic status, poor nutrition, and regionality or remoteness. Particularly, cancer has been reported to be higher in regional and remote locations, when compared to major cities in Australia [9]. Average oral cancer 5-year survival rate for indigenous Australians is 23% lower than nonindigenous Australians [10]. Also, gender disparity is evident in oral cancer and incidence can be attributed to habits such as smoking and drinking alcohol [11]. Behavioral and lifestyle factors have been very widely implicated in oral cancer especially in rural and remote areas mainly due to lack of awareness around the risk factors for oral cancer [6, 12].

The spatial epidemiological approaches used to identify and quantify variations and patterns within a defined population residing in a geographical location are termed disease mapping. This approach can aid in identifying communities with higher risk of disease so that resources and targeted public health screening and healthcare interventions can be planned. This mapping approach has not been used to identify oral cancer risk in any states of Australia previously. Hence, this retrospective disease mapping study was undertaken to identify the oral cancer trends and patterns within the Queensland state in Australia using existing local government areas (LGA) and suburbs as spatial units.

## 2. Materials and Methods

Queensland state cancer registry (QCR) dataset for the period 1982–2018 was obtained to evaluate the oral and oropharyngeal cancer trends over time. The QCR is one of the largest population-based cancer registries in Australia is the custodian of all cancer-related data (excluding basal cell carcinomas) in Queensland. QCR collects detailed information from the healthcare providers including cancer patients' demographic details, cancer diagnosis, hospitalization, and mortality information through a mandatory QCR notification form [13]. It operates under the Public Health Act (PHA) 2005 and holds the most comprehensive cancer data sets to provide an overview of cancer in Queensland. Ethical approval was obtained from the James Cook University Human Research Ethics Committee (# H8609) and further approval under the PHA 2005 was provided by Queensland health. Dataset was received from the National Cancer Registry as a deidentified and password protected spreadsheet. All data have been managed under the Australian code for the responsible conduct of research.

### 2.1. Data Analysis

Descriptive statistics were conducted and presented as tables and in text. Oral cancer was broadly classified based on their location into oral cavity and oropharyngeal cancers. Oral cavity cancers were those involving the anterior tongue, floor of the mouth, buccal or labial mucosa, gingiva, hard palate, and retromolar area while tumors involving the tongue base, lingual and palatal tonsils, soft palate, and oropharyngeal walls were categorized as oropharyngeal cancers. Outcomes for mapping included the incidence, overall mortality, 3-year mortality, and 5-year mortality of oral cancer in Queensland, Australia. From the QCR cohort dataset, the spatial units that were extracted and included in analysis were suburbs and localities (SSC) data. These SSCs were adjusted to the boundaries used in the 2016 Australian census which meant that a total of 3,262 SSCs were used and mapped. Furthermore, this was used to generate higher area data at the level of the LGAs (*n* = 78).

LGA and SSC general population for the estimation of expected incidence and mortality of oral cancer comprised those aged 15 years and above according to the 2016 census data obtained from the Australian Bureau of Statistics (ABS) [14]. For SSC populations without residents in the ABS data, uninformative expected incidence/mortality values were used. Adjacency matrix for determining LGA and SSC areas with common boundaries was obtained using GeoDa (v1.20) according to the rook definition using first-order weighting. Only one LGA (Mornington Shire) had no neighbors following computation and the closest neighbor (Burke Shire) was manually assigned before spatial modeling. For the SSC maps, 42 islands did not have neighbors assigned automatically, and these were also input manually.

The hierarchical Bayesian approach was used to determine the adjusted incidence and mortality using the Gibbs sampler which is a type of Markov Chain Monte Carlo method for sampling from a probability distribution. Conditional autoregressive priors proposed by Besag, York, and Mollie (BYM) were specified within the model for spatial smoothing of estimates. Only a single Markov chain was run for all outcome estimates and the convergence of the chain was assessed using visual plots (trace, density, and autocorrelation) and Geweke diagnostic test. In total, 500,000 iterations were performed with every 20th iteration kept. The initial 60% of total iterations were used as burn-in to ensure convergence of all nodes while the latter 40% were kept for obtaining the smoothed estimates. Median smoothed incidence and mortality relative risks were used to construct choropleth maps using geometric data of LGAs and SSCs obtained from the Queensland Government Open Data Portal and ABS 2016 Census Data Packs [14–16]. Spatial clustering/variation was assessed using the Global and Local Moran's *I* test with probability values estimated with Monte Carlo randomization at 99,999 permutations. To identify spatial clusters and outliers, the lower limit of the 95% credible interval of the median risk estimation was used to select areas with significantly high and low risks for oral cancer incidence and mortality. Probability values below 5% were considered statistically significant. Descriptive statistics, convergence analysis, spatial modeling, and cancer maps were performed using SPSS v27, R statistical software, OpenBUGS, and GeoDa v1.20.

## 3. Results

### 3.1. Patient Cohort

In total, 9,887 patients with oral and oropharyngeal cancers were diagnosed between 1982 and 2018. Two patients were below 15 years and were excluded from further analysis. The age of included patients was from 18 to 105 years with a median age (IQR) of 62 (54–70) years with no oral cancer cases recorded in the age group of 15–17 years. Most patients were 65 years or older (56.3%) and more males than females (71.5% vs. 28.5%) were present in the cohort (Table 1). Slightly more patients had oral cavity tumors (52.1%) which were often moderately differentiated (49.4%). As of May 1, 2022, only 40.7% of the patients were alive and patients' survival time ranged from <1 month to 478 months with a median survival time (IQR) of 60 (17–122) months.

### 3.2. Incidence Risk Mapping

**Figure 1:**
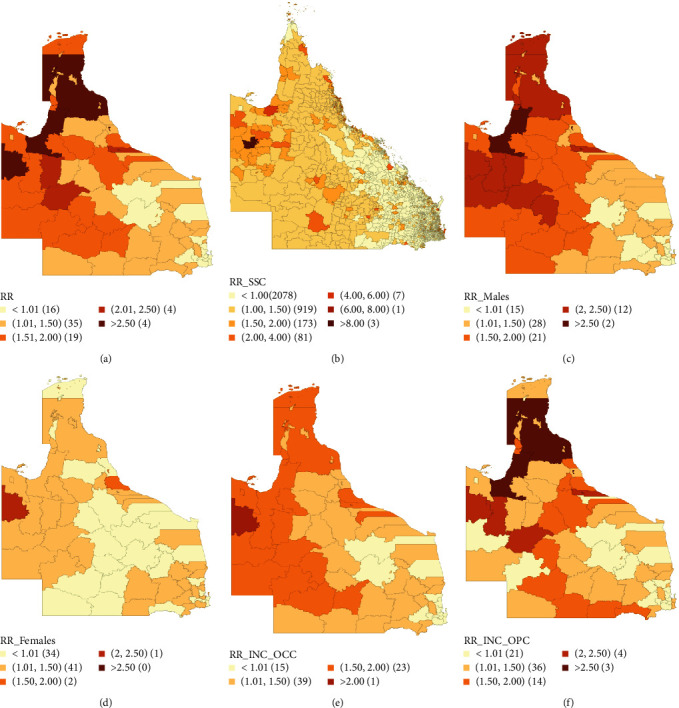
Bayesian smoothed median estimate maps for (a) oral cancer incidence risks in 78 local government areas, (b) oral cancer incidence risks in 3,262 state suburbs and localities, (c) oral cancer incidence risks in 78 local government areas among males, (d) oral cancer incidence risks in 78 local government areas among females, (e) incidence risks in 78 local government areas for oral cavity cancers, and (f) incidence risks in 78 local government areas for oropharyngeal cancers.

Nine thousand eight hundred eighty-five patients were used for oral cancer incidence mapping. The median smoothed standardized incidence ratio (SIR) estimated was from 0.77 to 2.91 across all the LGAs with the distribution of the relative risk in comparison to the Queensland average (SIR = 1.00) shown in Figure 1(a). Of the 62 LGAs with higher-than-average risk, 30 LGAs were statistically significant according to the estimation of their 95% credible intervals (95% CI) (Table S1). Twenty-one LGAs had high-oral cancer incidence risk between 1% and 100% above the state average while nine LGAs had a higher median smoothed SIR above 100% of the state average. Of these nine areas, only one LGA (Mount Isa City) had a median smoothed SIR and 95% CI of 2.61 (2.14–3.15) which was constantly above 100% of the state average.

Further spatial modeling done at the level of the Queensland suburbs (SSC; *n* = 3262) is plotted in the median SIR maps in Figure 1(b). Median smoothed SIRs ranged from 0 to 11.62. Overall, 107 suburbs with significantly higher median smoothed SIRs than the state-wide average was observed (Table S1). Fifteen suburbs had higher median smoothed SIRs and 95% credible interval above 100% with Mount Isa (11.62 (5.31–23.95)), Bundaberg Central (11.07 (5.84–19.47)), and Rockhampton city (9.60 (7.07–12.55)) being the three suburbs with the highest incidence risks.

Choropleth maps of oral cancer incidence risks by gender are presented in Figures 1(c) and 1(d). Generally, higher median SIRs were observed for males than females across most LGAs. For males, 34 LGAs had significantly higher oral cancer incidence risks, 14 of which had median smoothed SIRs above 100%. Carpentaria Shire had the highest median SIR (95% CI) of 2.72 (1.81–4.28) for males. Furthermore, the median smoothed SIR for females was significantly higher than the state average in only three LGAs—Mount Isa City (2.39 (1.61–3.44)), Cassowary Coast Regional Council (1.69 (1.21–2.32)), and Bundaberg Regional Council (1.29 (1.04–1.59)). However, none of the LGAs with the highest median risks (>100% above state average) for both genders had 95% credible intervals in the same range (Table S1).

Median smoothed SIR maps according to the tumor site (oral cavity vs oropharynx) are presented in Figures 1(e) and 1(f). The incidence risks of oral cavity cancer and oropharyngeal cancer ranged from 0.77–2.77 to 0.64–3.76, respectively (Table S1). Eighteen LGAs had significantly increased oral cavity cancer incidence risk compared to the state average with only Mount Isa City having a median smoothed SIR and 95% CI that are above 100% higher than the average risk (2.77 [2.13–3.55]). For oropharyngeal cancers, 20 LGAs had markedly higher-than-state-average risks with 7 LGAs having median smoothed SIRs increased above 100%. Of these seven areas, only Yarrabah Aboriginal Shire had consistently increased estimates according to their median SIRs and 95% CI (3.76 (2.09–6.61)).

**Figure 2:**
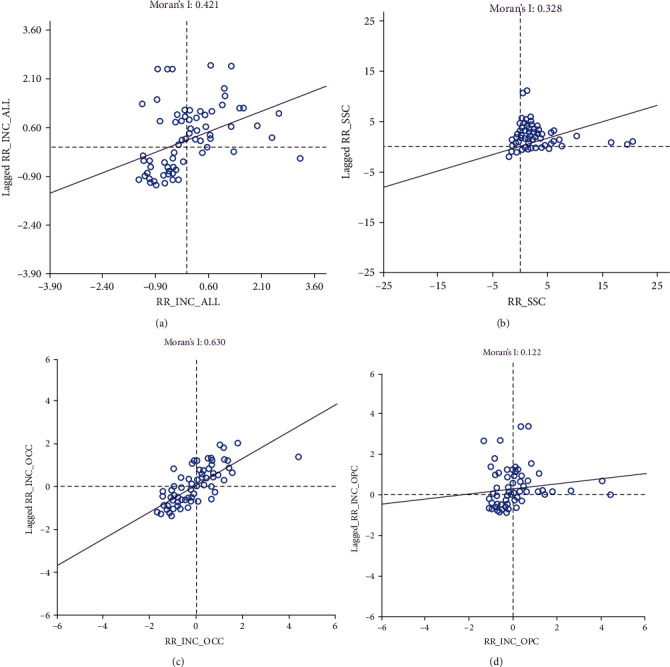
Scatter plots for global Moran's *I* estimation for (a) median smoothed standardized incidence ratios of oral cancer in 78 local government areas, (b) median smoothed standardized incidence ratios of oral cancer in 3,262 state suburbs and localities, (c) median smoothed standardized incidence ratios of oral cavity cancer in 78 local government areas, and (d) median smoothed standardized incidence ratios of oropharyngeal cancer in 78 local government areas.

Autocorrelation analysis scatter plots are shown in Figure 2. The global Moran's *I* statistic for the median SIR estimation of oral cancer at the LGA level was 0.42 with a *p*-value of <0.001. This means that the LGAs with high and/or low-median SIRs were more spatially clustered than would be expected if the underlying spatial processes were at random. Likewise, there was a significant positive spatial autocorrelation (0.33) for the median SMRs at the level of the SSC (*p* < 0.001). When the median SIRs at the LGA level were stratified by the tumor location, oral cavity cancer incidence risks also had significant spatial clustering with a global Moran's *I* statistic of 0.63 (*p* < 0.001). Although median SIRs for oropharyngeal cancers displayed a positive global spatial autocorrelation (0.12), this was not statistically significant (*p*=0.051).

**Figure 3:**
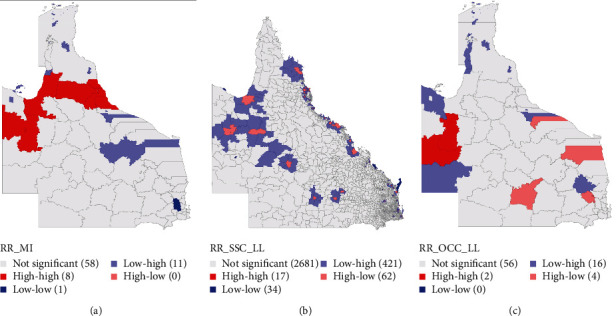
Local index of spatial autocorrelation maps for (a) significant median smoothed standardized incidence ratios of oral cancer in 78 local government areas, (b) significant median smoothed standardized incidence ratios of oral cancer in 3,262 state suburbs and localities, and (c) significant median smoothed standardized incidence ratios of oral cavity cancer in 78 local government areas.

Local index of spatial autocorrelation (LISA) map showed eight LGAs (and their neighbors) formed significant high-risk clusters (Mount Isa City, Cloncurry Shire, Carpentaria Shire, Mareeba Shire, Douglas Shire, Cairns, Cassowary Coast, and Tablelands Regional Councils (Figure 3(a)). Furthermore, only one significant low-risk cluster involving the Somerset Regional Council and its neighbors was observed. However, 11 spatial outliers were identified which represented areas with low risks surrounded by the neighbors with high risks of oral cancer incidence.

At the SSC level, significant high-risk and low-risk clusters were identified around 17 and 34 SSCs, respectively (Figure 3(b)). These high-risk SSCs were Bellara, Bongaree, Boondall, Clayfield, Currajong, Gulliver, Hyde Park, Manunda, Nelly Bay, Newtown, Parramatta Park, Pimlico, Redcliffe, Westcourt, Wooloowin, Woorin, and Wynnum. Likewise, 421 SSCs with a low risk of oral cancer incidence had high-risk neighbors while 62 clusters with high-risk incidence estimates were within areas with low-risk estimates.

The LISA map for oral cavity cancer incidence risks is shown in Figure 3(c). Two LGAs and their neighbors, i.e., Boulia and Cloncurry Shires were high-risk clusters. Also, 16 LGAs were low–high spatial outliers while four LGAs were high–low spatial outliers.

### 3.3. Mortality Risk Mapping

**Figure 4:**
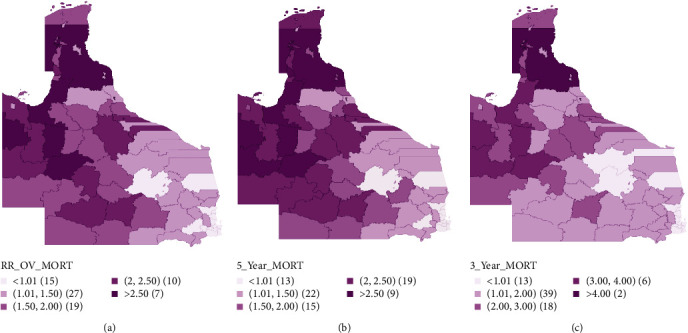
Bayesian smoothed median estimate maps for (a) oral cancer overall mortality risks in 78 local government areas, (b) oral cancer 5-year mortality risks in 78 local government areas, and (c) oral cancer 3-year mortality risks in 78 local government areas.

Five thousand eight hundred fifty-nine patients died within the period in review with 3,482 patients dying within 3 years and 617 more deaths within 5 years and the rest (1,760 deaths) occurring after 5 years postdiagnosis. Median smoothed standardized mortality ratios (SMR) maps based on lifetime mortality for patients with oral cancer in the Queensland LGAs are shown in Figure 4(a). Estimated median SMRs ranged from 0.65 to 3.80 and were significantly higher than the state average (SMR = 1.00) in 31 LGAs. Seventeen LGAs had a median SMR above 100% of the state average while 15 LGAs had a median SMR within 100% of the state average (Table S1). Considering the 95% credible interval which is indicative of the uncertainty around the estimates, three LGAs had the highest overall mortality risks—Yarrabah Aboriginal Shire (3.80 (2.16–6.39)), Cook Shire (3.37 (2.21–5.06)), and Mount Isa City (3.04 (2.40–3.80)).

The choropleth map for median SMRs based on 5-year mortality is presented in Figure 4(b). Estimated median SMRs were from 0.63 to 5.32 and 36 LGAs had significantly higher than state average mortality risks. The highest 5-year mortality risks were also observed in the same LGAs as the overall mortality risks with Yarrabah Aboriginal Shire, Cook Shire, and Mount Isa City having a median SMR (95% CI) of 5.32 (2.92–9.02), 4.18 (2.66–6.43), and 3.58 (2.75–4.57), respectively (Table S1). Similar trends were also observed in the 3-year median SMR map (Figure 4(c)) with 34 LGAs having significantly higher-than-state-average oral cancer mortality risks. The highest 3-year mortality risk was also estimated in Yarrabah Aboriginal Shire, Cook Shire, and Mount Isa City with a median SMR (95% CI) of 5.74 (3.07–9.98), 4.32 (2.72–6.76), and 3.84 (2.92–4.96), respectively (Table S1).

**Figure 5:**
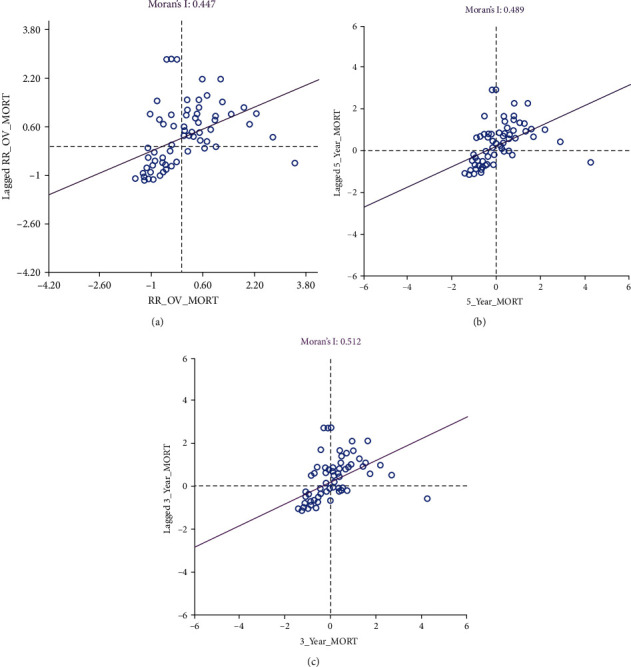
Scatter plots for global Moran's *I* estimation for (a) median smoothed standardized overall mortality ratios of oral cancer in 78 local government areas, (b) median smoothed standardized 5-year mortality ratios of oral cancer in 78 local government areas, and (c) median smoothed standardized 3-year mortality ratios of oral cancer in 78 local government areas.

**Figure 6:**
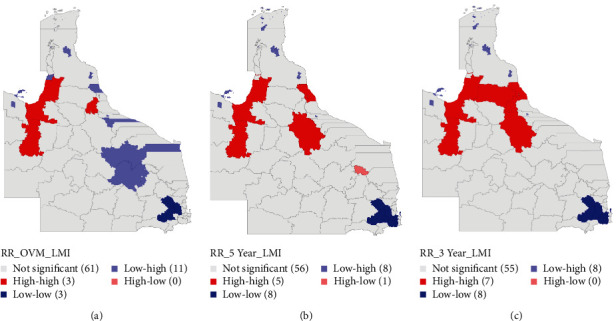
Local index of spatial autocorrelation maps for (a) Significant median smoothed standardized overall mortality ratios of oral cancer in 78 local government areas, (b) significant median smoothed standardized 5-year mortality ratios of oral cancer in 78 local government areas, and (c) significant median smoothed standardized 3-year mortality ratios of oral cancer in 78 local government areas.

Global Moran's *I* scatter plots are displayed in Figure 5. All three measures of mortality risks (overall mortality, 5-year mortality, and 3-year mortality) had significant positive spatial autocorrelation (0.45–0.51, *p* < 0.001). LISA maps for overall mortality showed three significant high–high (Carpentaria Shire, Cloncurry Shire, and Tablelands Regional Council) and three significant low–low (Somerset, South Burnett, and Toowoomba Regional Councils) lifetime mortality spatial clusters (Figure 6(a)). In addition to Carpentaria and Cloncurry Shires, Cairns, and Charters Towers Regional Councils, as well as Douglas Shire, formed significant high-risk clusters with their LGA neighbors when mortality within 5 years of diagnosis was considered (Figure 6(b)). Also, seven high–high (Cairns, Carpentaria, Charters Towers, Cloncurry, Douglas, Tablelands, and Mareeba) LGA clusters and eight low–low LGA clusters were identified for mortality within 3 years of diagnosis.

## 4. Discussion

Increasing incidence of cancer in a population living within a geographic location is a significant health challenge. Bayesian statistical approach is a reliable and established methodology used to map oral cancer incidence and mortality risk within well-defined geographic regions. This study was first Australian study to map the oral and oropharyngeal cancer in the Queensland state, Australia.

Of the Australian states and Territories, Queensland has been reported to have the highest incidence of oropharyngeal cancer, i.e., 3.2 cases per 100,000 persons compared to national average of 2.7 cases per 100,000 persons [17]. In our study evaluating the incidence data spanning between 1982 and 2018, 9,885 patients were diagnosed with oral cancer in adults. At least nine LGAs were identified to have more than double the SIR, in comparison to QLD average including the predominately aboriginal areas (LGAs) Yarrabah Aboriginal Shire, Carpentaria Shire, Mount Isa City, Cook Shire, Winton Shire, Wujal Wujal Aboriginal Shire, McKinlay Shire, Hinchinbrook Shire, and Longreach Regional Shire. This is particularly concerning since 76.5% of the populated areas in the above (nine) LGAs are classified as remote or very remote and have five times the Aboriginal and Torres Strait Islander population in comparison to whole of Queensland [18].

Various demographic factors have been identified as risk factors for oral cancer. Male gender has been reported to be a significant risk factor with higher proportion affected in various populations in Australia and across the world [11, 19, 20]. In our study, close to 72% of the diagnosed patients were males. Thirty-four LGAs were identified in our study with higher incidence risk for males, with Carpentaria Shire showing highest risk for males. This may be due to the higher prevalence of known risk behaviors including smoking and alcohol consumption in Australian males, particularly in the case of oral cavity cancers [4, 5, 21]. Although the proportion of males who smoke daily has decreased from 15% (2016) to 13% in (2019), the greatest difference between genders was noted in 40–49-year-olds, with more males smoking daily (18.4%) than women (13.4%) [21]. This may, in part, explain the higher proportion (97%) of oral cancer patients diagnosed were aged over 40 years in our study.

Regionality plays a significant role in prevalence of chronic health issues in Australia [6, 12, 22, 23]. This may be due higher risk behaviors, lack of awareness around healthy practices, level of education, and limited access to health care facilities [23–25]. In our study, inner regional suburbs Bundaberg Central and Rockhampton City along with Mount Isa which is classified as very remote suburb had the highest incidence risks [26]. Previously, three Australian studies conducted in regional Western Australia reported that an increasing number of oral cancer cases diagnosed in regional and remote areas are classified as in advanced stages of the disease [24, 25, 27]. Reasons for delay in diagnosis were reported to be regional isolation, lack of knowledge about the risk factors and symptoms, tobacco and alcohol abuse and HPV related oropharyngeal squamous cell carcinoma [27–29]. Additionally, the proportion of people living in the lowest socioeconomic areas who smoked daily was almost four times higher than in the proportion of people in highest socioeconomic areas (18.1% compared with 5.0%) [10].

Our mapping identified three LGAs, Yarrabah Aboriginal Shire, Cook Shire, and Mount Isa city with highest overall mortality risks. Furthermore, the highest mortality risks at 3 and 5 years was also noted in the above LGAs. Demographic profile of residents within these regions varied significantly with Yarrabah having a predominately Indigenous people (97.3%) to Mount Isa homing 72% of nonindigenous persons (2016) [18]. LISA mapping is known to provide a statistic for each location with significance and establish a proportional relationship between the local and global statistics [30]. In our study, LISA maps indicated Carpentaria Shire, Cloncurry Shire, and Tablelands Regional Council LGAs were classed as high–high (high-risk LGA adjacent to high-risk LGA) clusters for life time mortality. [31]. Within these regions, Tablelands (*R*) LGA had the largest percentage of persons aged 65+ (25.2%) compared to 16% for the whole of QLD and Carpentaria having 41% Indigenous people residing in the area. Additionally, Carpentaria has one of the highest rates of homelessness with 1 in 50 people listed as homeless along with 40% of the residents deemed to be most disadvantaged and 26% unemployed [31]. All of the above demographic and socioeconomic factors can contribute to increased incidence and mortality of oral and oropharyngeal cancers.

Bayesian modeling has been widely used in assessing spatiotemporal variations of various diseases including SARS and various types of cancers globally [19, 32–43]. However, minimal studies exist in the spatial mapping of oral and oropharyngeal cancer. In a study focusing on oral and oropharynx cancer mortality, it was reported that 4 out of 5 deaths were in males and geographically clustered in southeast and southern regions of Brazil [38]. Similar to the current study, our previous study on Hong Kong population successfully identified of hotspots or case cluster for incidence and higher mortality risks within two districts of Hong Kong Island [19]. It must be acknowledged that the modeling compares the risk to the larger unit (state or country) average incidence or mortality rates to arrive at a subunit (locality) specific risk levels (currently under review “Interpretation of Bayesian maps”) Additionally, the models used in mapping may include many structural and functional assumptions at different levels of the hierarchy [44]. The effect of such assumptions may be noted in the median smoothed SIR values for female from Bundaberg regional council, specifically the lower limit being just above the state (i.e., 1.04 compared to 1 for the state). However, this is still considered significant since the median SIR is 1.29. Additionally, it is evident that LGAs with highest median risks had a variable range of values for both genders. This may be attributed to the variation within the geographic area and the overall trend of male predisposition for oral and oropharyngeal cancer.

There are some limitations identified within this retrospective study. The focus of this paper was limited to the cases histologically diagnosed as squamous cell carcinoma and other diagnosis were excluded from the analysis. Additionally, the definition and criteria for tumor grade varied over the study period (36 years) which may affect the grouping and severity of cancer. Dataset obtained from QCR was limited to the period between January 1, 1982, and December 31, 2018. Data prior to 1982 were not held by a central registry and were not accessible. Additionally, the databases did not record lifestyle behaviors such as smoking and alcohol history in the patients diagnosed with cancer.

## 5. Conclusions

This paper confirms the effective identification of at-risk population for oral cancer within the regional and remote areas of Queensland state using Bayesian disease mapping approach. The finding of our study can be utilized to design targeted prevention programs and interventional strategies to be delivered to communities living in areas identified as hotspots.

## Figures and Tables

**Table 1:** Patient cohort characteristics.

Variable	Category	*N* (%)
Age	<40 years	283 (2.9)
	40–64 years	4,036 (40.8)
	≥65 years	5,566 (56.3)

Gender	Female	2,815 (28.5)
	Male	7,070 (71.5)

Tumor site	Oral cavity	5,151 (52.1)
	Oropharynx	4,578 (46.3)
	Not specified	156 (1.6)

Tumor grade	Well-differentiated	1,140 (11.5)
	Moderately differentiated	4,888 (49.4)
	Poorly differentiated	2,359 (23.9)
	Undifferentiated	33 (0.3)
	Unknown	1,465 (14.9)

Status	Alive	4,026 (40.7)
	Dead	5,859 (59.3)

## Data Availability

The data that support the findings of this study are available from Queensland Cancer Registry (QCR). Restrictions apply to the availability of these data, which were used under license for this study. Data are available from the authors with the permission of QCR.

## Supplementary Materials

Supplementary Materials Table S1: smoothed standardized ratios based on local government areas (LGA) and suburbs within Queensland.
