# Supplementary material for: Bayesian Disease Mapping to Identify High-Risk Population for Oral Cancer: A Retrospective Spatiotemporal Analysis
Source: Int J Dent. 2023 Nov 2;2023:3243373. doi: 10.1155/2023/3243373 (PMC10635753; doi:10.1155/2023/3243373)
Supplement: Supplementary Materials — Table S1: smoothed standardized ratios based on local government areas (LGA) and suburbs within Queensland. [file 3243373.f1.pdf]

Table of Contents

Overall Incidence (SIR) based on Local Government Area
Incidence (SIR) in Males based on Local Government Area
Incidence (SIR) in Females based on Local Government Area
Incidence (SIR) of Oral Cavity Carcinoma (OCC) based on Local Government Area
Incidence (SIR) of Oro-pharyngeal carcinoma (OPC) based on Local Government Area
Overall Mortility (SMR) based on Local Government Area
5 Year Mortility (SMR) based on Local Government Area
3 Year Mortility (SMR) based on Local Government Area
Overall Incidence (SIR) based on Suburb

Abbreviations:

INC - Incidence
OV - Overall
SIR - Standardized Incidence Ratio
SMR - Standardized Mortility Ratio
LGA - Local Government Area
LL - Lower Limit
UL- Upper limit

LGA	SIR	LL	Median	UL	Population
Aurukun Shire	RR[55]	0.77	1.45	2.58	896
Balonne Shire	RR[56]	0.79	1.22	1.83	3433
Banana Shire	RR[57]	0.88	1.18	1.55	11152
Barcaldine Regional	RR[58]	0.90	1.40	2.11	2281
Barcoo Shire	RR[59]	0.83	1.53	2.71	225
Blackall Tambo Regional	RR[60]	0.94	1.52	2.39	1570
Boulia Shire	RR[61]	**1.06**	**1.92**	**3.42**	352
Brisbane City	RR[62]	0.88	0.92	0.96	931878
Bulloo Shire	RR[63]	0.70	1.35	2.50	297
Bundaberg Regional	RR[64]	**1.12**	**1.26**	**1.42**	76491
Burdekin Shire	RR[65]	**1.27**	**1.62**	**2.04**	14040
Burke Shire	RR[66]	**1.04**	**1.88**	**3.32**	276
Cairns Regional	RR[67]	**1.13**	**1.25**	**1.37**	124800
Carpentaria Shire	RR[68]	**1.74**	**2.63**	**4.03**	1553
Cassowary Coast Regional	RR[69]	**1.42**	**1.72**	**2.05**	23460
Central Highlands Regional	RR[70]	0.64	0.84	1.08	21079
Charters Towers Regional	RR[71]	**1.28**	**1.68**	**2.18**	9321
Cherbourg Aboriginal Shire	RR[72]	0.42	0.92	1.85	805
Cloncurry Shire	RR[73]	**1.24**	**1.90**	**2.80**	2418
Cook Shire	RR[74]	**1.77**	**2.54**	**3.60**	3442
Croydon Shire	RR[75]	0.85	1.58	2.83	237
Diamantina Shire	RR[76]	0.92	1.74	3.27	237
Doomadgee Aboriginal Shire	RR[77]	0.58	1.28	2.51	861
Douglas Shire	RR[78]	**1.04**	**1.40**	**1.84**	9781
Etheridge Shire	RR[1]	0.94	1.58	2.61	647
Flinders Shire	RR[2]	0.87	1.46	2.33	1270
Fraser Coast Regional	RR[3]	**1.10**	**1.24**	**1.39**	84462
Gladstone Regional	RR[4]	0.69	0.84	1.00	47696
Gold Coast City	RR[5]	0.87	0.93	0.98	454869
Goondiwindi Regional	RR[6]	0.90	1.25	1.69	8360
Gympie Regional	RR[7]	0.76	0.92	1.10	40504
Hinchinbrook Shire	RR[8]	**1.57**	**2.04**	**2.62**	9178
Hope Vale Aboriginal Shire	RR[9]	0.51	1.15	2.28	658
Ipswich City	RR[10]	0.83	0.92	1.02	147805
Isaac Regional	RR[11]	0.65	0.88	1.16	15636
Kowanyama Aboriginal Shire	RR[12]	0.94	1.73	3.08	690
Livingstone Shire	RR[13]	**1.08**	**1.31**	**1.57**	29422
Lockhart River Aboriginal Shire	RR[14]	0.54	1.23	2.52	488
Lockyer Valley Regional	RR[15]	0.69	0.86	1.06	31066
Logan City	RR[16]	0.70	0.77	0.84	234853
Longreach Regional	RR[17]	**1.35**	**2.00**	**2.92**	2961
Mackay Regional	RR[18]	**1.04**	**1.17**	**1.31**	91200
Mapoon Aboriginal Shire	RR[19]	0.67	1.39	2.75	225
Maranoa Regional	RR[20]	**1.02**	**1.36**	**1.80**	9908
Mareeba Shire	RR[21]	**1.14**	**1.44**	**1.78**	17681
McKinlay Shire	RR[22]	**1.25**	**2.11**	**3.55**	659
Moreton Bay Regional	RR[23]	0.80	0.86	0.92	336169
Mornington Shire	RR[24]	0.80	1.65	3.15	797
Mount Isa City	RR[25]	**2.14**	**2.61**	**3.15**	14196

Murweh Shire	RR[26]	**1.09**	**1.62**	**2.36**	3404
Napranum Aboriginal Shire	RR[27]	0.69	1.34	2.40	665
Noosa Shire	RR[28]	0.95	1.12	1.32	43701
North Burnett Regional	RR[29]	0.84	1.16	1.57	8621
Northern Peninsula Area Regional	RR[30]	0.54	1.03	1.76	1769
Palm Island Aboriginal Shire	RR[31]	0.37	0.82	1.55	1665
Paroo Shire	RR[32]	0.78	1.31	2.12	1329
Pormpuraaw Aboriginal Shire	RR[33]	**1.08**	**1.99**	**3.65**	548
Quilpie Shire	RR[34]	**1.03**	**1.72**	**2.93**	644
Redland City	RR[35]	0.90	1.00	1.12	119406
Richmond Shire	RR[36]	0.77	1.40	2.36	644
Rockhampton Regional	RR[37]	**1.07**	**1.22**	**1.39**	63150
Scenic Rim Regional	RR[38]	0.79	0.97	1.18	32485
Somerset Regional	RR[39]	0.98	1.23	1.54	19566
South Burnett Regional	RR[40]	1.00	1.22	1.49	26057
Southern Downs Regional	RR[41]	0.89	1.10	1.34	28497
Sunshine Coast Regional	RR[42]	0.82	0.89	0.96	240988
Tablelands Regional	RR[43]	**1.17**	**1.44**	**1.76**	20322
Toowoomba Regional	RR[44]	0.83	0.93	1.03	128187
Torres Shire	RR[45]	**1.06**	**1.66**	**2.53**	2632
Torres Strait Island Regional	RR[46]	0.92	1.53	2.41	2952
Townsville City	RR[47]	0.96	1.06	1.16	149049
Weipa Town	RR[48]	0.55	1.02	1.70	2868
Western Downs Regional	RR[49]	0.89	1.11	1.35	26063
Whitsunday Regional	RR[50]	**1.01**	**1.24**	**1.50**	27657
Winton Shire	RR[51]	**1.49**	**2.34**	**3.82**	950
Woorabinda Aboriginal Shire	RR[52]	0.61	1.16	2.15	638
Wujal Wujal Aboriginal Shire	RR[53]	**1.13**	**2.16**	**4.34**	220
Yarrabah Aboriginal Shire	RR[54]	**1.78**	**2.91**	**4.58**	1692

LGA	SIR	LL	Median	UL	Population
Aurukun Shire	RR[55]	0.89	1.69	3.04	444
Balonne Shire	RR[56]	0.79	1.24	1.88	1753
Banana Shire	RR[57]	0.90	1.23	1.65	5770
Barcaldine Regional	RR[58]	0.98	1.52	2.28	1147
Barcoo Shire	RR[59]	0.94	1.68	2.93	132
Blackall Tambo Regional	RR[60]	**1.06**	**1.69**	**2.68**	776
Boulia Shire	RR[61]	**1.16**	**2.04**	**3.58**	192
Brisbane City	RR[62]	0.84	0.89	0.93	454152
Bulloo Shire	RR[63]	0.80	1.51	2.77	164
Bundaberg Regional	RR[64]	**1.06**	**1.23**	**1.42**	37236
Burdekin Shire	RR[65]	**1.34**	**1.74**	**2.24**	7078
Burke Shire	RR[66]	**1.13**	**1.99**	**3.46**	153
Cairns Regional	RR[67]	**1.24**	**1.39**	**1.54**	60894
Carpentaria Shire	RR[68]	**1.81**	**2.72**	**4.28**	800
Cassowary Coast Regional	RR[69]	**1.29**	**1.60**	**1.97**	11997
Central Highlands Regional	RR[70]	0.76	1.00	1.28	11191
Charters Towers Regional	RR[71]	**1.43**	**1.90**	**2.51**	4563
Cherbourg Aboriginal Shire	RR[72]	0.44	0.99	2.06	378
Cloncurry Shire	RR[73]	**1.33**	**2.03**	**3.00**	1398
Cook Shire	RR[74]	**1.67**	**2.38**	**3.45**	1903
Croydon Shire	RR[75]	0.98	1.76	3.12	122
Diamantina Shire	RR[76]	0.93	1.75	3.23	120
Doomadgee Aboriginal Shire	RR[77]	0.57	1.34	2.74	393
Douglas Shire	RR[78]	**1.21**	**1.65**	**2.20**	4913
Etheridge Shire	RR[1]	**1.06**	**1.74**	**2.82**	362
Flinders Shire	RR[2]	**1.03**	**1.67**	**2.67**	648
Fraser Coast Regional	RR[3]	**1.11**	**1.27**	**1.45**	41026
Gladstone Regional	RR[4]	0.73	0.90	1.09	24578
Gold Coast City	RR[5]	0.89	0.95	1.02	218486
Goondiwindi Regional	RR[6]	1.00	1.40	1.95	4159
Gympie Regional	RR[7]	0.75	0.93	1.14	19888
Hinchinbrook Shire	RR[8]	**1.54**	**2.06**	**2.72**	4670
Hope Vale Aboriginal Shire	RR[9]	0.58	1.33	2.67	345
Ipswich City	RR[10]	0.79	0.89	1.00	72236
Isaac Regional	RR[11]	0.79	1.06	1.38	8704
Kowanyama Aboriginal Shire	RR[12]	**1.11**	**2.04**	**3.74**	321
Livingstone Shire	RR[13]	**1.02**	**1.27**	**1.56**	14982
Lockhart River Aboriginal Shire	RR[14]	0.63	1.44	2.97	238
Lockyer Valley Regional	RR[15]	0.69	0.89	1.12	15349
Logan City	RR[16]	0.66	0.74	0.82	114884
Longreach Regional	RR[17]	**1.46**	**2.19**	**3.28**	1434
Mackay Regional	RR[18]	**1.08**	**1.23**	**1.40**	46084
Mapoon Aboriginal Shire	RR[19]	0.75	1.56	3.05	113
Maranoa Regional	RR[20]	**1.04**	**1.42**	**1.91**	5037
Mareeba Shire	RR[21]	**1.26**	**1.60**	**2.01**	9327
McKinlay Shire	RR[22]	**1.31**	**2.17**	**3.63**	362
Moreton Bay Regional	RR[23]	0.75	0.81	0.89	162423
Mornington Shire	RR[24]	0.80	1.73	3.48	376
Mount Isa City	RR[25]	**1.89**	**2.39**	**2.97**	7372

Murweh Shire	RR[26]	**1.26**	**1.88**	**2.81**	1663
Napranum Aboriginal Shire	RR[27]	0.74	1.44	2.59	342
Noosa Shire	RR[28]	0.94	1.15	1.39	20977
North Burnett Regional	RR[29]	0.74	1.06	1.46	4402
Northern Peninsula Area Regiona	RR[30]	0.62	1.21	2.09	835
Palm Island Aboriginal Shire	RR[31]	0.43	0.97	1.85	820
Paroo Shire	RR[32]	0.86	1.43	2.31	665
Pormpuraaw Aboriginal Shire	RR[33]	**1.14**	**2.11**	**3.93**	273
Quilpie Shire	RR[34]	**1.17**	**1.92**	**3.25**	336
Redland City	RR[35]	0.89	1.01	1.15	57690
Richmond Shire	RR[36]	0.90	1.59	2.64	336
Rockhampton Regional	RR[37]	**1.13**	**1.32**	**1.53**	30920
Scenic Rim Regional	RR[38]	0.72	0.92	1.15	15912
Somerset Regional	RR[39]	0.90	1.17	1.50	9828
South Burnett Regional	RR[40]	0.91	1.15	1.44	12940
Southern Downs Regional	RR[41]	0.88	1.12	1.40	13870
Sunshine Coast Regional	RR[42]	0.77	0.85	0.94	114598
Tablelands Regional	RR[43]	**1.14**	**1.45**	**1.82**	9880
Toowoomba Regional	RR[44]	0.77	0.88	1.00	61753
Torres Shire	RR[45]	**1.29**	**2.04**	**3.17**	1279
Torres Strait Island Regional	RR[46]	0.94	1.64	2.67	1506
Townsville City	RR[47]	0.95	1.06	1.19	74011
Weipa Town	RR[48]	0.60	1.13	1.92	1549
Western Downs Regional	RR[49]	0.77	1.00	1.25	13292
Whitsunday Regional	RR[50]	**1.08**	**1.34**	**1.65**	14285
Winton Shire	RR[51]	**1.42**	**2.22**	**3.58**	494
Woorabinda Aboriginal Shire	RR[52]	0.69	1.30	2.41	311
Wujal Wujal Aboriginal Shire	RR[53]	**1.28**	**2.43**	**5.03**	98
Yarrabah Aboriginal Shire	RR[54]	**1.55**	**2.69**	**4.53**	837

LGA	SIR	LL	Median	UL	Population
Aurukun Shire	RR[55]	0.45	1.00	2.06	456
Balonne Shire	RR[56]	0.59	1.03	1.75	1690
Banana Shire	RR[57]	0.59	0.94	1.42	5379
Barcaldine Regional	RR[58]	0.58	1.00	1.68	1141
Barcoo Shire	RR[59]	0.56	1.07	1.97	106
Blackall Tambo Regional	RR[60]	0.52	0.95	1.66	790
Boulia Shire	RR[61]	0.66	1.31	2.48	159
Brisbane City	RR[62]	0.93	1.01	1.08	477737
Bulloo Shire	RR[63]	0.49	1.00	1.95	134
Bundaberg Regional	RR[64]	**1.04**	**1.29**	**1.59**	39257
Burdekin Shire	RR[65]	0.69	1.08	1.63	6962
Burke Shire	RR[66]	0.67	1.35	2.58	120
Cairns Regional	RR[67]	0.76	0.93	1.13	63903
Carpentaria Shire	RR[68]	0.70	1.21	2.08	746
Cassowary Coast Regional	RR[69]	**1.21**	**1.69**	**2.32**	11469
Central Highlands Regional	RR[70]	0.37	0.65	0.98	9882
Charters Towers Regional	RR[71]	0.62	0.98	1.47	4763
Cherbourg Aboriginal Shire	RR[72]	0.41	0.99	2.21	429
Cloncurry Shire	RR[73]	0.67	1.25	2.12	1017
Cook Shire	RR[74]	0.77	1.28	2.34	1537
Croydon Shire	RR[75]	0.58	1.12	2.08	109
Diamantina Shire	RR[76]	0.65	1.27	2.55	119
Doomadgee Aboriginal Shire	RR[77]	0.56	1.36	3.21	461
Douglas Shire	RR[78]	0.51	0.88	1.39	4873
Etheridge Shire	RR[1]	0.58	1.05	1.82	291
Flinders Shire	RR[2]	0.54	1.00	1.72	627
Fraser Coast Regional	RR[3]	0.93	1.15	1.42	43437
Gladstone Regional	RR[4]	0.51	0.74	1.01	23115
Gold Coast City	RR[5]	0.79	0.88	0.99	236380
Goondiwindi Regional	RR[6]	0.49	0.86	1.37	4201
Gympie Regional	RR[7]	0.67	0.94	1.25	20627
Hinchinbrook Shire	RR[8]	0.89	1.37	2.13	4517
Hope Vale Aboriginal Shire	RR[9]	0.39	1.00	2.28	313
Ipswich City	RR[10]	0.83	0.99	1.18	75575
Isaac Regional	RR[11]	0.33	0.63	0.99	6939
Kowanyama Aboriginal Shire	RR[12]	0.50	1.05	2.09	369
Livingstone Shire	RR[13]	0.84	1.17	1.64	14439
Lockhart River Aboriginal Shire	RR[14]	0.40	1.01	2.33	251
Lockyer Valley Regional	RR[15]	0.58	0.85	1.18	15707
Logan City	RR[16]	0.74	0.86	1.00	119960
Longreach Regional	RR[17]	0.61	1.07	1.84	1535
Mackay Regional	RR[18]	0.74	0.94	1.18	45111
Mapoon Aboriginal Shire	RR[19]	0.47	1.06	2.28	107
Maranoa Regional	RR[20]	0.64	1.01	1.56	4870
Mareeba Shire	RR[21]	0.59	0.93	1.36	8361
McKinlay Shire	RR[22]	0.66	1.21	2.20	291
Moreton Bay Regional	RR[23]	0.86	0.98	1.10	173749
Mornington Shire	RR[24]	0.57	1.38	3.31	414
Mount Isa City	RR[25]	**1.61**	**2.39**	**3.44**	6814

Murweh Shire	RR[26]	0.47	0.87	1.46	1746
Napranum Aboriginal Shire	RR[27]	0.53	1.10	2.24	332
Noosa Shire	RR[28]	0.78	1.07	1.42	22726
North Burnett Regional	RR[29]	0.80	1.21	1.88	4214
Northern Peninsula Area Regior	RR[30]	0.46	0.98	1.91	930
Palm Island Aboriginal Shire	RR[31]	0.40	0.97	2.07	836
Paroo Shire	RR[32]	0.53	0.98	1.74	665
Pormpuraaw Aboriginal Shire	RR[33]	0.58	1.19	2.42	276
Quilpie Shire	RR[34]	0.52	0.97	1.72	309
Redland City	RR[35]	0.81	0.99	1.20	61712
Richmond Shire	RR[36]	0.57	1.08	1.94	304
Rockhampton Regional	RR[37]	0.72	0.94	1.21	32224
Scenic Rim Regional	RR[38]	0.76	1.05	1.43	16567
Somerset Regional	RR[39]	0.82	1.16	1.65	9734
South Burnett Regional	RR[40]	0.90	1.25	1.72	13115
Southern Downs Regional	RR[41]	0.71	1.01	1.40	14639
Sunshine Coast Regional	RR[42]	0.89	1.02	1.17	126390
Tablelands Regional	RR[43]	0.94	1.32	1.86	10440
Toowoomba Regional	RR[44]	0.88	1.07	1.27	66432
Torres Shire	RR[45]	0.42	0.88	1.65	1346
Torres Strait Island Regional	RR[46]	0.46	1.07	2.25	1450
Townsville City	RR[47]	0.86	1.04	1.23	75031
Weipa Town	RR[48]	0.40	0.93	1.89	1320
Western Downs Regional	RR[49]	0.90	1.25	1.73	12771
Whitsunday Regional	RR[50]	0.59	0.87	1.24	13373
Winton Shire	RR[51]	0.76	1.29	2.32	449
Woorabinda Aboriginal Shire	RR[52]	0.40	0.85	1.67	330
Wujal Wujal Aboriginal Shire	RR[53]	0.47	1.01	2.07	114
Yarrabah Aboriginal Shire	RR[54]	0.83	1.71	3.76	850

LGA	SIR	LL	Median	UL	Population
Aurukun Shire	RR[55]	0.73	1.36	2.46	896
Balonne Shire	RR[56]	0.63	1.01	1.53	3433
Banana Shire	RR[57]	0.99	1.35	1.87	11152
Barcaldine Regional	RR[58]	0.88	1.33	1.98	2281
Barcoo Shire	RR[59]	0.91	1.54	2.59	225
Blackall Tambo Regional	RR[60]	0.93	1.45	2.29	1570
Boulia Shire	RR[61]	**1.14**	**1.93**	**3.32**	352
Brisbane City	RR[62]	0.94	0.99	1.05	931878
Bulloo Shire	RR[63]	0.79	1.40	2.47	297
Bundaberg Regional	RR[64]	**1.06**	**1.25**	**1.47**	76491
Burdekin Shire	RR[65]	**1.14**	**1.54**	**2.08**	14040
Burke Shire	RR[66]	0.99	1.73	2.88	276
Cairns Regional	RR[67]	0.98	1.12	1.28	124800
Carpentaria Shire	RR[68]	**1.19**	**1.79**	**2.78**	1553
Cassowary Coast Regional	RR[69]	**1.19**	**1.53**	**1.95**	23460
Central Highlands Regional	RR[70]	0.71	0.98	1.28	21079
Charters Towers Regional	RR[71]	0.89	1.24	1.68	9321
Cherbourg Aboriginal Shire	RR[72]	0.48	1.01	2.00	805
Cloncurry Shire	RR[73]	**1.05**	**1.68**	**2.53**	2418
Cook Shire	RR[74]	**1.19**	**1.73**	**2.62**	3442
Croydon Shire	RR[75]	0.88	1.50	2.53	237
Diamantina Shire	RR[76]	0.98	1.73	3.11	237
Doomadgee Aboriginal Shire	RR[77]	0.72	1.55	3.10	861
Douglas Shire	RR[78]	0.84	1.22	1.71	9781
Etheridge Shire	RR[1]	0.91	1.43	2.27	647
Flinders Shire	RR[2]	0.82	1.33	2.07	1270
Fraser Coast Regional	RR[3]	**1.07**	**1.25**	**1.46**	84462
Gladstone Regional	RR[4]	0.66	0.85	1.06	47696
Gold Coast City	RR[5]	0.76	0.83	0.90	454869
Goondiwindi Regional	RR[6]	0.72	1.06	1.51	8360
Gympie Regional	RR[7]	0.77	0.98	1.22	40504
Hinchinbrook Shire	RR[8]	**1.14**	**1.58**	**2.21**	9178
Hope Vale Aboriginal Shire	RR[9]	0.59	1.27	2.52	658
Ipswich City	RR[10]	0.79	0.91	1.04	147805
Isaac Regional	RR[11]	0.59	0.86	1.18	15636
Kowanyama Aboriginal Shire	RR[12]	0.83	1.50	2.68	690
Livingstone Shire	RR[13]	**1.02**	**1.30**	**1.64**	29422
Lockhart River Aboriginal Shire	RR[14]	0.61	1.32	2.66	488
Lockyer Valley Regional	RR[15]	0.69	0.90	1.16	31066
Logan City	RR[16]	0.68	0.77	0.86	234853
Longreach Regional	RR[17]	**1.22**	**1.84**	**2.86**	2961
Mackay Regional	RR[18]	0.94	1.11	1.29	91200
Mapoon Aboriginal Shire	RR[19]	0.72	1.40	2.65	225
Maranoa Regional	RR[20]	0.92	1.29	1.78	9908
Mareeba Shire	RR[21]	**1.02**	**1.35**	**1.75**	17681
McKinlay Shire	RR[22]	**1.12**	**1.80**	**2.99**	659
Moreton Bay Regional	RR[23]	0.83	0.91	1.00	336169
Mornington Shire	RR[24]	0.84	1.75	3.51	797
Mount Isa City	RR[25]	**2.13**	**2.77**	**3.55**	14196

Murweh Shire	RR[26]	**1.05**	**1.56**	**2.40**	3404
Napranum Aboriginal Shire	RR[27]	0.77	1.40	2.49	665
Noosa Shire	RR[28]	0.79	1.00	1.25	43701
North Burnett Regional	RR[29]	0.87	1.21	1.68	8621
Northern Peninsula Area Regional	RR[30]	0.70	1.27	2.17	1769
Palm Island Aboriginal Shire	RR[31]	0.48	1.01	1.90	1665
Paroo Shire	RR[32]	0.81	1.30	2.06	1329
Pormpuraaw Aboriginal Shire	RR[33]	0.85	1.54	2.76	548
Quilpie Shire	RR[34]	0.98	1.57	2.58	644
Redland City	RR[35]	0.83	0.97	1.12	119406
Richmond Shire	RR[36]	0.86	1.43	2.31	644
Rockhampton Regional	RR[37]	0.97	1.17	1.40	63150
Scenic Rim Regional	RR[38]	0.72	0.94	1.20	32485
Somerset Regional	RR[39]	0.93	1.22	1.61	19566
South Burnett Regional	RR[40]	**1.05**	**1.34**	**1.71**	26057
Southern Downs Regional	RR[41]	0.85	1.10	1.41	28497
Sunshine Coast Regional	RR[42]	0.79	0.88	0.99	240988
Tablelands Regional	RR[43]	0.97	1.27	1.64	20322
Toowoomba Regional	RR[44]	0.87	1.00	1.15	128187
Torres Shire	RR[45]	**1.01**	**1.64**	**2.64**	2632
Torres Strait Island Regional	RR[46]	0.81	1.50	2.59	2952
Townsville City	RR[47]	0.97	1.11	1.26	149049
Weipa Town	RR[48]	0.69	1.28	2.20	2868
Western Downs Regional	RR[49]	0.80	1.04	1.34	26063
Whitsunday Regional	RR[50]	0.81	1.06	1.37	27657
Winton Shire	RR[51]	**1.16**	**1.77**	**2.78**	950
Woorabinda Aboriginal Shire	RR[52]	0.58	1.06	1.86	638
Wujal Wujal Aboriginal Shire	RR[53]	0.87	1.57	2.96	220
Yarrabah Aboriginal Shire	RR[54]	0.56	1.12	2.07	1692

LGA	SIR	LL	Median	UL	Population
Aurukun Shire	RR[55]	0.76	1.48	2.84	896
Balonne Shire	RR[56]	1.00	1.70	2.84	3433
Banana Shire	RR[57]	0.50	0.83	1.31	11152
Barcaldine Regional	RR[58]	0.71	1.31	2.35	2281
Barcoo Shire	RR[59]	0.40	0.97	2.21	225
Blackall Tambo Regional	RR[60]	0.74	1.47	2.78	1570
Boulia Shire	RR[61]	0.47	0.96	1.95	352
Brisbane City	RR[62]	0.79	0.84	0.90	931878
Bulloo Shire	RR[63]	0.39	1.24	3.41	297
Bundaberg Regional	RR[64]	**1.05**	**1.26**	**1.49**	76491
Burdekin Shire	RR[65]	**1.09**	**1.55**	**2.14**	14040
Burke Shire	RR[66]	0.59	1.43	3.40	276
Cairns Regional	RR[67]	**1.20**	**1.37**	**1.57**	124800
Carpentaria Shire	RR[68]	**1.80**	**3.57**	**6.63**	1553
Cassowary Coast Regional	RR[69]	**1.33**	**1.74**	**2.25**	23460
Central Highlands Regional	RR[70]	0.53	0.79	1.13	21079
Charters Towers Regional	RR[71]	**1.35**	**1.98**	**2.82**	9321
Cherbourg Aboriginal Shire	RR[72]	0.53	1.26	2.80	805
Cloncurry Shire	RR[73]	**1.06**	**2.01**	**3.61**	2418
Cook Shire	RR[74]	**1.76**	**2.82**	**4.45**	3442
Croydon Shire	RR[75]	0.68	1.40	2.81	237
Diamantina Shire	RR[76]	0.62	1.30	2.70	237
Doomadgee Aboriginal Shire	RR[77]	0.40	0.89	1.88	861
Douglas Shire	RR[78]	**1.01**	**1.58**	**2.35**	9781
Etheridge Shire	RR[1]	0.56	1.22	2.62	647
Flinders Shire	RR[2]	0.84	1.62	3.00	1270
Fraser Coast Regional	RR[3]	**1.03**	**1.23**	**1.45**	84462
Gladstone Regional	RR[4]	0.68	0.89	1.15	47696
Gold Coast City	RR[5]	0.96	1.04	1.13	454869
Goondiwindi Regional	RR[6]	**1.02**	**1.60**	**2.40**	8360
Gympie Regional	RR[7]	0.64	0.86	1.14	40504
Hinchinbrook Shire	RR[8]	**1.64**	**2.35**	**3.27**	9178
Hope Vale Aboriginal Shire	RR[9]	0.43	1.08	2.43	658
Ipswich City	RR[10]	0.82	0.96	1.11	147805
Isaac Regional	RR[11]	0.65	0.99	1.44	15636
Kowanyama Aboriginal Shire	RR[12]	0.78	1.57	3.14	690
Livingstone Shire	RR[13]	1.00	1.32	1.71	29422
Lockhart River Aboriginal Shire	RR[14]	0.50	1.06	2.15	488
Lockyer Valley Regional	RR[15]	0.65	0.90	1.20	31066
Logan City	RR[16]	0.69	0.79	0.89	234853
Longreach Regional	RR[17]	0.86	1.55	2.68	2961
Mackay Regional	RR[18]	**1.04**	**1.23**	**1.45**	91200
Mapoon Aboriginal Shire	RR[19]	0.59	1.23	2.45	225
Maranoa Regional	RR[20]	0.92	1.42	2.09	9908
Mareeba Shire	RR[21]	**1.04**	**1.48**	**2.03**	17681
McKinlay Shire	RR[22]	0.73	1.42	2.82	659
Moreton Bay Regional	RR[23]	0.71	0.79	0.88	336169
Mornington Shire	RR[24]	0.46	1.29	3.14	797
Mount Isa City	RR[25]	**1.53**	**2.09**	**2.79**	14196

Murweh Shire	RR[26]	0.72	1.31	2.28	3404
Napranum Aboriginal Shire	RR[27]	0.63	1.24	2.30	665
Noosa Shire	RR[28]	1.00	1.27	1.59	43701
North Burnett Regional	RR[29]	0.73	1.21	1.90	8621
Northern Peninsula Area Regiona	RR[30]	0.28	0.81	1.89	1769
Palm Island Aboriginal Shire	RR[31]	0.39	0.93	1.93	1665
Paroo Shire	RR[32]	0.75	1.55	2.98	1329
Pormpuraaw Aboriginal Shire	RR[33]	0.91	1.76	3.49	548
Quilpie Shire	RR[34]	0.79	1.51	2.91	644
Redland City	RR[35]	0.90	1.06	1.23	119406
Richmond Shire	RR[36]	0.61	1.26	2.48	644
Rockhampton Regional	RR[37]	**1.06**	**1.29**	**1.55**	63150
Scenic Rim Regional	RR[38]	0.71	0.97	1.28	32485
Somerset Regional	RR[39]	0.88	1.24	1.70	19566
South Burnett Regional	RR[40]	0.69	0.97	1.32	26057
Southern Downs Regional	RR[41]	0.80	1.10	1.46	28497
Sunshine Coast Regional	RR[42]	0.80	0.91	1.02	240988
Tablelands Regional	RR[43]	**1.16**	**1.57**	**2.08**	20322
Toowoomba Regional	RR[44]	0.71	0.84	0.99	128187
Torres Shire	RR[45]	0.71	1.36	2.47	2632
Torres Strait Island Regional	RR[46]	0.82	1.43	2.37	2952
Townsville City	RR[47]	0.87	1.00	1.15	149049
Weipa Town	RR[48]	0.23	0.64	1.43	2868
Western Downs Regional	RR[49]	0.89	1.20	1.59	26063
Whitsunday Regional	RR[50]	**1.06**	**1.38**	**1.78**	27657
Winton Shire	RR[51]	**1.09**	**2.15**	**4.36**	950
Woorabinda Aboriginal Shire	RR[52]	0.68	1.46	3.10	638
Wujal Wujal Aboriginal Shire	RR[53]	0.62	1.81	5.49	220
Yarrabah Aboriginal Shire	RR[54]	**2.09**	**3.76**	**6.61**	1692

LGA	SMR	LL	Median	UL	Population
Aurukun Shire	RR[55]	0.93	1.91	3.75	896
Balonne Shire	RR[56]	0.79	1.34	2.16	3433
Banana Shire	RR[57]	**1.03**	**1.45**	**2.01**	11152
Barcaldine Regional	RR[58]	**1.08**	**1.78**	**2.86**	2281
Barcoo Shire	RR[59]	0.90	1.82	3.55	225
Blackall Tambo Regional	RR[60]	**1.11**	**1.92**	**3.27**	1570
Boulia Shire	RR[61]	**1.20**	**2.37**	**4.61**	352
Brisbane City	RR[62]	0.94	0.99	1.05	931878
Bulloo Shire	RR[63]	0.77	1.64	3.36	297
Bundaberg Regional	RR[64]	**1.06**	**1.25**	**1.45**	76491
Burdekin Shire	RR[65]	**1.53**	**2.03**	**2.65**	14040
Burke Shire	RR[66]	**1.13**	**2.23**	**4.34**	276
Cairns Regional	RR[67]	**1.05**	**1.20**	**1.35**	124800
Carpentaria Shire	RR[68]	**1.55**	**2.55**	**4.17**	1553
Cassowary Coast Regional	RR[69]	**1.54**	**1.94**	**2.40**	23460
Central Highlands Regional	RR[70]	0.64	0.90	1.22	21079
Charters Towers Regional	RR[71]	**1.50**	**2.06**	**2.79**	9321
Cherbourg Aboriginal Shire	RR[72]	0.41	1.03	2.37	805
Cloncurry Shire	RR[73]	**1.22**	**2.06**	**3.27**	2418
Cook Shire	RR[74]	**2.21**	**3.37**	**5.06**	3442
Croydon Shire	RR[75]	0.90	1.83	3.61	237
Diamantina Shire	RR[76]	0.89	1.91	3.95	237
Doomadgee Aboriginal Shire	RR[77]	0.50	1.33	2.97	861
Douglas Shire	RR[78]	0.92	1.36	1.93	9781
Etheridge Shire	RR[1]	0.98	1.80	3.23	647
Flinders Shire	RR[2]	**1.05**	**1.88**	**3.23**	1270
Fraser Coast Regional	RR[3]	**1.11**	**1.29**	**1.49**	84462
Gladstone Regional	RR[4]	0.71	0.89	1.11	47696
Gold Coast City	RR[5]	0.77	0.84	0.91	454869
Goondiwindi Regional	RR[6]	0.79	1.20	1.77	8360
Gympie Regional	RR[7]	0.68	0.87	1.10	40504
Hinchinbrook Shire	RR[8]	**1.62**	**2.23**	**3.02**	9178
Hope Vale Aboriginal Shire	RR[9]	0.53	1.36	3.07	658
Ipswich City	RR[10]	0.70	0.81	0.93	147805
Isaac Regional	RR[11]	0.70	1.01	1.40	15636
Kowanyama Aboriginal Shire	RR[12]	0.95	1.97	3.89	690
Livingstone Shire	RR[13]	0.92	1.19	1.51	29422
Lockhart River Aboriginal Shire	RR[14]	0.56	1.46	3.38	488
Lockyer Valley Regional	RR[15]	0.71	0.94	1.21	31066
Logan City	RR[16]	0.57	0.65	0.73	234853
Longreach Regional	RR[17]	**1.38**	**2.22**	**3.45**	2961
Mackay Regional	RR[18]	**1.03**	**1.20**	**1.39**	91200
Mapoon Aboriginal Shire	RR[19]	0.72	1.68	3.74	225
Maranoa Regional	RR[20]	**1.20**	**1.68**	**2.32**	9908
Mareeba Shire	RR[21]	**1.04**	**1.40**	**1.84**	17681
McKinlay Shire	RR[22]	**1.41**	**2.57**	**4.74**	659
Moreton Bay Regional	RR[23]	0.72	0.80	0.88	336169
Mornington Shire	RR[24]	0.78	1.87	4.02	797
Mount Isa City	RR[25]	**2.40**	**3.04**	**3.80**	14196
Murweh Shire	RR[26]	**1.33**	**2.10**	**3.24**	3404
Napranum Aboriginal Shire	RR[27]	0.79	1.68	3.31	665
Noosa Shire	RR[28]	0.71	0.91	1.15	43701
North Burnett Regional	RR[29]	0.85	1.26	1.82	8621
Northern Peninsula Area Regional	RR[30]	0.50	1.09	2.07	1769
Palm Island Aboriginal Shire	RR[31]	0.38	0.95	1.98	1665
Paroo Shire	RR[32]	0.94	1.70	2.97	1329
Pormpuraaw Aboriginal Shire	RR[33]	**1.14**	**2.33**	**4.73**	548
Quilpie Shire	RR[34]	**1.16**	**2.13**	**3.92**	644

Redland City	RR[35]	0.70	0.83	0.96	119406
Richmond Shire	RR[36]	0.84	1.66	3.04	644
Rockhampton Regional	RR[37]	**1.14**	**1.35**	**1.58**	63150
Scenic Rim Regional	RR[38]	0.69	0.91	1.17	32485
Somerset Regional	RR[39]	0.82	1.12	1.49	19566
South Burnett Regional	RR[40]	0.96	1.24	1.59	26057
Southern Downs Regional	RR[41]	0.91	1.19	1.51	28497
Sunshine Coast Regional	RR[42]	0.73	0.82	0.91	240988
Tablelands Regional	RR[43]	**1.23**	**1.60**	**2.04**	20322
Toowoomba Regional	RR[44]	0.85	0.98	1.12	128187
Torres Shire	RR[45]	0.90	1.61	2.69	2632
Torres Strait Island Regional	RR[46]	0.70	1.38	2.49	2952
Townsville City	RR[47]	0.99	1.12	1.26	149049
Weipa Town	RR[48]	0.63	1.26	2.28	2868
Western Downs Regional	RR[49]	0.91	1.19	1.53	26063
Whitsunday Regional	RR[50]	**1.08**	**1.38**	**1.74**	27657
Winton Shire	RR[51]	**1.73**	**2.92**	**5.12**	950
Woorabinda Aboriginal Shire	RR[52]	0.57	1.23	2.53	638
Wujal Wujal Aboriginal Shire	RR[53]	**1.33**	**2.81**	**6.44**	220
Yarrabah Aboriginal Shire	RR[54]	**2.16**	**3.80**	**6.39**	1692

LGA	SMR	LL	Median	UL	Population
Aurukun Shire	RR[55]	**1.14**	**2.47**	**5.09**	896
Balonne Shire	RR[56]	0.84	1.48	2.53	3433
Banana Shire	RR[57]	**1.14**	**1.68**	**2.40**	11152
Barcaldine Regional	RR[58]	**1.16**	**2.00**	**3.37**	2281
Barcoo Shire	RR[59]	0.94	2.01	4.14	225
Blackall Tambo Regional	RR[60]	**1.05**	**1.95**	**3.48**	1570
Boulia Shire	RR[61]	**1.36**	**2.83**	**5.81**	352
Brisbane City	RR[62]	0.89	0.95	1.01	931878
Bulloo Shire	RR[63]	0.78	1.78	3.93	297
Bundaberg Regional	RR[64]	**1.04**	**1.25**	**1.50**	76491
Burdekin Shire	RR[65]	**1.72**	**2.36**	**3.16**	14040
Burke Shire	RR[66]	**1.14**	**2.42**	**4.95**	276
Cairns Regional	RR[67]	**1.12**	**1.30**	**1.50**	124800
Carpentaria Shire	RR[68]	**1.79**	**3.07**	**5.24**	1553
Cassowary Coast Regional	RR[69]	**1.59**	**2.07**	**2.65**	23460
Central Highlands Regional	RR[70]	0.62	0.92	1.31	21079
Charters Towers Regional	RR[71]	**1.59**	**2.26**	**3.17**	9321
Cherbourg Aboriginal Shire	RR[72]	0.41	1.11	2.78	805
Cloncurry Shire	RR[73]	**1.39**	**2.43**	**4.02**	2418
Cook Shire	RR[74]	**2.66**	**4.18**	**6.43**	3442
Croydon Shire	RR[75]	0.97	2.09	4.35	237
Diamantina Shire	RR[76]	0.95	2.18	4.80	237
Doomadgee Aboriginal Shire	RR[77]	0.53	1.53	3.69	861
Douglas Shire	RR[78]	**1.03**	**1.59**	**2.33**	9781
Etheridge Shire	RR[1]	0.96	1.88	3.54	647
Flinders Shire	RR[2]	**1.20**	**2.23**	**4.03**	1270
Fraser Coast Regional	RR[3]	**1.04**	**1.25**	**1.49**	84462
Gladstone Regional	RR[4]	0.66	0.87	1.13	47696
Gold Coast City	RR[5]	0.74	0.82	0.90	454869
Goondiwindi Regional	RR[6]	0.69	1.13	1.78	8360
Gympie Regional	RR[7]	0.62	0.84	1.11	40504
Hinchinbrook Shire	RR[8]	**1.57**	**2.28**	**3.23**	9178
Hope Vale Aboriginal Shire	RR[9]	0.59	1.64	3.96	658
Ipswich City	RR[10]	0.66	0.78	0.92	147805
Isaac Regional	RR[11]	0.67	1.02	1.48	15636
Kowanyama Aboriginal Shire	RR[12]	**1.12**	**2.45**	**5.14**	690
Livingstone Shire	RR[13]	0.88	1.20	1.58	29422
Lockhart River Aboriginal Shire	RR[14]	0.63	1.76	4.41	488
Lockyer Valley Regional	RR[15]	0.75	1.02	1.37	31066
Logan City	RR[16]	0.54	0.63	0.73	234853
Longreach Regional	RR[17]	**1.48**	**2.50**	**4.08**	2961
Mackay Regional	RR[18]	0.99	1.19	1.41	91200
Mapoon Aboriginal Shire	RR[19]	0.82	2.06	4.91	225
Maranoa Regional	RR[20]	**1.12**	**1.66**	**2.40**	9908
Mareeba Shire	RR[21]	**1.02**	**1.45**	**1.98**	17681
McKinlay Shire	RR[22]	**1.51**	**2.90**	**5.59**	659
Moreton Bay Regional	RR[23]	0.72	0.81	0.90	336169
Mornington Shire	RR[24]	0.88	2.29	5.26	797
Mount Isa City	RR[25]	**2.75**	**3.58**	**4.57**	14196
Murweh Shire	RR[26]	**1.35**	**2.25**	**3.66**	3404
Napranum Aboriginal Shire	RR[27]	0.94	2.09	4.38	665
Noosa Shire	RR[28]	0.72	0.96	1.25	43701
North Burnett Regional	RR[29]	0.78	1.22	1.86	8621
Northern Peninsula Area Regional	RR[30]	0.58	1.34	2.67	1769
Palm Island Aboriginal Shire	RR[31]	0.39	1.05	2.37	1665
Paroo Shire	RR[32]	**1.04**	**1.96**	**3.62**	1329
Pormpuraaw Aboriginal Shire	RR[33]	**1.36**	**2.97**	**6.36**	548
Quilpie Shire	RR[34]	**1.04**	**2.05**	**3.96**	644

Redland City	RR[35]	0.61	0.74	0.90	119406
Richmond Shire	RR[36]	0.91	1.89	3.66	644
Rockhampton Regional	RR[37]	**1.18**	**1.44**	**1.73**	63150
Scenic Rim Regional	RR[38]	0.69	0.95	1.27	32485
Somerset Regional	RR[39]	0.85	1.20	1.66	19566
South Burnett Regional	RR[40]	0.85	1.17	1.57	26057
Southern Downs Regional	RR[41]	0.89	1.21	1.61	28497
Sunshine Coast Regional	RR[42]	0.70	0.80	0.91	240988
Tablelands Regional	RR[43]	**1.20**	**1.63**	**2.16**	20322
Toowoomba Regional	RR[44]	0.82	0.97	1.14	128187
Torres Shire	RR[45]	**1.15**	**2.11**	**3.66**	2632
Torres Strait Island Regional	RR[46]	0.80	1.68	3.17	2952
Townsville City	RR[47]	**1.02**	**1.18**	**1.35**	149049
Weipa Town	RR[48]	0.78	1.64	3.08	2868
Western Downs Regional	RR[49]	0.91	1.24	1.64	26063
Whitsunday Regional	RR[50]	**1.04**	**1.39**	**1.82**	27657
Winton Shire	RR[51]	**1.79**	**3.18**	**5.80**	950
Woorabinda Aboriginal Shire	RR[52]	0.58	1.35	2.96	638
Wujal Wujal Aboriginal Shire	RR[53]	**1.15**	**2.65**	**6.25**	220
Yarrabah Aboriginal Shire	RR[54]	**2.92**	**5.32**	**9.02**	1692

LGA	SMR	LL	Median	UL	Population
Aurukun Shire	RR[55]	**1.23**	**2.76**	**5.83**	896
Balonne Shire	RR[56]	0.79	1.46	2.56	3433
Banana Shire	RR[57]	**1.23**	**1.82**	**2.64**	11152
Barcaldine Regional	RR[58]	**1.11**	**1.97**	**3.42**	2281
Barcoo Shire	RR[59]	0.90	1.99	4.23	225
Blackall Tambo Regional	RR[60]	**1.05**	**1.99**	**3.70**	1570
Boulia Shire	RR[61]	**1.41**	**3.02**	**6.45**	352
Brisbane City	RR[62]	0.89	0.96	1.02	931878
Bulloo Shire	RR[63]	0.74	1.73	3.96	297
Bundaberg Regional	RR[64]	**1.08**	**1.32**	**1.60**	76491
Burdekin Shire	RR[65]	**1.82**	**2.54**	**3.45**	14040
Burke Shire	RR[66]	**1.20**	**2.62**	**5.53**	276
Cairns Regional	RR[67]	**1.11**	**1.31**	**1.52**	124800
Carpentaria Shire	RR[68]	**1.83**	**3.22**	**5.61**	1553
Cassowary Coast Regional	RR[69]	**1.66**	**2.20**	**2.85**	23460
Central Highlands Regional	RR[70]	0.54	0.85	1.25	21079
Charters Towers Regional	RR[71]	**1.59**	**2.33**	**3.33**	9321
Cherbourg Aboriginal Shire	RR[72]	0.40	1.14	3.01	805
Cloncurry Shire	RR[73]	**1.38**	**2.51**	**4.26**	2418
Cook Shire	RR[74]	**2.72**	**4.32**	**6.76**	3442
Croydon Shire	RR[75]	0.99	2.21	4.77	237
Diamantina Shire	RR[76]	0.95	2.24	5.13	237
Doomadgee Aboriginal Shire	RR[77]	0.54	1.65	4.14	861
Douglas Shire	RR[78]	**1.17**	**1.81**	**2.67**	9781
Etheridge Shire	RR[1]	0.99	1.98	3.85	647
Flinders Shire	RR[2]	**1.12**	**2.18**	**4.08**	1270
Fraser Coast Regional	RR[3]	**1.05**	**1.28**	**1.54**	84462
Gladstone Regional	RR[4]	0.63	0.86	1.15	47696
Gold Coast City	RR[5]	0.69	0.77	0.86	454869
Goondiwindi Regional	RR[6]	0.60	1.03	1.68	8360
Gympie Regional	RR[7]	0.57	0.80	1.09	40504
Hinchinbrook Shire	RR[8]	**1.52**	**2.28**	**3.30**	9178
Hope Vale Aboriginal Shire	RR[9]	0.61	1.77	4.40	658
Ipswich City	RR[10]	0.66	0.80	0.95	147805
Isaac Regional	RR[11]	0.63	0.99	1.48	15636
Kowanyama Aboriginal Shire	RR[12]	**1.20**	**2.70**	**5.83**	690
Livingstone Shire	RR[13]	0.83	1.15	1.57	29422
Lockhart River Aboriginal Shire	RR[14]	0.64	1.89	4.92	488
Lockyer Valley Regional	RR[15]	0.76	1.06	1.44	31066
Logan City	RR[16]	0.54	0.64	0.75	234853
Longreach Regional	RR[17]	**1.26**	**2.22**	**3.79**	2961
Mackay Regional	RR[18]	0.96	1.17	1.41	91200
Mapoon Aboriginal Shire	RR[19]	0.86	2.23	5.52	225
Maranoa Regional	RR[20]	**1.12**	**1.71**	**2.53**	9908
Mareeba Shire	RR[21]	**1.16**	**1.65**	**2.28**	17681
McKinlay Shire	RR[22]	**1.60**	**3.16**	**6.27**	659
Moreton Bay Regional	RR[23]	0.68	0.77	0.87	336169
Mornington Shire	RR[24]	0.95	2.54	6.03	797
Mount Isa City	RR[25]	**2.92**	**3.84**	**4.96**	14196
Murweh Shire	RR[26]	**1.23**	**2.14**	**3.62**	3404
Napranum Aboriginal Shire	RR[27]	0.99	2.30	4.97	665
Noosa Shire	RR[28]	0.61	0.85	1.14	43701
North Burnett Regional	RR[29]	0.80	1.28	1.99	8621
Northern Peninsula Area Regional	RR[30]	0.62	1.46	3.00	1769
Palm Island Aboriginal Shire	RR[31]	0.38	1.09	2.55	1665
Paroo Shire	RR[32]	0.93	1.84	3.51	1329
Pormpuraaw Aboriginal Shire	RR[33]	**1.47**	**3.32**	**7.32**	548
Quilpie Shire	RR[34]	0.89	1.85	3.69	644

Redland City	RR[35]	0.57	0.71	0.87	119406
Richmond Shire	RR[36]	0.92	1.99	3.97	644
Rockhampton Regional	RR[37]	**1.11**	**1.37**	**1.69**	63150
Scenic Rim Regional	RR[38]	0.71	1.00	1.36	32485
Somerset Regional	RR[39]	0.88	1.27	1.79	19566
South Burnett Regional	RR[40]	0.81	1.15	1.57	26057
Southern Downs Regional	RR[41]	0.79	1.11	1.52	28497
Sunshine Coast Regional	RR[42]	0.66	0.77	0.89	240988
Tablelands Regional	RR[43]	**1.19**	**1.66**	**2.24**	20322
Toowoomba Regional	RR[44]	0.85	1.02	1.21	128187
Torres Shire	RR[45]	**1.29**	**2.42**	**4.26**	2632
Torres Strait Island Regional	RR[46]	0.91	1.93	3.67	2952
Townsville City	RR[47]	**1.03**	**1.20**	**1.39**	149049
Weipa Town	RR[48]	0.72	1.61	3.16	2868
Western Downs Regional	RR[49]	1.00	1.37	1.84	26063
Whitsunday Regional	RR[50]	**1.05**	**1.43**	**1.91**	27657
Winton Shire	RR[51]	**1.89**	**3.43**	**6.47**	950
Woorabinda Aboriginal Shire	RR[52]	0.56	1.35	3.09	638
Wujal Wujal Aboriginal Shire	RR[53]	**1.24**	**2.95**	**7.26**	220
Yarrabah Aboriginal Shire	RR[54]	**3.07**	**5.74**	**9.98**	1692

LGA	SIR	LL	Median	UL	Population
Abbeywood	RR[1]	0.29	0.91	2.80	20
Abbotsford (Qld)	RR[2]	0.29	0.87	2.46	3
Abercorn	RR[3]	0.27	0.82	2.34	34
Abergowrie	RR[4]	0.44	1.22	2.96	327
Abingdon Downs	RR[5]	0.49	1.35	4.00	0
Abington (Qld)	RR[6]	0.36	1.03	3.01	53
Acacia Ridge	RR[7]	0.81	1.27	1.91	5949
Acland	RR[8]	0.28	0.76	2.20	27
Adare	RR[9]	0.22	0.57	1.33	670
Adavale	RR[10]	0.43	1.16	3.16	80
Adelaide Park	RR[11]	0.35	0.96	2.42	334
Advancetown	RR[12]	0.23	0.62	1.46	380
Aeroglen	RR[13]	0.73	1.88	4.65	314
Agnes Water	RR[14]	0.48	1.08	2.19	1801
Airdmillan	RR[15]	0.33	1.06	3.17	82
Airlie Beach	RR[16]	0.42	0.94	1.89	1150
Airville	RR[17]	0.33	0.90	2.19	283
Aitkenvale	RR[18]	0.60	1.03	1.73	3856
Alabama Hill	RR[19]	0.51	1.54	4.23	99
Albany Creek	RR[20]	0.43	0.64	0.94	12566
Alberta	RR[21]	0.33	0.95	2.69	48
Alberton (Qld)	RR[22]	0.34	0.88	2.07	485
Albinia	RR[23]	0.30	0.91	2.61	109
Albion (Brisbane - Qld)	RR[24]	0.94	1.68	2.89	2054
Albion (Richmond - Qld)	RR[25]	0.49	1.43	4.03	13
Alderley	RR[26]	0.78	1.21	1.84	5008
Aldershot	RR[27]	0.28	0.68	1.65	1049
Aldoga	RR[28]	0.33	0.92	2.95	0
Alexandra (Qld)	RR[29]	0.35	0.92	2.53	168
Alexandra Headland	RR[30]	0.19	0.40	0.85	3541
Alexandra Hills	RR[31]	0.58	0.84	1.18	12995
Algester	RR[32]	0.35	0.62	1.02	6766
Alice Creek	RR[33]	0.30	0.87	2.22	48
Alice River	RR[34]	0.28	0.64	1.31	1811
Allan	RR[35]	0.25	0.75	2.33	73
Allandale (Qld)	RR[36]	0.27	0.74	2.15	44
Allenstown	RR[37]	0.62	1.17	2.05	2313
Allenview	RR[38]	0.22	0.61	1.69	154
Alligator Creek (Mackay - Qld)	RR[39]	0.37	0.95	2.04	617
Alligator Creek (Townsville - Qld)	RR[40]	0.38	0.91	1.97	1064
Allora	RR[41]	0.73	1.57	3.07	1017
Alloway	RR[42]	0.30	0.82	2.10	390
Almaden	RR[43]	0.39	1.15	3.56	58
Aloomba	RR[44]	0.35	0.94	2.29	408
Alpha	RR[45]	0.41	1.08	2.66	276
Alsace	RR[46]	0.34	0.93	2.92	3
Alton Downs	RR[47]	0.32	0.74	1.62	1025
Alva	RR[48]	0.33	0.92	2.52	238
Amamoor	RR[49]	0.37	0.90	2.21	511

Amamoor Creek	RR[50]	0.31	0.99	2.92	43
Amaroo (Qld)	RR[51]	0.42	1.42	4.44	22
Amber	RR[52]	0.45	1.26	3.83	0
Amberley	RR[53]	0.24	0.67	1.68	246
Ambrose	RR[54]	0.29	0.89	2.34	177
Amby	RR[55]	0.32	1.05	3.21	74
Amiens	RR[56]	0.28	0.75	1.99	232
Amity	RR[57]	0.65	2.17	5.31	341
Andergrove	RR[58]	0.62	0.94	1.40	7353
Anderleigh	RR[59]	0.30	0.86	2.33	80
Andromache	RR[60]	0.36	1.00	2.98	48
Anduramba	RR[61]	0.30	0.81	2.46	68
Annandale (Qld)	RR[62]	0.41	0.70	1.13	6750
Annerley	RR[63]	0.79	1.14	1.58	9924
Anstead	RR[64]	0.19	0.47	1.16	1185
Anthony	RR[65]	0.22	0.68	1.94	85
Antigua	RR[66]	0.38	1.08	2.90	120
Apple Tree Creek	RR[67]	0.60	1.43	3.27	525
Applethorpe	RR[68]	0.26	0.73	1.94	422
Arafura Sea	RR[69]	0.19	0.76	2.99	0
Araluen (Qld)	RR[70]	0.23	0.61	1.61	511
Aramac	RR[71]	0.81	2.12	5.63	224
Aramara	RR[72]	0.30	0.86	2.59	54
Arana Hills	RR[73]	0.49	0.83	1.30	5346
Aranbanga	RR[74]	0.30	0.90	2.52	7
Aratula (Qld)	RR[75]	0.20	0.58	1.61	413
Arbouin	RR[76]	0.42	1.31	3.90	0
Arcadia (Qld)	RR[77]	**1.48**	**3.96**	**9.31**	235
Arcadia Valley	RR[78]	0.34	0.94	2.46	81
Archer River	RR[79]	0.41	1.19	3.62	22
Archerfield	RR[80]	0.50	1.29	3.03	471
Arcturus	RR[81]	0.31	0.92	2.61	60
Argoon (Qld)	RR[82]	0.30	0.85	2.35	123
Argyll	RR[83]	0.30	0.95	2.58	26
Armstrong Beach	RR[84]	0.20	0.61	1.58	686
Armstrong Creek (Qld)	RR[85]	0.21	0.58	1.67	270
Aroona	RR[86]	0.19	0.46	0.92	2662
Arriga	RR[87]	0.26	0.68	1.43	1031
Arundel	RR[88]	0.42	0.69	1.06	8464
Ascot (Brisbane - Qld)	RR[89]	0.67	1.12	1.71	4871
Ascot (Toowoomba - Qld)	RR[90]	0.25	0.73	2.12	23
Ashfield (Qld)	RR[91]	0.30	0.81	1.97	594
Ashgrove	RR[92]	0.73	1.02	1.42	10177
Ashmore	RR[93]	0.97	1.35	1.81	9873
Ashwell	RR[94]	0.25	0.71	2.04	64
Aspley	RR[95]	0.70	1.00	1.42	10077
Atherton	RR[96]	**1.01**	**1.49**	**2.14**	6005
Athol	RR[97]	0.23	0.69	1.92	113
Atkinsons Dam	RR[98]	0.60	1.58	4.01	178
Aubigny	RR[99]	0.24	0.69	1.85	203

Auburn (Qld)	RR[100]	0.29	0.91	2.78	23
Auchenflower	RR[101]	0.30	0.59	0.99	5106
Augathella	RR[102]	0.62	1.67	4.04	360
Augustine Heights	RR[103]	0.12	0.28	0.60	3419
Aurukun	RR[104]	0.54	1.25	2.77	896
Austinville	RR[105]	0.23	0.64	1.61	290
Avenell Heights	RR[106]	0.56	1.00	1.61	4081
Avoca (Qld)	RR[107]	0.42	0.76	1.42	4002
Avoca Vale	RR[108]	0.30	0.85	2.46	28
Avondale (Qld)	RR[109]	0.25	0.62	1.57	547
Ayr	RR[110]	**1.45**	**1.99**	**2.65**	7203
Babinda (Qld)	RR[111]	0.95	1.93	3.69	1031
Back Plains	RR[112]	0.27	0.78	2.30	63
Badu Island	RR[113]	0.51	1.41	3.43	526
Baffle Creek	RR[114]	0.28	0.79	2.21	145
Baffle West	RR[115]	0.36	1.12	3.38	10
Bahrs Scrub	RR[116]	0.17	0.41	0.93	1450
Bajool	RR[117]	0.63	1.55	3.84	334
Bakers Bend	RR[118]	0.39	1.16	3.51	23
Bakers Creek (Qld)	RR[119]	0.21	0.61	1.41	925
Baking Board	RR[120]	0.29	0.82	2.36	74
Balberra	RR[121]	0.34	0.88	2.14	366
Balcomba	RR[122]	0.36	0.98	2.65	9
Bald Hills (Qld)	RR[123]	0.42	0.74	1.21	5165
Bald Knob	RR[124]	0.28	0.79	2.16	231
Balgal Beach	RR[125]	0.44	1.21	2.80	816
Balgowan (Qld)	RR[126]	0.24	0.75	2.40	11
Ball Bay	RR[127]	0.25	0.71	2.06	319
Ballandean	RR[128]	0.38	1.06	2.80	281
Ballard	RR[129]	0.41	1.10	2.92	125
Ballaroo	RR[130]	0.32	0.94	2.64	34
Ballogie	RR[131]	0.49	1.25	3.09	246
Balmoral (Qld)	RR[132]	0.70	1.23	2.06	3127
Balmoral Ridge	RR[133]	0.24	0.64	1.66	246
Balnagowan	RR[134]	0.24	0.66	1.85	293
Bamaga	RR[135]	0.23	0.71	1.87	767
Bambaroo	RR[136]	0.34	1.03	2.93	108
Bamboo	RR[137]	0.42	1.25	3.67	104
Bamboo Creek	RR[138]	0.45	1.24	3.28	75
Ban Ban	RR[139]	0.30	0.85	2.61	20
Ban Ban Springs	RR[140]	0.21	0.88	3.68	4
Banana	RR[141]	0.29	0.81	2.18	275
Bancroft	RR[142]	0.26	0.79	2.28	74
Bangalee (Qld)	RR[143]	0.19	0.77	2.65	148
Bangall	RR[144]	0.47	1.46	4.15	4
Banks Creek	RR[145]	0.26	0.78	2.39	5
Banks Pocket	RR[146]	0.23	0.71	1.95	126
Banksia Beach	RR[147]	0.42	0.75	1.26	5141
Bannockburn (Qld)	RR[148]	0.24	0.64	1.60	591
Banyo	RR[149]	1.00	1.49	2.23	4780

Bapaume	RR[150]	0.26	0.82	2.36	94
Barakula	RR[151]	0.30	0.90	2.72	10
Baralaba	RR[152]	0.49	1.26	3.39	283
Barambah	RR[153]	0.28	0.84	2.28	36
Barcaldine	RR[154]	0.51	1.12	2.27	1129
Barcaldine Downs	RR[155]	0.45	1.26	3.79	14
Bardon	RR[156]	0.49	0.80	1.24	7370
Baree	RR[157]	0.49	1.37	3.97	143
Barellan Point	RR[158]	0.38	0.92	2.04	939
Bargara	RR[159]	0.78	1.22	1.81	6328
Bargunyah	RR[160]	0.32	1.01	3.03	22
Barker Creek Flat	RR[161]	0.30	0.91	2.54	43
Barkly (Qld)	RR[162]	0.53	1.59	4.57	28
Barlil	RR[163]	0.27	0.82	2.20	30
Barlows Hill	RR[164]	0.51	1.25	2.77	649
Barlyne	RR[165]	0.30	0.91	2.89	3
Barmaryee	RR[166]	0.33	0.86	1.92	672
Barmoya	RR[167]	0.44	1.26	3.51	57
Barnard	RR[168]	0.30	0.95	2.57	25
Barney Point	RR[169]	0.41	1.01	2.19	927
Barney View	RR[170]	0.24	0.78	2.24	53
Baroondah	RR[171]	0.34	0.99	2.85	1
Barramornie	RR[172]	0.29	0.95	2.72	15
Barratta (Qld)	RR[173]	0.37	1.05	2.84	54
Barrine	RR[174]	0.43	1.16	3.17	211
Barringha	RR[175]	0.33	1.01	2.99	50
Barringun	RR[176]	0.33	1.15	3.80	7
Barron	RR[177]	0.41	1.14	3.36	36
Barron Gorge	RR[178]	0.42	1.09	3.21	0
Bartle Frere	RR[179]	0.39	1.09	3.02	115
Barwidgi	RR[180]	0.41	1.23	3.72	0
Basalt (Qld)	RR[181]	0.40	1.06	2.81	160
Basilisk	RR[182]	0.41	1.27	3.71	0
Basin Pocket	RR[183]	0.45	1.02	2.26	730
Battery Hill	RR[184]	0.56	1.08	2.06	2083
Bauhinia	RR[185]	0.33	0.95	2.77	36
Bauple	RR[186]	0.62	1.51	3.46	567
Bauple Forest	RR[187]	0.34	0.93	2.67	7
Bayrick	RR[188]	0.37	1.18	3.41	9
Bayview Heights	RR[189]	0.52	0.94	1.56	3384
Beach Holm	RR[190]	0.28	0.80	2.39	32
Beachmere	RR[191]	0.58	1.06	1.77	3455
Beaconsfield (Qld)	RR[192]	0.48	0.85	1.38	4303
Beatrice	RR[193]	0.37	1.09	3.02	79
Beaudesert	RR[194]	0.88	1.34	2.05	5132
Beaufort (Qld)	RR[195]	0.34	1.00	2.82	39
Beaver Rock	RR[196]	0.29	0.90	2.81	46
Bedourie	RR[197]	0.43	1.33	3.76	88
Beebo	RR[198]	0.29	0.92	2.81	67
Beecher	RR[199]	0.41	1.04	2.45	670

Beechmont	RR[200]	0.27	0.67	1.48	704
Beelbee	RR[201]	0.26	0.81	2.61	14
Beelbi Creek	RR[202]	0.40	1.22	3.29	111
Beenaam Valley	RR[203]	0.26	0.78	2.40	46
Beenleigh	RR[204]	0.92	1.38	1.96	6670
Beerburrum	RR[205]	0.33	0.89	2.13	622
Beeron	RR[206]	0.31	0.89	2.62	11
Beerwah	RR[207]	0.41	0.71	1.18	5382
Begonia	RR[208]	0.30	0.96	2.89	33
Beilba	RR[209]	0.37	1.06	2.85	46
Belcong	RR[210]	0.28	0.79	2.37	35
Belgian Gardens	RR[211]	0.50	1.05	1.93	1693
Belivah	RR[212]	0.21	0.58	1.61	273
Bell (Qld)	RR[213]	0.38	1.02	2.56	405
Bella Creek	RR[214]	0.35	1.12	3.55	40
Bellara	RR[215]	**2.07**	**3.15**	**4.58**	2869
Bellbird Park	RR[216]	0.58	0.98	1.52	5106
Bellbowrie	RR[217]	0.25	0.51	0.99	4327
Bellenden Ker	RR[218]	0.36	1.06	2.82	204
Bellevue (Qld)	RR[219]	0.43	1.30	3.66	0
Bellfield (Qld)	RR[220]	0.45	1.42	4.66	0
Belli Park	RR[221]	0.23	0.63	1.55	555
Bellmere	RR[222]	0.36	0.68	1.19	4386
Bells Bridge	RR[223]	0.26	0.72	2.01	166
Bells Creek	RR[224]	0.26	0.70	1.97	85
Bellthorpe	RR[225]	0.27	0.72	1.88	115
Belmont (Qld)	RR[226]	0.35	0.66	1.19	3482
Belmunda	RR[227]	0.22	0.88	3.15	7
Belvedere	RR[228]	0.57	1.42	2.95	699
Belyando	RR[229]	0.37	0.99	2.71	50
Bemerside	RR[230]	0.58	1.52	4.07	190
Benair	RR[231]	0.29	0.83	2.38	116
Benaraby	RR[232]	0.47	1.05	2.26	911
Benarkin	RR[233]	0.27	0.81	2.54	51
Benarkin North	RR[234]	0.34	0.92	2.40	273
Benholme	RR[235]	0.27	0.81	2.43	76
Benobble	RR[236]	0.26	0.75	2.26	32
Benowa	RR[237]	0.76	1.18	1.73	7088
Bentley Park	RR[238]	0.32	0.59	0.94	5788
Berajondo	RR[239]	0.26	0.82	2.37	54
Berat	RR[240]	0.24	0.76	2.18	87
Bergen	RR[241]	0.29	0.79	2.13	54
Berrinba	RR[242]	0.18	0.46	1.06	982
Berserker	RR[243]	0.78	1.21	1.83	5554
Bethania	RR[244]	0.64	1.06	1.70	4543
Biarra	RR[245]	0.28	0.78	2.10	199
Biboohra	RR[246]	0.42	1.06	2.58	469
Biddaddaba	RR[247]	0.35	0.94	2.57	152
Biddeston	RR[248]	0.23	0.66	1.75	228
Bidwill (Qld)	RR[249]	0.27	0.71	1.96	396

Biggenden	RR[250]	0.84	1.81	3.67	714
Biggera Waters	RR[251]	0.63	0.97	1.44	7397
Bilinga	RR[252]	0.25	0.64	1.45	1615
Billa Billa	RR[253]	0.29	0.84	2.76	74
Biloela	RR[254]	0.57	0.99	1.61	4410
Bilyana	RR[255]	0.32	0.99	2.91	164
Bindebango	RR[256]	0.37	1.05	2.70	10
Bingegang	RR[257]	0.31	0.90	2.96	18
Bingil Bay	RR[258]	0.54	1.44	3.52	347
Binjour	RR[259]	0.51	1.47	4.21	77
Binna Burra (Qld)	RR[260]	0.24	0.73	2.33	4
Birdsville	RR[261]	0.50	1.48	4.06	121
Birkalla	RR[262]	0.40	1.08	3.36	108
Birkdale	RR[263]	0.56	0.83	1.16	11769
Birnam (Scenic Rim - Qld)	RR[264]	0.24	0.67	1.98	88
Birnam (Toowoomba - Qld)	RR[265]	0.21	0.68	1.98	53
Birtinya	RR[266]	0.34	0.73	1.52	1578
Black Duck Creek	RR[267]	0.22	0.69	2.17	18
Black Jack	RR[268]	0.37	1.06	2.99	125
Black Mountain (Qld)	RR[269]	0.39	0.88	1.78	1213
Black River (Qld)	RR[270]	0.31	0.76	1.66	1192
Black Snake	RR[271]	0.28	0.82	2.32	65
Blackall	RR[272]	0.77	1.57	3.07	1176
Blackbull	RR[273]	0.49	1.54	4.84	3
Blackbutt (Qld)	RR[274]	0.52	1.27	2.86	713
Blackbutt North	RR[275]	0.25	0.71	2.09	351
Blackbutt South	RR[276]	0.25	0.80	2.33	146
Blackdown	RR[277]	0.29	0.96	3.01	0
Blackrock	RR[278]	0.69	1.74	4.15	264
Blacks Beach	RR[279]	0.18	0.41	0.83	2826
Blacksoil	RR[280]	0.34	1.01	2.87	98
Blackstone	RR[281]	0.56	1.21	2.77	837
Blackswamp	RR[282]	0.30	0.86	2.64	47
Blackwater	RR[283]	0.53	0.94	1.58	3536
Blairmore	RR[284]	0.28	0.90	2.89	0
Blanchview	RR[285]	0.23	0.67	1.75	158
Blantyre	RR[286]	0.20	0.66	2.01	49
Blaxland (Qld)	RR[287]	0.24	0.76	2.41	24
Blenheim	RR[288]	0.28	0.77	2.04	240
Bli Bli	RR[289]	0.36	0.63	1.00	6255
Bloomfield	RR[290]	0.58	1.60	4.17	178
Bloomsbury	RR[291]	0.53	1.39	3.20	477
Blue Mountain	RR[292]	0.27	0.80	2.21	72
Blue Mountain Heights	RR[293]	0.28	0.78	1.82	753
Bluewater	RR[294]	0.36	0.90	2.16	826
Bluewater Park	RR[295]	0.30	0.80	1.83	751
Bluff	RR[296]	0.48	1.20	2.92	317
Blythdale	RR[297]	0.33	1.00	2.84	33
Boatman	RR[298]	0.37	1.09	2.98	6
Bogandilla	RR[299]	0.30	0.95	2.90	24

Bogie	RR[300]	0.36	0.99	2.40	141
Bohle	RR[301]	0.60	1.81	4.92	76
Bohle Plains	RR[302]	0.22	0.48	1.04	2288
Boigu Island	RR[303]	0.32	1.18	3.63	174
Bokarina	RR[304]	0.28	0.77	1.70	1038
Bollier	RR[305]	0.28	0.75	2.01	172
Bollon	RR[306]	0.32	0.89	2.54	168
Bolwarra (Qld)	RR[307]	0.44	1.32	4.19	0
Bombeeta	RR[308]	0.37	1.08	3.11	112
Bon Accord	RR[309]	0.31	0.92	2.56	15
Bondoola	RR[310]	0.30	0.78	1.86	432
Bongaree	RR[311]	**1.80**	**2.46**	**3.29**	6400
Bongeen	RR[312]	0.26	0.76	2.11	50
Bonnie Doon (Qld)	RR[313]	0.51	1.33	3.18	268
Bonogin	RR[314]	0.26	0.53	1.00	3510
Bonshaw (Qld)	RR[315]	0.28	0.92	3.13	28
Bony Mountain	RR[316]	0.31	0.81	2.21	78
Boodua	RR[317]	0.24	0.72	2.07	75
Boogan	RR[318]	0.56	1.58	4.03	91
Booie	RR[319]	0.31	0.75	1.80	814
Boolboonda	RR[320]	0.26	0.81	2.75	52
Boolburra	RR[321]	0.29	0.99	3.06	24
Boompa	RR[322]	0.29	0.88	2.44	68
Boonah (Qld)	RR[323]	**1.39**	**2.37**	**3.73**	2058
Boonara	RR[324]	0.30	0.82	2.47	54
Boonarga	RR[325]	0.28	0.84	2.38	28
Boondall	RR[326]	**1.02**	**1.46**	**2.01**	7576
Boondandilla	RR[327]	0.29	0.91	2.60	0
Boondooma	RR[328]	0.33	0.86	2.34	63
Boonooroo	RR[329]	0.25	0.77	2.10	303
Boonooroo Plains	RR[330]	0.40	1.21	3.80	3
Booral (Qld)	RR[331]	0.37	0.88	1.83	1254
Booroobin	RR[332]	0.22	0.68	1.76	220
Booubyjan	RR[333]	0.33	0.85	2.16	82
Booval	RR[334]	**1.56**	**2.53**	**3.88**	2214
Booyal	RR[335]	0.32	0.85	2.32	210
Borallon	RR[336]	0.47	1.26	3.64	64
Boreen Point	RR[337]	0.32	0.88	2.40	300
Boronia Heights	RR[338]	0.47	0.76	1.24	5968
Bororen	RR[339]	0.26	0.71	1.78	324
Boulder Creek	RR[340]	0.34	1.11	3.11	12
Bouldercombe	RR[341]	0.46	1.03	2.15	866
Boulia	RR[342]	0.65	1.96	5.57	249
Bountiful Islands	RR[343]	0.21	1.23	7.36	0
Bowen	RR[344]	**1.24**	**1.72**	**2.28**	8442
Bowen Hills	RR[345]	0.27	0.57	1.14	3108
Bowenville	RR[346]	0.34	0.89	2.51	201
Boyland	RR[347]	0.28	0.74	1.78	627
Boyne Island	RR[348]	0.32	0.63	1.19	3588
Boyne Valley	RR[349]	0.25	0.71	1.88	281

Boynedale	RR[350]	0.24	0.78	2.30	17
Boyneside	RR[351]	0.31	0.84	2.50	46
Boynewood	RR[352]	0.28	0.86	2.50	150
Bracalba	RR[353]	0.25	0.74	2.04	94
Bracewell	RR[354]	0.32	0.83	2.39	151
Bracken Ridge	RR[355]	0.65	0.93	1.27	13493
Braemeadows	RR[356]	0.40	1.18	3.05	250
Braemore	RR[357]	0.28	0.80	2.19	117
Bramston Beach	RR[358]	0.30	1.01	3.13	158
Branch Creek	RR[359]	0.31	0.87	2.79	32
Branchview	RR[360]	0.26	0.75	2.29	14
Brandon	RR[361]	0.26	0.69	1.64	892
Brandy Creek (Qld)	RR[362]	0.26	0.82	2.71	96
Branyan	RR[363]	0.75	1.31	2.15	3191
Brassall	RR[364]	0.69	1.06	1.55	8381
Bray Park (Qld)	RR[365]	0.30	0.53	0.86	7991
Breadalbane (Qld)	RR[366]	0.32	0.99	2.92	20
Breakaway	RR[367]	0.43	1.31	3.85	165
Breddan	RR[368]	0.33	0.87	2.19	378
Brendale	RR[369]	0.24	0.53	1.07	2177
Bribie Island North	RR[370]	0.23	1.34	6.22	0
Bridgeman Downs	RR[371]	0.19	0.37	0.66	6837
Bridges	RR[372]	0.31	0.90	2.38	234
Brigalow	RR[373]	0.30	0.79	2.23	138
Brightly	RR[374]	0.27	0.78	2.03	59
Brighton (Qld)	RR[375]	0.79	1.19	1.70	7594
Brightview	RR[376]	0.23	0.59	1.38	653
Brigooda	RR[377]	0.29	0.93	2.79	27
Bringalily	RR[378]	0.28	0.83	2.44	60
Brinsmead	RR[379]	0.49	0.88	1.46	4157
Brisbane Airport	RR[380]	0.41	1.20	3.66	0
Brisbane City	RR[381]	0.31	0.53	0.86	9012
Broadbeach	RR[382]	0.63	1.07	1.64	5093
Broadbeach Waters	RR[383]	0.71	1.10	1.60	6509
Broadmere	RR[384]	0.32	0.97	2.90	41
Broadwater (Qld)	RR[385]	0.29	0.81	2.05	223
Broken River	RR[386]	0.29	0.87	2.72	19
Bromelton	RR[387]	0.25	0.68	1.83	126
Brookfield (Qld)	RR[388]	0.34	0.69	1.29	2876
Brookhill	RR[389]	0.49	1.30	3.56	64
Brooklands	RR[390]	0.38	0.97	2.45	244
Brookstead	RR[391]	0.26	0.74	1.84	165
Brookwater	RR[392]	0.22	0.51	1.07	1625
Brooloo	RR[393]	0.32	0.92	2.31	293
Brooweena	RR[394]	0.31	0.89	2.44	88
Broughton (Qld)	RR[395]	0.31	0.81	1.88	551
Brovinia	RR[396]	0.32	0.91	2.67	7
Browns Plains (Qld)	RR[397]	0.19	0.40	0.76	4905
Broxburn	RR[398]	0.25	0.72	2.05	123
Brush Creek	RR[399]	0.32	0.94	2.84	37

Bryden	RR[400]	0.28	0.76	2.28	33
Brymaroo	RR[401]	0.28	0.74	2.10	103
Buaraba	RR[402]	0.27	0.77	2.04	156
Buaraba South	RR[403]	0.25	0.78	2.20	0
Bucasia	RR[404]	0.37	0.72	1.25	3495
Bucca (Qld)	RR[405]	0.29	0.72	1.65	865
Buccan	RR[406]	0.28	0.69	1.46	1481
Buckingham (Qld)	RR[407]	0.48	1.52	4.58	0
Buckland (Qld)	RR[408]	0.38	1.02	3.03	37
Buddina	RR[409]	0.58	1.07	1.85	3158
Buderim	RR[410]	0.65	0.85	1.09	24204
Budgee	RR[411]	0.22	0.69	1.91	32
Bukali	RR[412]	0.26	0.81	2.58	51
Bulgun	RR[413]	0.38	1.04	2.80	226
Bulimba	RR[414]	0.80	1.26	1.90	5586
Bullawarra	RR[415]	0.39	1.14	3.84	5
Bullcamp	RR[416]	0.28	0.85	2.52	49
Bulleringa	RR[417]	0.43	1.26	4.25	0
Bulli Creek	RR[418]	0.30	0.87	2.53	6
Bulloo Downs	RR[419]	0.37	1.18	3.40	0
Bullyard	RR[420]	0.44	1.25	3.16	158
Bulwer	RR[421]	0.00	0.00	0.00	49
Bunburra	RR[422]	0.23	0.68	1.98	86
Bundaberg Central	RR[423]	**5.84**	**11.07**	**19.47**	294
Bundaberg East	RR[424]	0.55	1.09	2.02	2223
Bundaberg North	RR[425]	0.68	1.15	1.80	4602
Bundaberg South	RR[426]	0.54	1.05	1.84	2774
Bundaberg West	RR[427]	0.87	1.59	2.72	2117
Bundall	RR[428]	0.46	0.82	1.48	3716
Bundamba	RR[429]	0.62	1.03	1.60	5247
Bundi	RR[430]	0.33	0.96	2.73	35
Bundoora (Qld)	RR[431]	0.26	0.80	2.52	9
Bungaban	RR[432]	0.33	0.91	2.60	40
Bungadoo	RR[433]	0.28	0.77	1.97	280
Bungalow	RR[434]	0.68	1.31	2.35	1908
Bungeworgorai	RR[435]	0.36	0.99	2.77	52
Bungil (Qld)	RR[436]	0.35	0.98	3.01	24
Bungundarra	RR[437]	0.34	0.91	2.30	437
Bungunya	RR[438]	0.29	0.88	2.60	49
Bunjurgen	RR[439]	0.26	0.68	2.00	99
Bunya	RR[440]	0.22	0.53	1.12	1525
Bunya Creek	RR[441]	0.29	0.78	2.31	62
Bunya Mountains	RR[442]	0.28	0.78	2.19	123
Burbank	RR[443]	0.20	0.50	1.16	855
Burdell	RR[444]	0.26	0.50	0.94	4235
Burgowan	RR[445]	0.33	0.94	2.78	29
Burketown	RR[446]	0.51	1.63	4.55	196
Burleigh	RR[447]	0.45	1.37	4.44	44
Burleigh Heads	RR[448]	0.94	1.34	1.88	8672
Burleigh Waters	RR[449]	0.94	1.28	1.69	11555

Burncluith	RR[450]	0.28	0.85	2.37	122
Burnett Creek	RR[451]	0.23	0.71	2.00	11
Burnett Heads	RR[452]	0.63	1.24	2.22	2256
Burnside (Qld)	RR[453]	0.30	0.71	1.43	1857
Burpengary	RR[454]	0.43	0.67	1.00	11135
Burpengary East	RR[455]	0.17	0.36	0.68	5371
Burra Burri	RR[456]	0.31	0.89	2.64	36
Burrar Islet	RR[457]	0.31	1.35	5.08	0
Burrum Heads	RR[458]	0.39	0.86	1.72	1884
Burrum River	RR[459]	0.33	0.88	2.37	215
Burrum Town	RR[460]	0.33	0.93	2.69	147
Burton (Qld)	RR[461]	0.30	0.85	2.43	29
Burua	RR[462]	0.22	0.55	1.37	583
Bushland Beach	RR[463]	0.17	0.38	0.72	4536
Bushley	RR[464]	0.44	1.43	4.28	27
Butchers Creek	RR[465]	0.39	1.08	3.12	86
Buxton (Qld)	RR[466]	0.40	1.02	2.44	409
Bybera	RR[467]	0.29	0.92	2.86	3
Byee	RR[468]	0.25	0.87	2.51	21
Byellee	RR[469]	0.26	0.78	2.30	0
Byfield	RR[470]	0.36	1.03	3.10	238
Bymount	RR[471]	0.35	1.05	3.22	29
Byrnestown	RR[472]	0.41	1.31	3.96	27
Cabarlah	RR[473]	0.18	0.50	1.13	830
Caboolture	RR[474]	0.64	0.85	1.12	20780
Caboolture South	RR[475]	0.49	0.83	1.39	4182
Caboonbah	RR[476]	0.28	0.86	2.71	14
Cadarga	RR[477]	0.29	0.91	2.99	13
Caffey	RR[478]	0.23	0.66	2.01	55
Cainbable	RR[479]	0.23	0.73	2.34	64
Cairdbeign	RR[480]	0.30	0.93	2.61	66
Cairns City	RR[481]	**2.82**	**3.96**	**5.57**	2588
Cairns North	RR[482]	0.37	0.68	1.21	4594
Calamvale	RR[483]	0.25	0.42	0.66	13620
Calavos	RR[484]	0.30	0.93	2.26	279
Calcium	RR[485]	0.36	1.05	3.08	18
Caldervale	RR[486]	0.41	1.10	3.08	8
Calen	RR[487]	0.62	1.50	3.83	310
Calgoa	RR[488]	0.31	0.89	2.73	9
Calico Creek	RR[489]	0.24	0.72	1.99	128
Calingunee	RR[490]	0.29	0.90	2.85	19
Callandoon	RR[491]	0.28	0.91	2.79	25
Callemondah	RR[492]	0.28	0.84	2.46	18
Callide	RR[493]	0.27	0.84	2.57	66
Calliope (Qld)	RR[494]	0.27	0.54	0.99	3644
Caloundra	RR[495]	**1.96**	**2.83**	**4.00**	3568
Caloundra West	RR[496]	0.24	0.48	0.85	4796
Calvert (Qld)	RR[497]	0.27	0.82	2.00	260
Camboon (Qld)	RR[498]	0.31	0.90	2.41	69
Cambooya	RR[499]	0.29	0.68	1.51	1189

Cambridge (Qld)	RR[500]	0.45	1.41	4.22	34
Cambroon	RR[501]	0.45	1.30	3.44	180
Cameby	RR[502]	0.27	0.86	2.42	47
Cameron Corner	RR[503]	0.35	1.21	4.15	5
Camira (Qld)	RR[504]	0.50	0.85	1.32	5938
Camoola	RR[505]	0.51	1.39	3.71	23
Camooweal	RR[506]	0.61	1.79	4.41	162
Camp Creek (Qld)	RR[507]	0.39	1.12	2.94	131
Camp Hill	RR[508]	0.74	1.08	1.55	8574
Camp Mountain	RR[509]	0.41	0.96	2.03	1112
Campaspe	RR[510]	0.32	1.04	2.83	93
Campbell Creek	RR[511]	0.32	0.90	2.71	7
Campbells Pocket	RR[512]	0.23	0.71	1.89	66
Campwin Beach	RR[513]	0.22	0.71	1.87	414
Canaga	RR[514]	0.28	0.82	2.18	81
Canal Creek	RR[515]	0.31	0.94	2.87	8
Cania	RR[516]	0.30	0.82	2.25	24
Canina	RR[517]	0.21	0.65	1.77	303
Cannindah	RR[518]	0.27	0.82	2.53	29
Canning Creek	RR[519]	0.29	0.88	2.55	5
Canningvale	RR[520]	0.24	0.71	1.92	124
Cannon Creek (Scenic Rim - Qld)	RR[521]	0.24	0.70	2.14	69
Cannon Creek (Southern Downs - Q	RR[522]	0.30	0.87	2.37	27
Cannon Hill	RR[523]	0.94	1.54	2.28	4470
Cannon Valley	RR[524]	0.35	0.87	2.02	735
Cannonvale	RR[525]	0.39	0.71	1.19	4495
Canoona	RR[526]	0.35	0.94	2.52	68
Canungra	RR[527]	0.51	1.24	2.62	978
Capalaba	RR[528]	0.61	0.87	1.19	14072
Cape Cleveland	RR[529]	0.65	1.85	4.71	122
Cape Conway	RR[530]	0.30	0.83	2.55	0
Cape Gloucester	RR[531]	0.24	0.83	2.81	56
Cape Hillsborough	RR[532]	0.20	0.79	3.56	37
Cape Tribulation	RR[533]	0.45	1.31	3.87	104
Capella	RR[534]	0.21	0.57	1.42	748
Captain Creek	RR[535]	0.25	0.74	2.00	282
Captains Mountain	RR[536]	0.28	0.79	2.27	69
Caravonica	RR[537]	0.49	1.02	2.07	1588
Carbine Creek	RR[538]	0.29	0.91	2.87	20
Carbrook	RR[539]	0.28	0.71	1.61	1030
Cardstone	RR[540]	0.38	1.16	3.61	17
Cardwell	RR[541]	**1.16**	**2.26**	**4.05**	1169
Carina (Qld)	RR[542]	0.60	0.91	1.33	9139
Carina Heights	RR[543]	0.32	0.55	0.97	5661
Carindale	RR[544]	0.39	0.60	0.85	13060
Carmila	RR[545]	0.26	0.79	2.13	286
Carmoo	RR[546]	0.55	1.65	4.43	159
Carnarvon Park	RR[547]	0.38	1.06	2.97	3
Carneys Creek	RR[548]	0.24	0.69	1.98	38
Carole Park	RR[549]	0.34	1.10	3.10	4

Caroline Crossing	RR[550]	0.38	1.16	3.53	5
Carpendale	RR[551]	0.21	0.67	2.07	105
Carpentaria	RR[552]	0.53	1.67	4.79	9
Carrandotta	RR[553]	0.52	1.48	4.71	0
Carrara	RR[554]	0.35	0.57	0.88	10011
Carrington (Qld)	RR[555]	0.46	1.29	3.45	153
Carruchan	RR[556]	0.36	1.03	2.69	217
Carseldine	RR[557]	0.43	0.70	1.08	7949
Carstairs	RR[558]	0.38	1.06	3.00	97
Carters Ridge	RR[559]	0.23	0.63	1.60	386
Cashmere	RR[560]	0.20	0.45	0.91	3779
Cassowary	RR[561]	0.37	1.07	2.99	98
Castaways Beach	RR[562]	0.24	0.61	1.62	546
Castle Creek (Qld)	RR[563]	0.31	0.93	2.60	19
Castle Hill (Qld)	RR[564]	0.48	1.16	2.42	781
Cattle Creek (North Burnett - Qld)	RR[565]	0.29	0.91	2.70	25
Cattle Creek (Toowoomba - Qld)	RR[566]	0.28	0.79	2.47	19
Causeway Lake	RR[567]	0.71	2.00	5.86	110
Cawarral	RR[568]	0.38	0.90	2.04	653
Cawdor (Qld)	RR[569]	0.21	0.62	1.75	234
Cecil Plains	RR[570]	0.24	0.66	1.74	362
Cedar Creek (Gold Coast - Qld)	RR[571]	0.49	1.13	2.36	650
Cedar Creek (Moreton Bay - Qld)	RR[572]	0.21	0.55	1.33	553
Cedar Grove	RR[573]	0.14	0.38	0.89	1633
Cedar Pocket	RR[574]	0.28	0.83	2.35	286
Cedar Vale	RR[575]	0.38	0.78	1.50	2081
Cedarton	RR[576]	0.23	0.70	2.18	104
Cement Mills	RR[577]	0.28	0.86	2.77	13
Centenary Heights	RR[578]	0.42	0.77	1.25	4946
Ceratodus	RR[579]	0.27	0.88	2.68	22
Chahpingah	RR[580]	0.31	0.84	2.50	26
Chambers Flat	RR[581]	0.59	1.13	2.05	1924
Chances Plain	RR[582]	0.29	0.84	2.28	116
Chandler (Qld)	RR[583]	0.29	0.68	1.62	1162
Chapel Hill (Qld)	RR[584]	0.27	0.47	0.76	8152
Charlestown (Qld)	RR[585]	0.30	0.82	2.31	58
Charleville	RR[586]	**1.07**	**1.78**	**2.84**	2615
Charlton (Qld)	RR[587]	0.23	0.68	1.94	104
Charlwood	RR[588]	0.25	0.66	1.76	123
Charters Towers City	RR[589]	**2.68**	**4.14**	**6.18**	1706
Chatsworth (Qld)	RR[590]	0.27	0.66	1.54	852
Cheeseborough	RR[591]	0.30	0.94	2.76	3
Chelmer	RR[592]	**1.13**	**2.03**	**3.32**	2303
Chelmsford	RR[593]	0.29	0.83	2.32	97
Chelona	RR[594]	0.26	0.83	2.38	80
Cheltenham (Qld)	RR[595]	0.31	0.92	2.81	17
Cherbourg	RR[596]	0.29	0.75	1.62	805
Chermside	RR[597]	0.52	0.83	1.27	8104
Chermside West	RR[598]	0.33	0.61	1.05	5329
Cherry Creek	RR[599]	0.28	0.86	2.44	44

Cherry Gully	RR[600]	0.26	0.80	2.66	9
Cherwell	RR[601]	0.36	1.05	2.95	5
Chevallum	RR[602]	0.28	0.76	1.95	355
Chewko	RR[603]	0.44	1.29	3.32	165
Childers (Qld)	RR[604]	**1.71**	**2.91**	**4.77**	1354
Chillagoe	RR[605]	0.37	1.02	2.65	207
Chinchilla	RR[606]	0.52	0.89	1.40	5014
Chinghee Creek	RR[607]	0.23	0.76	2.45	20
Chirnside	RR[608]	0.25	0.83	2.29	22
Chorregon	RR[609]	0.47	1.45	3.94	52
Christmas Creek	RR[610]	0.25	0.74	2.23	40
Churchable	RR[611]	0.43	1.19	3.13	225
Churchill (Qld)	RR[612]	0.39	0.86	1.70	1449
Chuwar	RR[613]	0.24	0.55	1.22	1731
Cinnabar	RR[614]	0.28	0.83	2.33	56
Clagiraba	RR[615]	0.31	0.75	1.73	479
Clairview	RR[616]	0.27	0.89	2.80	134
Clara Creek	RR[617]	0.39	1.11	3.12	33
Claraville	RR[618]	0.54	1.49	4.37	5
Clare (Qld)	RR[619]	0.44	1.29	3.38	163
Clarendon (Qld)	RR[620]	0.26	0.74	2.07	191
Clarke Creek	RR[621]	0.31	0.92	2.67	26
Clayfield	RR[622]	**1.17**	**1.59**	**2.12**	8910
Clear Island Waters	RR[623]	0.39	0.77	1.34	3554
Clear Mountain	RR[624]	0.29	0.78	1.94	605
Clemant	RR[625]	0.32	1.00	2.94	0
Clermont	RR[626]	0.99	1.71	2.76	2328
Cleveland (Qld)	RR[627]	0.78	1.07	1.43	12700
Clifford	RR[628]	0.34	0.92	2.65	22
Clifton (Qld)	RR[629]	0.53	1.16	2.17	1189
Clifton Beach (Qld)	RR[630]	0.21	0.48	1.01	2571
Clinton (Qld)	RR[631]	0.35	0.64	1.17	4436
Clintonvale	RR[632]	0.28	0.74	2.07	60
Cloncurry	RR[633]	0.99	1.68	2.75	2169
Clontarf (Moreton Bay - Qld)	RR[634]	0.83	1.24	1.83	6870
Clontarf (Toowoomba - Qld)	RR[635]	0.29	0.84	2.58	18
Closeburn	RR[636]	0.24	0.70	1.67	465
Cloyna	RR[637]	0.25	0.78	2.37	105
Cluden	RR[638]	0.34	0.85	2.23	359
Clumber	RR[639]	0.24	0.70	2.01	56
Coal Creek	RR[640]	0.29	0.86	2.58	47
Coalbank	RR[641]	0.29	0.82	2.44	24
Coalfalls	RR[642]	0.59	1.39	2.95	724
Coalstoun Lakes	RR[643]	0.32	0.93	2.52	92
Cobbs Hill	RR[644]	0.30	0.88	2.47	16
Cobraball	RR[645]	0.31	1.00	2.94	56
Cockatoo (Qld)	RR[646]	0.31	0.95	2.77	29
Coconuts	RR[647]	0.35	1.10	2.93	186
Coen	RR[648]	0.48	1.30	3.28	273
Coes Creek	RR[649]	0.19	0.51	1.19	1121

Coles Creek	RR[650]	0.25	0.74	2.09	40
Colevale	RR[651]	0.34	1.09	3.43	17
Coleyville	RR[652]	0.20	0.61	1.69	155
Colinton (Qld)	RR[653]	0.26	0.81	2.29	57
Collaroy (Qld)	RR[654]	0.32	0.95	2.79	9
College View	RR[655]	0.25	0.69	2.04	71
Collingwood Park (Qld)	RR[656]	0.29	0.56	0.99	5200
Collinsville (Qld)	RR[657]	0.76	1.69	3.33	1040
Colosseum	RR[658]	0.36	0.96	2.45	172
Columbia	RR[659]	0.40	1.13	3.09	80
Columboola	RR[660]	0.31	0.89	2.33	57
Comet	RR[661]	0.27	0.72	1.77	380
Commissioners Flat	RR[662]	0.25	0.75	2.38	21
Como (Qld)	RR[663]	0.29	0.79	2.13	40
Comoon Loop	RR[664]	0.45	1.37	3.84	48
Cona Creek	RR[665]	0.34	0.94	2.75	58
Condamine	RR[666]	0.46	1.21	2.82	310
Condamine Farms	RR[667]	0.26	0.82	2.56	23
Condamine Plains	RR[668]	0.25	0.76	2.10	83
Condon	RR[669]	0.47	0.84	1.40	4533
Conjuboy	RR[670]	0.41	1.31	3.66	3
Conondale	RR[671]	0.29	0.73	1.76	698
Consuelo	RR[672]	0.35	0.99	2.97	30
Conway	RR[673]	0.31	0.84	2.45	156
Conway Beach	RR[674]	0.27	0.87	2.81	72
Coochiemudlo Island	RR[675]	0.67	1.76	3.73	677
Coochin	RR[676]	0.23	0.67	1.76	87
Coochin Creek	RR[677]	0.27	0.73	2.09	68
Cooee Bay	RR[678]	0.38	0.97	2.07	751
Cooeeimbardi	RR[679]	0.41	1.17	3.40	7
Cooktown	RR[680]	**1.87**	**3.04**	**4.65**	2110
Coolabine	RR[681]	0.25	0.80	2.40	65
Coolabunia	RR[682]	0.27	0.77	2.22	133
Cooladdi	RR[683]	0.41	1.14	3.21	13
Coolana	RR[684]	0.23	0.67	1.88	154
Coolangatta (Qld)	RR[685]	**1.02**	**1.60**	**2.29**	5461
Coolbie	RR[686]	0.36	1.03	3.24	81
Coolmunda	RR[687]	0.51	1.45	4.08	111
Cooloola	RR[688]	0.31	0.83	2.39	3
Cooloola Cove	RR[689]	0.34	0.76	1.47	2269
Cooloolabin	RR[690]	0.25	0.74	1.87	219
Coolum Beach	RR[691]	0.57	0.90	1.40	6967
Coombabah	RR[692]	0.65	0.98	1.45	8737
Coomera	RR[693]	0.16	0.30	0.52	9454
Coominglah	RR[694]	0.31	0.85	2.39	15
Coominglah Forest	RR[695]	0.29	0.86	2.36	0
Coominya	RR[696]	0.70	1.47	2.97	977
Coomoo	RR[697]	0.33	0.96	2.77	23
Coomrith	RR[698]	0.30	0.93	2.71	32
Coonambula	RR[699]	0.31	0.87	2.68	54

Coonarr	RR[700]	0.36	1.14	3.10	204
Coondoo	RR[701]	0.28	0.79	2.21	66
Coongoola	RR[702]	0.37	1.14	3.77	10
Coopers Plains	RR[703]	0.63	1.06	1.68	4654
Coorada	RR[704]	0.35	0.96	2.71	6
Cooran	RR[705]	0.23	0.57	1.19	1298
Cooranga	RR[706]	0.26	0.77	2.14	102
Cooroibah	RR[707]	0.23	0.55	1.19	1656
Cooroo Lands	RR[708]	0.33	1.18	4.35	3
Coorooman	RR[709]	0.70	1.86	5.19	80
Cooroy	RR[710]	0.80	1.34	2.13	3111
Cooroy Mountain	RR[711]	0.37	1.06	3.19	112
Coorparoo	RR[712]	0.60	0.86	1.18	13745
Coorumba	RR[713]	0.39	1.12	2.98	106
Coorumbene	RR[714]	0.34	1.04	2.97	2
Cootharaba	RR[715]	0.28	0.71	1.54	690
Coowonga	RR[716]	0.36	1.01	2.55	211
Cooya Beach	RR[717]	0.86	2.17	4.77	450
Cooyar	RR[718]	0.27	0.76	1.98	194
Coppabella (Qld)	RR[719]	0.32	0.84	2.08	430
Coquette Point	RR[720]	0.55	1.73	5.47	74
Coral Cove	RR[721]	0.22	0.58	1.41	1003
Coral Sea	RR[722]	0.37	1.24	3.93	111
Coralie	RR[723]	0.48	1.48	4.79	6
Cordalba	RR[724]	0.49	1.29	3.16	365
Cordelia	RR[725]	0.60	1.59	4.48	174
Corella	RR[726]	0.27	0.74	2.27	49
Corfield	RR[727]	0.44	1.31	3.35	154
Corinda	RR[728]	0.56	0.97	1.59	4045
Coringa	RR[729]	0.34	0.89	2.54	68
Corndale (Qld)	RR[730]	0.27	0.82	2.64	39
Cornish Creek	RR[731]	0.44	1.30	3.74	16
Cornubia	RR[732]	0.28	0.52	0.89	5855
Cornwall (Qld)	RR[733]	0.36	1.09	3.14	11
Cosgrove (Qld)	RR[734]	0.24	0.77	1.94	234
Cotherstone	RR[735]	0.23	0.79	2.55	0
Cotswold Hills	RR[736]	0.24	0.63	1.41	1041
Cottonvale (Qld)	RR[737]	0.26	0.82	2.23	127
Coulson	RR[738]	0.32	0.86	2.31	161
Coverty	RR[739]	0.47	1.40	3.90	130
Cow Bay	RR[740]	0.46	1.49	4.21	181
Cowan Cowan	RR[741]	0.00	0.00	0.00	28
Cowley	RR[742]	0.39	1.15	3.09	70
Cowley Beach	RR[743]	0.39	1.20	3.47	68
Cowley Creek	RR[744]	0.41	1.26	3.95	10
Cracow	RR[745]	0.54	1.50	4.41	84
Craiglie	RR[746]	0.33	0.84	2.05	783
Craignish	RR[747]	0.44	0.94	1.90	1478
Cranbrook (Qld)	RR[748]	0.73	1.16	1.83	4887
Cranley	RR[749]	0.24	0.57	1.29	1272

Crawford	RR[750]	0.26	0.76	2.33	112
Crediton	RR[751]	0.28	0.81	2.13	66
Cremorne (Qld)	RR[752]	0.23	0.93	3.66	30
Cressbrook	RR[753]	0.29	0.83	2.20	98
Cressbrook Creek	RR[754]	0.30	0.88	2.44	20
Crestmead	RR[755]	0.35	0.57	0.93	8699
Crimea	RR[756]	0.31	1.05	3.14	11
Crinum	RR[757]	0.27	0.79	2.44	61
Croftby	RR[758]	0.23	0.67	1.75	114
Crohamhurst	RR[759]	0.24	0.67	2.01	184
Cromarty	RR[760]	0.41	1.24	3.81	21
Crossdale	RR[761]	0.37	1.01	2.69	69
Crossroads	RR[762]	0.29	0.82	2.23	92
Crowley Vale	RR[763]	0.21	0.70	2.01	71
Crownthorpe	RR[764]	0.29	0.83	2.52	38
Crows Nest (Qld)	RR[765]	0.85	1.59	2.78	1758
Croydon (Qld)	RR[766]	0.42	1.28	3.12	201
Cryna	RR[767]	0.24	0.70	2.09	100
Crystal Brook (Qld)	RR[768]	0.32	0.88	2.44	137
Crystal Creek (Qld)	RR[769]	0.36	1.07	3.33	6
Crystalbrook	RR[770]	0.45	1.21	3.79	11
Cullinane	RR[771]	0.48	1.15	2.83	449
Cungulla	RR[772]	0.52	1.78	5.00	254
Cunnamulla	RR[773]	0.61	1.33	2.77	873
Cunningham (Qld)	RR[774]	0.27	0.78	2.55	57
Curra	RR[775]	0.49	1.03	1.99	1510
Currajah	RR[776]	0.44	1.29	3.65	55
Currajong	RR[777]	**1.68**	**2.69**	**4.21**	2065
Curramore (Qld)	RR[778]	0.44	1.19	3.26	163
Currimundi	RR[779]	0.70	1.16	1.74	5437
Currumbin	RR[780]	**1.30**	**2.14**	**3.52**	2413
Currumbin Valley	RR[781]	0.51	1.13	2.15	1493
Currumbin Waters	RR[782]	0.74	1.13	1.69	7371
Curtis Island	RR[783]	0.03	0.71	3.91	323
Cushnie	RR[784]	0.29	0.81	2.42	105
Cutella	RR[785]	0.22	0.72	2.21	20
Cuttaburra	RR[786]	0.35	1.14	3.30	10
Cynthia	RR[787]	0.26	0.86	2.39	24
Cypress Gardens	RR[788]	0.25	0.82	2.61	85
Dagmar	RR[790]	0.44	1.45	4.24	0
D'Aguilar	RR[789]	0.30	0.79	1.81	924
Dagun	RR[791]	0.23	0.69	1.91	124
Daintree	RR[792]	0.40	1.22	3.61	113
Daisy Hill (Qld)	RR[793]	0.39	0.71	1.17	5240
Dajarra	RR[794]	0.59	1.71	4.93	133
Dakabin	RR[795]	0.30	0.65	1.31	2697
Dakenba	RR[796]	0.27	0.80	2.29	97
Dalbeg	RR[797]	0.36	1.05	3.00	64
Dalby	RR[798]	0.77	1.12	1.56	9748
Dalcouth	RR[799]	0.37	1.09	3.45	136

Dalga	RR[800]	0.25	0.84	2.57	3
Dallarnil	RR[801]	0.28	0.83	2.21	194
Dalma	RR[802]	0.32	0.98	2.69	64
Dalrymple Creek	RR[803]	0.40	1.21	3.40	85
Dalrymple Heights	RR[804]	0.26	0.85	2.73	41
Dalveen	RR[805]	0.27	0.75	2.00	289
Dalwogon	RR[806]	0.30	0.89	2.98	27
Dalysford	RR[807]	0.27	0.79	2.33	99
Damascus	RR[808]	0.28	0.80	2.36	89
Damper Creek	RR[809]	0.43	1.27	3.44	42
Danbulla	RR[810]	0.39	1.13	2.92	40
Danderoo	RR[811]	0.23	0.71	2.13	21
Dangore	RR[812]	0.40	1.15	3.33	29
Daradgee	RR[813]	0.40	1.13	3.38	65
Dargal Road	RR[814]	0.32	0.98	2.59	110
Darling Heights	RR[815]	0.45	0.80	1.41	4004
Darlington (Qld)	RR[816]	0.32	0.99	2.74	61
Darr Creek	RR[817]	0.32	0.90	2.62	18
Darra	RR[818]	0.40	0.77	1.33	3503
Darts Creek	RR[819]	0.54	1.55	4.31	125
Dauan Island	RR[820]	0.39	1.88	6.94	113
Daveson	RR[821]	0.33	1.18	3.76	17
Dayboro	RR[822]	0.29	0.69	1.43	1599
Daymar	RR[823]	0.28	0.92	2.78	25
Deagon	RR[824]	0.89	1.47	2.44	3023
Deception Bay	RR[825]	0.56	0.79	1.09	15559
Dedin	RR[826]	0.47	1.36	3.77	0
Deebing Heights	RR[827]	0.21	0.50	1.14	1526
Deep Creek (Qld)	RR[828]	0.31	0.94	2.82	13
Deepwater (Qld)	RR[829]	0.34	1.02	2.61	188
Deeragun	RR[830]	0.29	0.61	1.14	3096
Deeral	RR[831]	0.41	1.10	3.17	113
Degarra	RR[832]	0.42	1.45	4.47	85
Degilbo	RR[833]	0.29	0.88	2.47	140
Delan	RR[834]	0.27	0.76	2.03	247
Delaneys Creek	RR[835]	0.26	0.66	1.45	858
Depot Hill	RR[836]	**1.11**	**2.25**	**4.10**	850
Derri Derra	RR[837]	0.33	0.84	2.22	119
Derrymore	RR[838]	0.24	0.70	1.98	68
Desailly	RR[839]	0.44	1.27	4.44	0
Deuchar	RR[840]	0.33	0.89	2.44	235
Devereux Creek	RR[841]	0.27	0.70	1.83	322
Devon Park (Qld)	RR[842]	0.25	0.77	2.22	38
Diamantina Lakes	RR[843]	0.48	1.41	4.13	13
Diamond Valley	RR[844]	0.20	0.57	1.56	408
Diamondvale	RR[845]	0.27	0.91	3.08	44
Diamondy	RR[846]	0.32	0.86	2.37	42
Dicky Beach	RR[847]	0.34	0.79	1.58	1612
Didcot	RR[848]	0.32	0.94	2.91	45
Diddillibah	RR[849]	0.30	0.72	1.51	1129

Diglum	RR[850]	0.28	0.77	2.11	33
Dimbulah	RR[851]	0.63	1.42	2.92	866
Dingo	RR[852]	0.42	1.02	2.70	271
Dingo Beach	RR[853]	0.27	1.03	3.68	155
Dingo Pocket	RR[854]	0.40	1.13	3.24	75
Dinmore	RR[855]	0.37	0.91	2.11	690
Dirnbir	RR[856]	0.32	0.93	2.60	43
Dirranbandi	RR[857]	0.35	0.87	2.14	521
Dittmer	RR[858]	0.31	0.91	3.03	62
Diwan	RR[859]	0.50	1.57	4.93	137
Dixalea	RR[860]	0.33	0.94	2.78	68
Dixie (Qld)	RR[861]	0.44	1.39	4.13	16
Djarawong	RR[862]	0.37	1.09	3.31	79
Djiru	RR[863]	0.41	1.21	3.47	0
Djuan	RR[864]	0.38	1.07	3.31	77
Doctor Creek	RR[865]	0.28	0.86	2.45	29
Dolphin Heads	RR[866]	0.22	0.75	2.20	324
Domville	RR[867]	0.24	0.80	2.81	0
Donnybrook (Qld)	RR[868]	0.87	2.07	4.58	558
Doolandella	RR[869]	0.21	0.45	0.88	3652
Doolbi	RR[870]	0.34	1.06	3.09	106
Doomadgee	RR[871]	0.37	1.00	2.31	861
Doonan	RR[872]	0.40	0.80	1.44	2758
Doongul	RR[873]	0.39	1.24	3.40	26
Dotswood	RR[874]	0.35	0.97	2.47	83
Doughboy	RR[875]	0.31	0.87	2.61	24
Douglas (Toowoomba - Qld)	RR[876]	0.25	0.72	2.12	90
Douglas (Townsville - Qld)	RR[877]	0.16	0.33	0.60	6534
Dowar Islet	RR[878]	0.18	1.34	7.75	0
Downsfield	RR[879]	0.30	0.83	2.45	74
Dows Creek	RR[880]	0.28	0.84	2.33	102
Draper	RR[881]	0.28	0.69	1.66	534
Drayton	RR[882]	0.28	0.64	1.40	1402
Drewvale	RR[883]	0.25	0.48	0.95	3264
Drillham	RR[884]	0.30	0.88	2.48	94
Drillham South	RR[885]	0.31	0.93	2.51	53
Drinan	RR[886]	0.25	0.80	2.07	136
Dromedary (Qld)	RR[887]	0.34	0.98	2.79	4
Drummondslope	RR[888]	0.35	1.10	3.18	27
Duaringa	RR[889]	0.39	1.08	2.95	221
Duchess	RR[890]	0.57	1.63	4.75	18
Duckinwilla	RR[891]	0.37	0.94	2.58	12
Ducklo	RR[892]	0.27	0.68	1.97	249
Dugandan	RR[893]	0.22	0.61	1.54	475
Duingal	RR[894]	0.28	0.82	2.39	65
Dulacca	RR[895]	0.38	1.09	2.98	147
Dulong	RR[896]	0.38	0.97	2.29	474
Dululu	RR[897]	0.32	0.91	2.58	111
Dumbleton	RR[898]	0.31	0.88	2.45	202
Dumgree	RR[899]	0.30	0.88	2.48	46

Dumpy Creek	RR[900]	0.34	0.97	2.80	11
Dundarrah	RR[901]	0.29	0.98	2.95	3
Dundas (Qld)	RR[902]	0.27	0.74	2.15	63
Dundathu	RR[903]	0.24	0.79	2.21	198
Dundowran	RR[904]	0.24	0.60	1.46	727
Dundowran Beach	RR[905]	0.32	0.75	1.52	1734
Dunk	RR[906]	0.24	1.10	4.46	21
Dunkeld (Qld)	RR[907]	0.33	0.99	2.79	38
Dunmora	RR[908]	0.30	0.78	2.15	182
Dunmore (Qld)	RR[909]	0.27	0.84	2.38	19
Dunnrock	RR[910]	0.26	0.80	2.68	64
Dunrobin (Qld)	RR[911]	0.41	1.14	3.22	11
Dunwich	RR[912]	0.85	2.21	4.82	658
Durack (Qld)	RR[913]	0.57	0.93	1.45	6034
Durah	RR[914]	0.31	0.91	2.72	3
Durham	RR[915]	0.35	1.21	3.67	33
Durham Downs	RR[916]	0.33	0.98	3.02	57
Durong	RR[917]	0.38	1.10	2.87	190
Dutton Park	RR[918]	0.59	1.23	2.24	1729
Dutton River	RR[919]	0.47	1.38	3.92	29
Dykehead	RR[920]	0.33	0.94	2.83	5
Dynevor	RR[921]	0.40	1.16	3.57	17
Dysart (Qld)	RR[922]	0.30	0.66	1.32	2200
Eagle Farm	RR[923]	0.53	1.53	4.84	0
Eagleby	RR[924]	0.47	0.71	1.04	10446
Eaglefield	RR[925]	0.29	0.90	2.66	0
Earlville	RR[926]	1.00	1.58	2.54	3296
East Barron	RR[927]	0.45	1.22	3.11	174
East Brisbane	RR[928]	**1.14**	**1.69**	**2.45**	5218
East Cooyar	RR[929]	0.27	0.81	2.67	17
East Creek	RR[930]	0.45	1.57	5.14	0
East Deep Creek	RR[931]	0.23	0.59	1.45	536
East End	RR[932]	0.30	0.87	2.55	47
East Feluga	RR[933]	0.35	0.98	2.59	205
East Greenmount	RR[934]	0.23	0.63	1.52	304
East Haldon	RR[935]	0.23	0.74	2.00	20
East Innisfail	RR[936]	0.73	1.54	2.95	1452
East Ipswich	RR[937]	0.49	1.00	1.83	1920
East Mackay	RR[938]	0.60	1.11	1.91	2953
East Nanango	RR[939]	0.30	0.77	2.16	228
East Palmerston	RR[940]	0.39	1.04	3.03	153
East Russell	RR[941]	0.42	1.16	3.41	61
East Toowoomba	RR[942]	0.91	1.46	2.29	4187
East Trinity	RR[943]	0.38	1.07	2.87	179
Eastern Heights	RR[944]	0.80	1.41	2.31	3018
Eaton (Qld)	RR[945]	0.37	1.17	3.36	36
Eatons Hill	RR[946]	0.24	0.46	0.81	6036
Ebbw Vale	RR[947]	0.25	0.66	1.75	422
Ebenezer (Qld)	RR[948]	0.21	0.61	1.62	260
Edens Landing	RR[949]	0.23	0.48	0.87	3898

Edge Hill	RR[950]	0.64	1.10	1.87	3157
Edmonton	RR[951]	0.70	1.06	1.57	7887
Edward River	RR[952]	0.58	1.76	5.58	0
Eerwah Vale	RR[953]	0.30	0.77	1.98	499
Egypt	RR[954]	0.23	0.67	2.01	15
Eidsvold	RR[955]	0.34	0.88	2.20	462
Eidsvold East	RR[956]	0.31	0.84	2.52	20
Eidsvold West	RR[957]	0.34	0.88	2.38	56
Eight Mile Creek (Qld)	RR[958]	0.39	1.10	3.28	0
Eight Mile Plains	RR[959]	0.30	0.48	0.72	12755
Eimeo	RR[960]	0.33	0.73	1.47	2418
Einasleigh	RR[961]	0.54	1.56	4.24	78
El Arish	RR[962]	0.47	1.20	2.95	295
Elaman Creek	RR[963]	0.27	0.76	2.43	65
Elanora	RR[964]	0.59	0.90	1.32	9823
Elbow Valley	RR[965]	0.24	0.74	2.31	77
Electra	RR[966]	0.31	0.87	2.60	91
Elgin (Qld)	RR[967]	0.33	0.98	2.74	30
Elgin Vale	RR[968]	0.29	0.86	2.67	21
Eli Waters	RR[969]	0.28	0.61	1.17	2625
Elimbah	RR[970]	0.39	0.75	1.30	3174
Ellangowan (Qld)	RR[971]	0.42	1.27	3.35	102
Ellen Grove	RR[972]	0.17	0.40	0.87	2147
Ellerbeck	RR[973]	0.38	1.04	3.01	171
Ellesmere	RR[974]	0.44	1.21	3.11	286
Ellinjaa	RR[975]	0.36	1.16	3.51	22
Ellinthorp	RR[976]	0.20	0.81	3.17	15
Elliott (Qld)	RR[977]	0.35	0.94	2.62	106
Elliott Heads	RR[978]	0.81	1.70	3.53	874
Ellis Beach	RR[979]	0.40	1.65	6.81	21
Elphinstone (Isaac - Qld)	RR[980]	0.27	0.86	2.56	6
Elphinstone (Toowoomba - Qld)	RR[981]	0.28	0.79	2.49	56
Emerald (Qld)	RR[982]	0.39	0.61	0.92	10562
Emu Creek (Qld)	RR[983]	0.29	0.80	2.37	63
Emu Park	RR[984]	**1.14**	**1.95**	**3.31**	1798
Emu Vale	RR[985]	0.23	0.68	1.68	156
England Creek	RR[986]	0.28	0.77	2.23	24
Enoggera	RR[987]	0.58	1.01	1.60	4290
Enoggera Reservoir	RR[988]	0.26	0.74	1.97	19
Epsom (Qld)	RR[989]	0.27	0.86	2.71	6
Erakala	RR[990]	0.22	0.64	1.64	416
Ernestina	RR[991]	0.48	1.43	4.51	12
Eromanga	RR[992]	0.40	1.18	3.08	97
Erub Island	RR[993]	0.23	1.27	4.57	231
Esk (Qld)	RR[994]	0.96	1.81	3.31	1435
Eskdale (Qld)	RR[995]	0.29	0.83	2.25	30
Esmeralda	RR[996]	0.49	1.39	4.00	15
Etna Creek	RR[997]	0.40	0.95	2.12	739
Eton	RR[998]	0.33	0.76	1.93	538
Etty Bay	RR[999]	0.38	1.05	2.80	306

Eubenangee	RR[1000]	0.37	0.99	2.62	197
Eudlo	RR[1001]	0.29	0.75	1.70	892
Eukey	RR[1002]	0.29	0.91	2.45	119
Euleilah	RR[1003]	0.27	0.75	2.27	159
Eulo	RR[1004]	0.35	1.12	3.24	84
Eumamurrin	RR[1005]	0.36	0.97	2.63	77
Eumundi	RR[1006]	0.55	1.13	2.15	1765
Eungella (Qld)	RR[1007]	0.27	0.79	2.33	157
Eungella Dam	RR[1008]	0.32	0.88	2.60	8
Eungella Hinterland	RR[1009]	0.38	1.02	2.65	3
Euramo	RR[1010]	0.38	1.07	3.17	108
Eureka (Qld)	RR[1011]	0.35	0.94	2.69	135
Eurella	RR[1012]	0.37	1.05	3.26	11
Eurimbula	RR[1013]	0.28	0.85	2.46	4
Eurombah	RR[1014]	0.33	0.94	2.52	58
Eurong	RR[1015]	0.00	0.00	0.00	47
Euthulla	RR[1016]	0.40	1.05	2.62	271
Evans Landing	RR[1017]	0.26	0.97	3.28	59
Evanslea	RR[1018]	0.24	0.72	2.17	32
Evelyn	RR[1019]	0.37	0.96	2.51	209
Evergreen	RR[1020]	0.28	0.81	2.52	36
Everton Hills	RR[1021]	0.40	0.74	1.24	4647
Everton Park	RR[1022]	0.69	1.05	1.52	7345
Evora	RR[1023]	0.37	1.24	3.75	0
Fairdale	RR[1024]	0.27	0.82	2.62	26
Fairfield (Qld)	RR[1025]	0.46	0.91	1.64	2602
Fairney View	RR[1026]	0.26	0.72	2.04	188
Fairy Bower	RR[1027]	0.36	1.10	3.15	77
Fairyland	RR[1028]	0.28	0.83	2.44	51
Fairymead	RR[1029]	0.33	0.92	2.56	33
Farleigh	RR[1030]	0.27	0.69	1.66	654
Farnborough	RR[1031]	0.26	0.74	1.83	403
Farnsfield	RR[1032]	0.37	1.03	2.71	97
Farrars Creek	RR[1033]	0.46	1.39	4.11	0
Fassifern (Qld)	RR[1034]	0.23	0.70	2.16	18
Fassifern Valley	RR[1035]	0.23	0.74	2.20	66
Federal (Qld)	RR[1036]	0.31	0.86	2.22	251
Felton	RR[1037]	0.26	0.71	1.72	228
Felton South	RR[1038]	0.28	0.80	2.20	49
Feluga	RR[1039]	0.35	0.98	2.58	197
Ferney	RR[1040]	0.26	0.85	2.53	60
Fernvale (Qld)	RR[1041]	0.53	1.07	1.92	2335
Ferny Glen	RR[1042]	0.24	0.71	2.16	72
Ferny Grove	RR[1043]	0.24	0.50	0.93	4545
Ferny Hills	RR[1044]	0.47	0.76	1.20	6834
Ficks Crossing	RR[1045]	0.29	0.85	2.67	33
Fielding	RR[1046]	0.49	1.57	4.51	6
Fifteen Mile	RR[1047]	0.24	0.74	2.07	22
Fig Tree Pocket	RR[1048]	0.49	0.97	1.67	3081
Finch Hatton	RR[1049]	0.31	0.87	2.15	410

Finlayvale	RR[1050]	0.45	1.38	4.46	15
Finnie	RR[1051]	0.22	0.65	1.88	84
Fisher (Qld)	RR[1052]	0.50	1.48	4.74	4
Fishermans Pocket	RR[1053]	0.27	0.75	2.10	24
Fishery Falls	RR[1054]	0.48	1.44	3.78	123
Fitzgerald Creek	RR[1055]	0.38	1.18	3.43	64
Fitzgibbon	RR[1056]	0.31	0.59	1.06	4542
Fitzroy Island	RR[1057]	0.40	1.46	5.43	44
Flagstone Creek	RR[1058]	0.23	0.62	1.69	184
Flametree	RR[1059]	0.23	0.75	2.12	106
Flaxton	RR[1060]	0.52	1.18	2.78	835
Fletcher (Qld)	RR[1061]	0.29	0.88	2.63	83
Fletcher Creek	RR[1062]	0.37	1.09	3.22	0
Fleurbaix	RR[1063]	0.34	1.12	3.39	33
Flinders View	RR[1064]	0.63	1.09	1.68	4519
Flinton	RR[1065]	0.30	0.88	2.53	25
Florence Bay	RR[1066]	0.60	2.74	11.10	0
Flying Fish Point	RR[1067]	0.82	2.33	5.66	343
Flying Fox (Qld)	RR[1068]	0.25	0.71	2.33	52
Fordsdale	RR[1069]	0.21	0.66	2.03	46
Foreshores	RR[1070]	0.24	0.76	2.39	111
Forest Creek	RR[1071]	0.42	1.25	3.54	88
Forest Glen (Qld)	RR[1072]	0.25	0.61	1.38	1077
Forest Hill (Qld)	RR[1073]	0.30	0.77	1.77	803
Forest Lake	RR[1074]	0.19	0.31	0.49	17847
Forest Ridge	RR[1075]	0.25	0.80	2.60	41
Forest Springs	RR[1076]	0.27	0.77	2.15	55
Forestdale	RR[1077]	0.33	0.77	1.53	2039
Foresthome	RR[1078]	0.44	1.37	3.94	73
Forestvale	RR[1079]	0.36	1.07	2.89	39
Fork Lagoons	RR[1080]	0.29	0.86	2.44	0
Formartin	RR[1081]	0.26	0.75	2.17	67
Forrest Beach (Qld)	RR[1082]	0.71	1.57	3.13	1073
Forsayth	RR[1083]	0.41	1.20	3.29	103
Fortitude Valley	RR[1084]	0.48	0.79	1.25	6681
Forty Mile	RR[1085]	0.43	1.19	3.69	12
Fossilbrook	RR[1086]	0.42	1.29	4.32	2
Foulden	RR[1087]	0.25	0.82	2.49	3
Four Ways	RR[1088]	0.49	1.63	5.19	15
Foxdale	RR[1089]	0.28	0.86	2.43	97
Frankfield	RR[1090]	0.33	0.96	3.09	25
Fraser Island	RR[1091]	0.00	0.00	0.00	172
Frazerview	RR[1092]	0.24	0.69	2.16	31
Fredericksfield	RR[1093]	0.34	0.98	2.65	182
Freestone	RR[1094]	0.22	0.65	1.85	179
Frenches Creek	RR[1095]	0.20	0.69	2.18	74
Frenchville	RR[1096]	0.42	0.70	1.16	7205
Freshwater (Qld)	RR[1097]	0.40	0.86	1.78	1638
Freshwater Point	RR[1098]	0.29	0.81	2.52	123
Friday Pocket	RR[1099]	0.37	1.11	3.34	31

Fulham (Qld)	RR[1100]	0.37	1.10	3.11	27
Gadgarra	RR[1101]	0.37	1.09	3.10	3
Gaeta	RR[1102]	0.26	0.78	2.30	105
Gailes	RR[1103]	0.37	0.82	1.69	1443
Gainsford	RR[1104]	0.34	0.96	2.69	11
Gairloch	RR[1105]	0.45	1.32	4.23	37
Galilee	RR[1106]	0.38	1.11	3.44	16
Gamboola	RR[1107]	0.44	1.31	4.05	20
Gangalidda	RR[1108]	0.33	1.48	6.89	0
Garbutt	RR[1109]	**1.15**	**1.94**	**3.22**	1985
Garfield (Qld)	RR[1110]	0.39	1.17	3.66	29
Gargett	RR[1111]	0.34	1.00	2.52	197
Garnant	RR[1112]	0.42	1.22	3.39	87
Garners Beach	RR[1113]	0.37	1.21	4.26	25
Garradunga	RR[1114]	0.37	1.07	2.96	116
Garrawalt	RR[1115]	0.40	1.25	3.22	0
Gatton	RR[1116]	0.93	1.40	2.04	5846
Gaven	RR[1117]	0.25	0.58	1.15	1299
Gayndah	RR[1118]	0.37	0.81	1.60	1666
Gaythorne	RR[1119]	0.43	0.88	1.59	2541
Geebung	RR[1120]	**1.06**	**1.69**	**2.54**	3667
Geham	RR[1121]	0.29	0.77	1.81	380
Gemini Mountains	RR[1122]	0.27	0.82	2.43	42
Georgetown (Qld)	RR[1123]	0.48	1.30	3.27	272
Georgina (Qld)	RR[1124]	0.53	1.49	4.36	7
Germantown (Qld)	RR[1125]	0.35	1.15	3.53	53
Gheerulla	RR[1126]	0.34	0.93	2.33	184
Ghinghinda	RR[1127]	0.33	0.96	2.79	33
Gidya	RR[1128]	0.52	1.65	4.73	0
Gigoomgan	RR[1129]	0.32	0.85	2.50	29
Gilbert River	RR[1130]	0.45	1.38	3.75	35
Gilberton (Etheridge - Qld)	RR[1131]	0.45	1.35	3.91	4
Gilberton (Gold Coast - Qld)	RR[1132]	0.21	0.67	1.93	20
Gilla	RR[1133]	0.31	0.85	2.37	28
Gilldora	RR[1134]	0.23	0.70	2.20	32
Gilston	RR[1135]	0.38	0.75	1.47	1839
Gin Gin (Qld)	RR[1136]	0.42	0.98	2.14	882
Gindie	RR[1137]	0.30	0.83	2.22	167
Gindoran	RR[1138]	0.26	0.84	2.35	3
Ginoondan	RR[1139]	0.33	0.97	2.82	13
Girraween (Qld)	RR[1140]	0.32	1.00	2.96	9
Giru	RR[1141]	**1.06**	**2.61**	**5.62**	302
Givelda	RR[1142]	0.30	0.90	2.54	42
Gladfield (Qld)	RR[1143]	0.25	0.69	2.22	44
Gladstone Central	RR[1144]	**2.16**	**3.64**	**5.74**	1355
Gladstone Harbour	RR[1145]	0.04	1.30	10.74	29
Glamorgan Vale	RR[1146]	0.23	0.65	1.76	357
Glan Devon	RR[1147]	0.37	1.01	2.63	211
Glanmire (Qld)	RR[1148]	0.25	0.72	2.16	20
Glass House Mountains	RR[1149]	0.45	0.86	1.48	4114

Glastonbury	RR[1150]	0.33	0.84	2.02	291
Glebe (Qld)	RR[1151]	0.30	0.98	2.76	16
Glen Allyn	RR[1152]	0.34	1.05	2.77	124
Glen Aplin	RR[1153]	0.32	0.94	2.43	405
Glen Boughton	RR[1154]	0.27	1.14	4.68	24
Glen Cairn	RR[1155]	0.23	0.67	1.92	95
Glen Echo	RR[1156]	0.30	0.90	2.56	27
Glen Eden	RR[1157]	0.23	0.55	1.12	2037
Glen Esk	RR[1158]	0.31	0.90	2.72	50
Glen Isla	RR[1159]	0.39	1.24	3.37	29
Glen Niven	RR[1160]	0.22	0.80	2.98	68
Glen Russell	RR[1161]	0.37	1.15	3.47	7
Glen Ruth	RR[1162]	0.40	1.19	3.39	0
Glenarbon	RR[1163]	0.31	0.96	3.07	36
Glenaubyn	RR[1164]	0.31	0.91	2.71	30
Glenaven	RR[1165]	0.29	0.84	2.49	61
Glenbar	RR[1166]	0.29	0.89	2.63	11
Glencoe (Qld)	RR[1167]	0.22	0.60	1.72	253
Glendale (Qld)	RR[1168]	0.31	0.88	2.12	488
Glenden	RR[1169]	0.41	1.08	2.48	466
Gleneagle	RR[1170]	0.31	0.73	1.62	1403
Glenella	RR[1171]	0.33	0.68	1.27	3506
Glenfern (Qld)	RR[1172]	0.28	0.80	2.33	10
Glengallan	RR[1173]	0.23	0.79	2.52	43
Glenhaughton	RR[1174]	0.32	0.98	2.95	18
Glenlee (Qld)	RR[1175]	0.28	0.69	1.59	1013
Glenleigh	RR[1176]	0.29	0.83	2.35	27
Glenlyon (Qld)	RR[1177]	0.31	0.94	2.87	21
Glenmoral	RR[1178]	0.34	0.94	2.52	37
Glenmorgan	RR[1179]	0.37	1.10	3.10	116
Glenorchy (Qld)	RR[1180]	0.30	0.85	2.45	72
Glenore Grove	RR[1181]	0.33	0.82	1.93	724
Glenrae	RR[1182]	0.31	0.94	2.50	70
Glenrock (Qld)	RR[1183]	0.27	0.85	2.74	37
Glenroy (Qld)	RR[1184]	0.36	0.95	2.82	22
Glenvale	RR[1185]	0.43	0.75	1.26	5025
Glenview	RR[1186]	0.22	0.57	1.39	949
Glenwood (Qld)	RR[1187]	0.44	1.00	2.16	1354
Godwin Beach	RR[1188]	0.23	0.68	1.84	365
Gogango	RR[1189]	0.31	0.96	2.70	96
Golden Beach (Qld)	RR[1190]	**1.02**	**1.58**	**2.33**	4823
Golden Fleece	RR[1191]	0.33	0.93	2.61	42
Goldfields	RR[1192]	0.29	0.85	2.64	28
Goldsborough (Qld)	RR[1193]	0.36	0.93	2.10	685
Gooburrum	RR[1194]	0.52	1.11	2.25	1153
Good Night	RR[1195]	0.31	0.87	2.43	112
Goodar	RR[1196]	0.30	0.88	2.63	45
Goodger	RR[1197]	0.27	0.79	2.23	150
Goodna	RR[1198]	0.53	0.83	1.24	7716
Goodwood (Qld)	RR[1199]	0.34	1.00	2.47	140

Googa Creek	RR[1200]	0.28	0.84	2.63	39
Goolboo	RR[1201]	0.43	1.23	3.68	16
Goolman	RR[1202]	0.29	0.90	2.75	33
Goomally	RR[1203]	0.32	0.94	2.79	15
Goombi	RR[1204]	0.26	0.90	2.92	23
Goomboorian	RR[1205]	0.54	1.29	3.07	418
Goombungee	RR[1206]	0.33	0.82	1.78	804
Goomburra	RR[1207]	0.24	0.67	1.79	209
Goomeri	RR[1208]	0.40	0.96	2.28	551
Goomeribong	RR[1209]	0.26	0.83	2.40	34
Goondi	RR[1210]	0.41	1.18	3.49	44
Goondi Bend	RR[1211]	0.42	1.17	2.70	445
Goondi Hill	RR[1212]	0.46	1.20	3.19	434
Goondiwindi	RR[1213]	0.68	1.17	1.80	4931
Goorganga Creek	RR[1214]	0.32	1.01	3.00	3
Goorganga Plains	RR[1215]	0.35	0.99	2.95	0
Gooroolba	RR[1216]	0.40	1.26	3.95	12
Gootchie	RR[1217]	0.32	0.93	2.76	82
Goovigen	RR[1218]	0.29	0.84	2.34	183
Goowarra	RR[1219]	0.34	0.95	2.60	16
Goranba	RR[1220]	0.28	0.76	2.30	157
Gordon Park	RR[1221]	0.57	1.00	1.67	3488
Gordonbrook	RR[1222]	0.31	0.81	2.29	139
Gordonstone	RR[1223]	0.26	0.84	2.66	3
Gordonvale	RR[1224]	**1.26**	**1.88**	**2.61**	5111
Gore	RR[1225]	0.29	0.88	2.66	33
Gowrie Junction	RR[1226]	0.25	0.58	1.31	1585
Gowrie Little Plain	RR[1227]	0.21	0.69	1.95	52
Gowrie Mountain	RR[1228]	0.29	0.86	2.41	176
Gowrie Station	RR[1229]	0.38	1.15	3.74	12
Gracemere	RR[1230]	0.48	0.75	1.12	8262
Graceville	RR[1231]	0.47	0.89	1.52	3585
Grahams Creek	RR[1232]	0.25	0.78	2.16	136
Granadilla	RR[1233]	0.38	1.07	3.28	62
Grand Secret	RR[1234]	0.41	1.18	3.21	126
Grandchester	RR[1235]	0.38	0.97	2.29	367
Grange (Qld)	RR[1236]	0.74	1.22	2.01	3212
Granite Vale	RR[1237]	0.33	0.95	2.72	0
Grant	RR[1238]	0.41	1.19	3.96	11
Grantham	RR[1239]	0.22	0.58	1.35	519
Granville (Qld)	RR[1240]	0.74	1.43	2.48	2200
Grapetree	RR[1241]	0.30	0.83	2.34	24
Grassdale (Qld)	RR[1242]	0.26	0.78	2.15	4
Grasstree Beach	RR[1243]	0.37	0.95	2.38	618
Grays Gate	RR[1244]	0.27	0.85	2.46	28
Great Sandy Strait	RR[1245]	0.26	0.94	3.11	4
Green Hill	RR[1246]	0.37	1.11	3.11	139
Green Island	RR[1247]	0.41	1.53	6.16	25
Greenbank	RR[1248]	0.55	0.90	1.46	6061
Greenlake	RR[1249]	0.34	0.98	2.88	0

Greenlands (Qld)	RR[1250]	0.35	1.04	2.82	205
Greenmount (Mackay - Qld)	RR[1251]	0.24	0.63	1.67	379
Greenmount (Toowoomba - Qld)	RR[1252]	0.42	1.10	2.48	550
Greens Creek (Qld)	RR[1253]	0.22	0.64	1.72	271
Greenslopes	RR[1254]	0.58	0.90	1.36	7750
Greenswamp	RR[1255]	0.28	0.86	2.57	37
Greenup	RR[1256]	0.31	0.95	2.96	20
Greenvale (Qld)	RR[1257]	0.66	1.69	4.22	196
Greenview	RR[1258]	0.28	0.83	2.46	72
Greenwood (Qld)	RR[1259]	0.31	1.04	3.16	28
Gregors Creek	RR[1260]	0.30	0.81	2.32	65
Gregory (Qld)	RR[1261]	0.52	1.48	4.38	59
Gregory River (Bundaberg - Qld)	RR[1262]	0.37	0.98	2.75	49
Gregory River (Whitsunday - Qld)	RR[1263]	0.28	0.79	2.12	248
Greycliffe	RR[1264]	0.31	0.89	2.67	15
Greymare	RR[1265]	0.27	0.85	2.50	51
Griffin	RR[1266]	0.19	0.38	0.71	4999
Groganville	RR[1267]	0.43	1.27	3.64	7
Groomsville	RR[1268]	0.26	0.75	1.98	86
Groper Creek	RR[1269]	0.48	1.52	4.52	62
Grosmont	RR[1270]	0.32	0.93	2.57	123
Grosvenor	RR[1271]	0.29	0.86	2.73	17
Guanaba	RR[1272]	0.38	0.95	2.13	676
Guijar Islet	RR[1273]	0.27	1.82	8.56	0
Gulf Of Carpentaria	RR[1274]	0.28	1.33	7.09	0
Gulliver	RR[1275]	**1.73**	**2.73**	**4.21**	2279
Gulngai	RR[1276]	0.39	1.13	3.47	0
Guluguba	RR[1277]	0.32	0.92	2.49	92
Gumdale	RR[1278]	0.22	0.52	1.16	1575
Gumlow	RR[1279]	0.28	0.77	2.25	131
Gumlu	RR[1280]	**1.19**	**3.19**	**7.79**	164
Gunalda	RR[1281]	0.42	1.13	2.87	342
Gundiah	RR[1282]	0.39	1.18	3.44	103
Gungaloon	RR[1283]	0.32	0.91	2.62	26
Gunnawarra	RR[1284]	0.40	1.17	3.43	10
Gunnewin	RR[1285]	0.37	1.06	3.04	44
Gunpowder	RR[1286]	0.55	1.55	4.25	43
Gunyarra	RR[1287]	0.30	0.99	2.97	13
Gurgeena	RR[1288]	0.31	0.96	2.91	27
Gurulmundi	RR[1289]	0.30	0.90	2.75	7
Guthalungra	RR[1290]	0.35	1.07	3.15	86
Gwambegwine	RR[1291]	0.31	1.01	3.07	17
Gympie	RR[1292]	**1.17**	**1.61**	**2.14**	8718
Habana	RR[1293]	0.26	0.67	1.52	764
Haden	RR[1294]	0.43	1.21	3.71	153
Haigslea	RR[1295]	0.29	0.79	1.98	397
Hail Creek	RR[1296]	0.30	0.81	2.27	145
Haliday Bay	RR[1297]	0.22	0.78	2.64	147
Halifax	RR[1298]	**1.36**	**3.09**	**6.22**	412
Halliford	RR[1299]	0.31	0.82	2.51	0

Haly Creek	RR[1300]	0.30	0.85	2.25	107
Hamilton (Qld)	RR[1301]	0.45	0.80	1.28	6251
Hamilton Creek	RR[1302]	0.37	1.06	3.18	86
Hamilton Plains	RR[1303]	0.29	0.93	2.69	67
Hampden (Qld)	RR[1304]	0.38	0.96	2.40	450
Hampton (Qld)	RR[1305]	0.63	1.57	3.93	294
Hannaford	RR[1306]	0.31	0.89	2.50	100
Happy Valley (Qld)	RR[1307]	0.60	1.48	3.13	555
Harlaxton	RR[1308]	0.31	0.63	1.29	2090
Harlin	RR[1309]	0.46	1.25	3.16	132
Harrami	RR[1310]	0.29	0.85	2.44	36
Harriet	RR[1311]	0.30	0.90	2.97	4
Harristown	RR[1312]	0.69	1.06	1.54	7045
Harrisville	RR[1313]	0.24	0.66	1.78	484
Hatton Vale	RR[1314]	0.30	0.69	1.50	1165
Hawkins Creek	RR[1315]	0.46	1.19	3.07	170
Hawkwood	RR[1316]	0.29	0.93	2.58	27
Hawthorne	RR[1317]	0.70	1.20	1.94	3928
Hay Point	RR[1318]	0.22	0.60	1.57	1081
Hazeldean	RR[1319]	0.27	0.73	1.91	232
Hazledean	RR[1320]	0.27	0.85	2.48	16
Headington Hill	RR[1321]	0.28	0.77	2.29	55
Healy	RR[1322]	0.44	0.95	1.99	1378
Heathwood	RR[1323]	0.15	0.35	0.78	2078
Heatley	RR[1324]	0.89	1.46	2.42	3240
Hebel	RR[1325]	0.38	1.31	4.25	63
Helens Hill	RR[1326]	0.39	1.12	3.28	104
Helensvale	RR[1327]	0.56	0.80	1.08	13735
Helidon	RR[1328]	0.31	0.76	1.80	808
Helidon Spa	RR[1329]	0.29	0.75	1.87	431
Hemmant	RR[1330]	0.53	1.08	2.05	1874
Hendon (Qld)	RR[1331]	0.28	0.75	2.16	159
Hendra	RR[1332]	**1.01**	**1.59**	**2.46**	3736
Herberton	RR[1333]	0.46	1.12	2.36	696
Heritage Park	RR[1334]	0.14	0.32	0.64	3845
Hermit Park	RR[1335]	**1.43**	**2.31**	**3.46**	2760
Herston	RR[1336]	0.62	1.18	2.10	1963
Hervey Range	RR[1337]	0.30	0.83	2.13	227
Hibernia	RR[1338]	0.28	0.83	2.57	42
Hidden Valley (Qld)	RR[1339]	0.27	0.80	1.96	336
Hideaway Bay	RR[1340]	0.23	0.76	2.66	183
Highbury (Qld)	RR[1341]	0.47	1.33	3.95	0
Highfields (Qld)	RR[1342]	0.25	0.49	0.85	6313
Highgate Hill	RR[1343]	0.80	1.24	1.85	5414
Highgrove	RR[1344]	0.28	0.82	2.37	19
Highland Park	RR[1345]	0.34	0.60	1.04	5338
Highland Plains (Maranoa - Qld)	RR[1346]	0.37	1.13	3.33	2
Highland Plains (Toowoomba - Qld)	RR[1347]	0.28	0.78	2.32	32
Highvale	RR[1348]	0.47	0.99	2.02	1371
Highworth	RR[1349]	0.58	1.52	3.95	240

Hillcrest (Qld)	RR[1350]	0.24	0.47	0.92	4341
Hillview	RR[1351]	0.25	0.77	2.33	58
Hinchinbrook (Qld)	RR[1352]	0.30	1.31	5.25	0
Hirstglen	RR[1353]	0.24	0.70	1.95	73
Hivesville	RR[1354]	0.34	1.03	2.78	149
Hobartville (Qld)	RR[1355]	0.39	1.10	3.31	40
Hodgleigh	RR[1356]	0.27	0.84	2.26	96
Hodgson	RR[1357]	0.32	0.93	3.04	40
Hodgson Vale	RR[1358]	0.18	0.48	1.10	1018
Holland Park	RR[1359]	**1.19**	**1.70**	**2.38**	6328
Holland Park West	RR[1360]	0.45	0.77	1.24	5251
Holloways Beach	RR[1361]	0.83	1.60	2.82	1952
Hollywell	RR[1362]	0.41	0.83	1.62	2400
Holmview	RR[1363]	0.18	0.46	1.02	1733
Holroyd River	RR[1364]	0.43	1.31	4.00	0
Home Creek	RR[1365]	0.43	1.24	3.64	20
Home Hill	RR[1366]	0.91	1.63	2.66	2439
Homebush (Qld)	RR[1367]	0.32	0.93	2.47	221
Homestead	RR[1368]	0.48	1.54	4.96	41
Hookswood	RR[1369]	0.32	0.90	2.28	48
Hope Island	RR[1370]	0.59	0.89	1.27	9574
Hope Vale	RR[1371]	0.29	0.79	1.88	736
Hopeland (Qld)	RR[1372]	0.29	0.81	2.26	110
Horn	RR[1373]	0.53	1.56	3.95	386
Horse Camp	RR[1374]	0.35	0.90	2.46	335
Horse Creek	RR[1375]	0.35	1.10	3.27	99
Horseshoe Bay	RR[1376]	0.61	1.70	4.01	512
Horseshoe Lagoon	RR[1377]	0.35	1.00	2.64	174
Horton	RR[1378]	0.44	1.26	3.34	137
Howard	RR[1379]	**1.37**	**2.48**	**4.37**	1176
Howitt	RR[1380]	0.53	1.56	4.32	24
Hoya	RR[1381]	0.23	0.68	1.92	189
Hudson	RR[1382]	0.46	1.38	3.91	169
Hughenden	RR[1383]	0.71	1.49	3.12	942
Hull Heads	RR[1384]	0.33	1.02	3.03	101
Humboldt	RR[1385]	0.30	0.96	2.69	10
Humeburn	RR[1386]	0.40	1.15	3.25	22
Humphery	RR[1387]	0.28	0.93	2.94	17
Hunchy	RR[1388]	0.28	0.77	1.99	462
Hungerford (Qld)	RR[1389]	0.36	1.13	3.48	20
Hurricane	RR[1390]	0.45	1.30	3.64	17
Hutton Creek	RR[1391]	0.37	1.09	3.09	20
Hyde Park (Qld)	RR[1392]	**1.54**	**2.87**	**4.96**	1100
Iama Island	RR[1393]	0.24	0.96	3.57	212
Ibis	RR[1394]	0.47	1.30	3.81	4
Idalia	RR[1395]	0.51	0.94	1.60	3372
Ideraway	RR[1396]	0.31	0.89	2.66	28
Ilbilbie	RR[1397]	0.44	1.26	3.39	297
Ilfracombe	RR[1398]	0.69	1.74	4.23	206
Ilkley	RR[1399]	0.21	0.54	1.33	629

Illinbah	RR[1400]	0.24	0.71	2.05	96
Image Flat	RR[1401]	0.32	0.87	2.22	357
Imbil	RR[1402]	0.44	1.06	2.33	756
Inala	RR[1403]	0.84	1.19	1.59	11333
Indooroopilly	RR[1404]	0.31	0.53	0.84	10466
Ingberry	RR[1405]	0.41	1.23	3.56	11
Ingham	RR[1406]	**1.76**	**2.58**	**3.70**	3722
Inglestone	RR[1407]	0.29	0.86	2.68	56
Inglewood (Qld)	RR[1408]	0.38	0.92	2.17	794
Ingoldsby	RR[1409]	0.23	0.67	1.89	54
Injinoo	RR[1410]	0.24	0.74	2.19	319
Injune	RR[1411]	**1.24**	**2.73**	**5.82**	383
Inkerman (Qld)	RR[1412]	0.36	1.09	2.98	120
Innes Park	RR[1413]	0.46	0.92	1.71	1758
Innisfail	RR[1414]	**1.64**	**3.11**	**5.49**	969
Innisfail Estate	RR[1415]	0.45	1.08	2.41	1067
Innisplain	RR[1416]	0.35	0.99	2.57	75
Innot Hot Springs	RR[1417]	0.38	1.01	2.57	151
Inskip	RR[1418]	0.18	0.80	3.73	45
Inverlaw	RR[1419]	0.36	0.99	2.60	170
Inverness	RR[1420]	0.38	0.98	2.43	369
Ipswich	RR[1421]	**3.14**	**4.64**	**6.61**	2104
Iredale	RR[1422]	0.22	0.68	1.73	115
Iron Range	RR[1423]	0.51	1.66	6.37	14
Ironbark (Qld)	RR[1424]	0.29	0.72	1.76	542
Irongate	RR[1425]	0.26	0.72	1.98	89
Ironpot (Livingstone - Qld)	RR[1426]	0.34	0.90	2.54	133
Ironpot (South Burnett - Qld)	RR[1427]	0.30	0.84	2.65	36
Irvinebank	RR[1428]	0.49	1.39	3.44	110
Irvingdale	RR[1429]	0.26	0.75	2.12	147
Isis Central	RR[1430]	0.40	1.15	3.00	170
Isis River	RR[1431]	0.34	0.97	2.75	101
Isisford	RR[1432]	0.42	1.13	3.09	180
Isla	RR[1433]	0.29	0.91	2.55	128
Island Plantation	RR[1434]	0.27	0.84	2.57	123
Iveragh	RR[1435]	0.24	0.72	2.15	104
Ivory Creek	RR[1436]	0.30	0.83	2.57	40
Jackson	RR[1437]	0.28	0.92	2.78	54
Jackson North	RR[1438]	0.30	0.90	2.84	43
Jackson South	RR[1439]	0.31	0.94	2.69	10
Jacobs Well	RR[1440]	0.50	1.08	2.20	1521
Jaffa	RR[1441]	0.38	1.17	3.34	18
Jaggan	RR[1442]	0.36	1.02	2.83	164
Jamboree Heights	RR[1443]	0.20	0.44	0.88	2469
Jandowae	RR[1444]	0.34	0.85	1.96	891
Japoonvale	RR[1445]	0.49	1.40	3.48	128
Jardine	RR[1446]	0.32	0.91	2.84	42
Jardine River	RR[1447]	0.30	0.94	3.06	0
Jarra Creek	RR[1448]	0.53	1.44	3.76	95
Jarvisfield	RR[1449]	0.43	1.14	3.00	281

Jeebropilly	RR[1450]	0.24	0.68	2.07	4
Jellinbah	RR[1451]	0.30	0.93	2.66	23
Jensen	RR[1452]	0.33	0.75	1.64	1130
Jericho (Qld)	RR[1453]	0.35	1.08	3.02	83
Jerona	RR[1454]	0.37	1.14	3.19	40
Jimboomba	RR[1455]	0.30	0.50	0.76	9919
Jimbour East	RR[1456]	0.25	0.74	2.14	154
Jimbour West	RR[1457]	0.26	0.83	2.54	38
Jimna	RR[1458]	0.28	0.75	2.03	75
Jindalee (Qld)	RR[1459]	0.52	0.94	1.56	4255
Jinghi	RR[1460]	0.31	0.81	2.30	56
Jobs Gate	RR[1461]	0.34	1.05	3.57	6
Johnsons Hill	RR[1462]	0.41	1.20	3.93	7
Johnstown	RR[1463]	0.28	0.84	2.46	27
Jollys Lookout	RR[1464]	0.23	0.71	2.00	63
Jondaryan	RR[1465]	0.25	0.68	1.70	296
Jones Gully	RR[1466]	0.29	0.81	2.51	7
Jones Hill	RR[1467]	0.26	0.67	1.55	689
Josephville	RR[1468]	0.21	0.65	1.87	138
Joskeleigh	RR[1469]	0.37	1.05	2.83	51
Joyner	RR[1470]	0.33	0.69	1.37	2171
Jubilee Heights	RR[1471]	0.39	1.11	3.09	112
Jubilee Pocket	RR[1472]	0.41	0.92	1.85	1418
Julago	RR[1473]	0.35	1.02	2.98	91
Julatten	RR[1474]	0.38	0.89	1.90	916
Julia Creek	RR[1475]	**1.35**	**3.07**	**6.24**	415
Junabee	RR[1476]	0.25	0.68	1.80	169
Junction View	RR[1477]	0.26	0.68	2.09	35
Jundah	RR[1478]	0.41	1.19	3.39	93
Kaban	RR[1479]	0.37	1.06	3.11	73
Kabra	RR[1480]	0.53	1.34	3.39	337
Kagaru	RR[1481]	0.22	0.64	1.85	10
Kaimkillenbun	RR[1482]	0.27	0.73	1.94	233
Kairabah	RR[1483]	0.20	0.64	2.04	0
Kairi	RR[1484]	0.34	0.91	2.50	352
Kalapa	RR[1485]	0.34	1.00	2.84	80
Kalbar	RR[1486]	0.66	1.46	2.93	858
Kalinga	RR[1487]	0.57	1.16	2.16	1627
Kalkadoon	RR[1488]	0.44	1.67	5.30	23
Kalkie	RR[1489]	0.46	0.97	1.75	2109
Kallangur	RR[1490]	0.56	0.80	1.10	15913
Kalpowar	RR[1491]	0.30	0.79	2.26	57
Kalunga	RR[1492]	0.46	1.32	3.88	81
Kamerunga	RR[1493]	0.42	1.01	2.24	834
Kandanga	RR[1494]	0.35	0.96	2.34	527
Kandanga Creek	RR[1495]	0.27	0.78	2.15	95
Kangaroo Point (Qld)	RR[1496]	0.90	1.33	1.87	7624
Kanigan	RR[1497]	0.31	0.89	2.67	101
Kanimbla (Qld)	RR[1498]	0.40	0.81	1.54	2037
Kapaldo	RR[1499]	0.26	0.80	2.50	36

Karalee	RR[1500]	0.25	0.53	1.06	3311
Karana Downs	RR[1501]	0.43	0.85	1.58	2971
Karara	RR[1502]	0.27	0.82	2.25	103
Karawatha	RR[1503]	0.29	0.75	2.15	16
Karrabin	RR[1504]	0.23	0.66	1.74	351
Karragarra Island	RR[1505]	0.42	1.60	5.50	187
Karron	RR[1506]	0.53	1.56	4.93	0
Karumba	RR[1507]	0.79	1.91	4.32	474
Kawana	RR[1508]	0.39	0.77	1.40	3519
Kawl Kawl	RR[1509]	0.29	0.88	2.66	13
Kawungan	RR[1510]	0.75	1.23	1.97	4000
Kearneys Spring	RR[1511]	0.50	0.81	1.24	6949
Kedron	RR[1512]	0.95	1.37	1.92	7535
Kelsey Creek	RR[1513]	0.32	0.89	2.68	100
Kelso (Qld)	RR[1514]	0.33	0.56	0.90	7907
Kelvin Grove	RR[1515]	0.47	0.78	1.22	6917
Kelvinhaugh	RR[1516]	0.26	0.78	2.20	37
Kemmis	RR[1517]	0.28	0.82	2.56	3
Kenilworth	RR[1518]	0.52	1.18	2.72	495
Kenmore	RR[1519]	0.71	1.09	1.58	7088
Kenmore Hills	RR[1520]	0.43	0.94	1.71	1996
Kennedy	RR[1521]	0.36	1.07	3.04	127
Kensington (Qld)	RR[1522]	0.51	1.19	2.83	486
Kensington Grove	RR[1523]	0.21	0.54	1.23	1309
Kents Lagoon	RR[1524]	0.23	0.70	2.16	37
Kents Pocket	RR[1525]	0.25	0.78	2.32	18
Kentville	RR[1526]	0.26	0.74	2.20	85
Keperra	RR[1527]	0.48	0.80	1.36	5452
Kepnock	RR[1528]	0.78	1.33	2.07	3603
Keppel Sands	RR[1529]	**1.07**	**2.57**	**6.03**	320
Keriri Island	RR[1530]	0.16	0.80	3.33	171
Kerry	RR[1531]	0.25	0.67	1.68	261
Kewarra Beach	RR[1532]	0.43	0.81	1.36	4461
Keysland	RR[1533]	0.27	0.88	2.73	12
Kholo	RR[1534]	0.26	0.67	1.75	318
Khosh Bulduk	RR[1535]	0.26	0.78	2.32	45
Kia Ora (Qld)	RR[1536]	0.27	0.78	2.27	159
Kiamba	RR[1537]	0.34	0.94	2.59	156
Kianga (Qld)	RR[1538]	0.29	0.86	2.45	173
Kidaman Creek	RR[1539]	0.26	0.79	2.33	115
Kiels Mountain	RR[1540]	0.21	0.59	1.63	522
Kilbirnie	RR[1541]	0.24	0.78	2.31	51
Kilcoy	RR[1542]	0.47	1.01	2.12	1504
Kilcummin	RR[1543]	0.28	0.79	2.20	185
Kilkivan	RR[1544]	0.30	0.80	1.92	587
Killaloe	RR[1545]	0.34	1.00	3.13	92
Killarney (Qld)	RR[1546]	0.29	0.78	1.84	805
Kilmorey Falls	RR[1547]	0.39	1.08	3.24	0
Kimberley (Qld)	RR[1548]	0.39	1.29	5.09	30
Kin Kin	RR[1549]	0.35	0.86	2.00	621

Kin Kora	RR[1550]	0.22	0.53	1.10	2042
Kinbombi	RR[1551]	0.28	0.82	2.43	27
Kinchant Dam	RR[1552]	0.25	0.79	2.40	92
Kincora	RR[1553]	0.27	0.81	2.35	38
Kindon	RR[1554]	0.27	0.88	2.55	19
King Scrub	RR[1555]	0.22	0.57	1.50	269
Kingaham	RR[1556]	0.29	0.81	2.18	10
Kingaroy	RR[1557]	0.75	1.10	1.60	7855
Kings Beach	RR[1558]	0.49	0.98	1.89	2504
Kings Creek	RR[1559]	0.26	0.78	2.24	56
Kings Siding	RR[1560]	0.24	0.74	2.30	16
Kingsholme	RR[1561]	0.21	0.59	1.52	212
Kingsthorpe	RR[1562]	0.34	0.74	1.50	1454
Kingston (Qld)	RR[1563]	0.72	1.09	1.56	8007
Kinka Beach	RR[1564]	0.58	1.46	3.19	570
Kinkuna	RR[1565]	0.35	0.99	2.62	91
Kinleymore	RR[1566]	0.32	0.85	2.43	77
Kinnoul	RR[1567]	0.33	0.97	2.83	55
Kioma	RR[1568]	0.28	0.88	2.61	27
Kippa-Ring	RR[1569]	0.47	0.77	1.18	7907
Kirknie	RR[1570]	0.38	1.07	2.97	50
Kirkwood	RR[1571]	0.17	0.43	1.03	1504
Kirrama	RR[1572]	0.41	1.21	3.43	4
Kirwan	RR[1573]	0.33	0.50	0.72	16721
Kitoba	RR[1574]	0.28	0.89	2.56	10
Kleinton	RR[1575]	0.16	0.44	1.04	1146
Knapp Creek	RR[1576]	0.25	0.72	2.10	48
Koah	RR[1577]	0.33	0.85	2.03	473
Kobble Creek	RR[1578]	0.20	0.56	1.40	488
Kogan	RR[1579]	0.30	0.77	2.09	146
Kokotungo	RR[1580]	0.33	0.92	2.62	78
Kolonga	RR[1581]	0.27	0.85	2.43	36
Koombooloomba	RR[1582]	0.40	1.18	3.23	0
Koongal	RR[1583]	0.56	1.04	1.78	3310
Kooralbyn	RR[1584]	0.16	0.44	1.02	1406
Kooralgin	RR[1585]	0.24	0.83	2.43	43
Kooringal (Qld)	RR[1586]	0.00	0.00	0.00	45
Kooroomool	RR[1587]	0.43	1.17	3.44	0
Kooroongarra	RR[1588]	0.29	0.84	2.41	51
Koumala	RR[1589]	0.47	1.13	2.56	663
Kowanyama	RR[1590]	0.64	1.51	3.26	690
Kowguran	RR[1591]	0.31	0.91	2.78	34
Kragra	RR[1592]	0.28	0.94	2.85	27
Kulangoor	RR[1593]	0.22	0.66	1.72	413
Kulgun	RR[1594]	0.23	0.71	2.14	53
Kullogum	RR[1595]	0.37	1.00	2.88	90
Kulpi	RR[1596]	0.27	0.76	2.19	68
Kuluin	RR[1597]	0.49	0.99	1.85	1871
Kumbarilla	RR[1598]	0.25	0.74	2.10	171
Kumbia	RR[1599]	0.35	0.99	2.47	246

Kunda Park	RR[1600]	0.23	0.73	2.03	27
Kunioon	RR[1601]	0.31	0.85	2.61	19
Kunwarara	RR[1602]	0.42	1.20	3.45	64
Kuraby	RR[1603]	0.12	0.24	0.49	6149
Kuranda	RR[1604]	**1.25**	**2.10**	**3.33**	2425
Kureelpa	RR[1605]	0.23	0.59	1.46	766
Kureen	RR[1606]	0.35	1.00	3.09	99
Kuridala	RR[1607]	0.50	1.57	4.75	10
Kurrimine Beach	RR[1608]	0.49	1.18	2.71	636
Kurrowah	RR[1609]	0.27	0.81	2.36	25
Kurumbul	RR[1610]	0.31	1.03	3.39	34
Kurwongbah	RR[1611]	0.22	0.55	1.30	1148
Kuttabul	RR[1612]	0.58	1.37	3.10	602
Kybong	RR[1613]	0.29	0.79	2.05	297
Kynuna	RR[1614]	0.50	1.52	4.28	42
Kyoomba	RR[1615]	0.40	1.17	3.76	76
Labrador	RR[1616]	0.71	0.97	1.29	15783
Laceys Creek	RR[1617]	0.23	0.61	1.65	223
Laglan	RR[1618]	0.39	1.06	3.05	3
Lagoon Pocket	RR[1619]	0.22	0.67	2.04	98
Laguna Quays	RR[1620]	0.32	1.04	3.79	65
Laidley	RR[1621]	0.62	1.12	1.95	3064
Laidley Creek West	RR[1622]	0.22	0.65	1.79	123
Laidley Heights	RR[1623]	0.20	0.48	1.15	1014
Laidley North	RR[1624]	0.23	0.60	1.64	323
Laidley South	RR[1625]	0.28	0.82	2.43	221
Lake Barrine	RR[1626]	0.46	1.27	3.56	128
Lake Borumba	RR[1627]	0.30	0.84	2.49	6
Lake Clarendon	RR[1628]	0.24	0.64	1.57	254
Lake Eacham	RR[1629]	0.37	0.90	2.34	364
Lake Macdonald	RR[1630]	0.48	1.08	2.21	1081
Lake Manchester	RR[1631]	0.27	0.78	2.12	16
Lake Mary	RR[1632]	0.31	0.92	2.60	83
Lake Monduran	RR[1633]	0.30	0.83	2.59	3
Lake Proserpine	RR[1634]	0.36	1.01	2.75	6
Lake Tinaroo	RR[1635]	0.37	1.10	3.28	0
Lake Wivenhoe	RR[1636]	0.30	0.84	2.46	3
Lakefield	RR[1637]	0.42	1.40	4.51	16
Lakeland	RR[1638]	0.41	1.12	2.86	261
Lakes Creek	RR[1639]	0.32	0.81	2.03	416
Lakeside	RR[1640]	0.32	0.90	2.61	49
Lamb Island	RR[1641]	0.47	1.39	3.78	382
Lamb Range	RR[1642]	0.41	1.11	3.13	0
Lamington (Qld)	RR[1643]	0.29	0.97	2.86	77
Lammermoor	RR[1644]	0.42	0.91	1.86	1674
Landers Shoot	RR[1645]	0.23	0.64	1.76	206
Landsborough (Qld)	RR[1646]	0.40	0.80	1.40	3114
Lanefield	RR[1647]	0.25	0.68	1.95	86
Langlands	RR[1648]	0.27	0.82	2.66	7
Langlo	RR[1649]	0.36	1.14	3.54	43

Langshaw	RR[1650]	0.25	0.70	2.05	143
Lannercost	RR[1651]	0.42	1.16	3.11	95
Lansdowne (Qld)	RR[1652]	0.40	1.15	3.44	11
Lanskey	RR[1653]	0.66	2.01	6.02	60
Larapinta (Qld)	RR[1654]	0.19	0.59	1.76	0
Laravale	RR[1655]	0.23	0.68	1.94	155
Lark Hill	RR[1656]	0.25	0.75	2.20	49
Laura (Qld)	RR[1657]	0.47	1.16	3.07	181
Lavelle	RR[1658]	0.28	0.82	2.53	19
Lawes	RR[1659]	0.23	0.59	1.61	319
Lawgi Dawes	RR[1660]	0.26	0.77	2.36	114
Lawn Hill	RR[1661]	0.67	2.09	6.18	15
Lawnton	RR[1662]	1.00	1.57	2.32	4606
Leafdale	RR[1663]	0.28	0.85	2.47	22
Lefthand Branch	RR[1664]	0.23	0.69	1.94	72
Leichhardt (Qld)	RR[1665]	0.43	0.81	1.40	2905
Lemontree	RR[1666]	0.27	0.78	2.17	34
Leslie	RR[1667]	0.30	0.84	2.65	9
Leslie Dam	RR[1668]	0.28	0.75	2.05	120
Lethebrook	RR[1669]	0.31	0.99	3.34	47
Leyburn	RR[1670]	0.50	1.28	3.07	407
Leydens Hill	RR[1671]	0.44	1.23	3.68	3
Lilydale (Qld)	RR[1672]	0.22	0.68	2.02	62
Lilyvale (Central Highlands - Qld)	RR[1673]	0.24	0.77	2.21	30
Lilyvale (Toowoomba - Qld)	RR[1674]	0.22	0.67	1.97	45
Limestone (Qld)	RR[1675]	0.39	1.21	3.45	7
Limestone Creek	RR[1676]	0.30	0.87	2.26	155
Limestone Ridges	RR[1677]	0.21	0.63	1.86	102
Limevale	RR[1678]	0.33	0.96	2.98	48
Linden (Qld)	RR[1679]	0.43	1.08	3.05	20
Linthorpe	RR[1680]	0.25	0.63	1.61	349
Linville	RR[1681]	0.27	0.76	2.13	138
Little Mountain	RR[1682]	0.44	0.71	1.09	8041
Little Mulgrave	RR[1683]	0.77	2.02	5.31	208
Lizard	RR[1684]	0.24	1.13	4.26	49
Llanarth (Qld)	RR[1685]	0.31	1.04	2.84	32
Loch Lomond	RR[1686]	0.22	0.72	2.11	90
Lochington	RR[1687]	0.34	0.96	2.60	36
Lockhart River	RR[1688]	0.87	2.16	4.74	499
Lockrose	RR[1689]	0.36	0.95	2.22	444
Lockyer (Qld)	RR[1690]	0.24	0.68	1.97	71
Lockyer Waters	RR[1691]	0.47	1.23	3.00	466
Logan Central	RR[1692]	**1.07**	**1.64**	**2.43**	4548
Logan Reserve	RR[1693]	0.20	0.46	0.98	2660
Logan Village	RR[1694]	0.47	0.86	1.43	3497
Loganholme	RR[1695]	0.42	0.74	1.21	4834
Loganlea	RR[1696]	0.25	0.48	0.80	5602
Lonesome Creek	RR[1697]	0.30	0.90	2.31	130
Long Flat (Qld)	RR[1698]	0.24	0.71	2.03	55
Long Pocket	RR[1699]	0.51	1.40	3.72	146

Longreach (Qld)	RR[1700]	**1.41**	**2.25**	**3.51**	2400
Lota	RR[1701]	0.32	0.67	1.31	2584
Lotus Creek	RR[1702]	0.31	0.89	2.52	61
Low Isles	RR[1703]	0.41	1.65	6.54	0
Lower Beechmont	RR[1704]	0.25	0.64	1.50	863
Lower Cowley	RR[1705]	0.40	1.18	3.52	44
Lower Cressbrook	RR[1706]	0.28	0.90	2.59	13
Lower Daintree	RR[1707]	0.39	1.22	3.86	81
Lower Mount Walker	RR[1708]	0.23	0.64	1.81	149
Lower Tenthill	RR[1709]	0.23	0.62	1.77	209
Lower Tully	RR[1710]	0.38	1.09	3.32	59
Lower Wonga	RR[1711]	0.35	0.92	2.45	272
Lowesby	RR[1712]	0.33	0.98	3.02	7
Lowestoff	RR[1713]	0.25	0.78	2.51	29
Lowmead	RR[1714]	0.36	0.99	2.71	136
Lowood	RR[1715]	0.76	1.30	2.19	3131
Lucinda	RR[1716]	0.50	1.41	3.49	380
Lumeah (Qld)	RR[1717]	0.38	1.24	3.58	3
Lumholtz	RR[1718]	0.41	1.24	3.56	0
Lundavra	RR[1719]	0.29	0.85	2.40	76
Luscombe	RR[1720]	0.27	0.75	1.99	236
Lutwyche	RR[1721]	**1.10**	**1.79**	**2.80**	3035
Lynam	RR[1722]	0.29	0.95	2.69	7
Lyndhurst (Qld)	RR[1723]	0.45	1.34	3.69	8
Lyndside	RR[1724]	0.44	1.34	3.63	0
Lyons (Qld)	RR[1725]	0.22	0.62	1.75	26
Lyra	RR[1726]	0.28	1.00	3.20	40
Lytton	RR[1727]	0.35	1.04	3.29	6
Ma Ma Creek	RR[1728]	0.29	0.85	2.39	129
Maadi	RR[1729]	0.52	1.47	4.30	44
Maalan	RR[1730]	0.38	1.16	3.04	22
Maaroom	RR[1731]	0.34	1.03	2.91	215
Mabuiag Island	RR[1732]	0.28	1.17	4.26	128
Macalister	RR[1733]	0.31	0.78	2.25	119
Macalister Range	RR[1734]	0.41	1.05	2.93	0
Macfarlane	RR[1735]	0.36	1.19	3.43	14
Macgregor (Qld)	RR[1736]	0.33	0.61	1.03	5155
Machans Beach	RR[1737]	**1.09**	**2.23**	**4.13**	886
Machine Creek	RR[1738]	0.30	0.86	2.60	98
Mackay	RR[1739]	**2.91**	**4.15**	**5.66**	3121
Mackay Harbour	RR[1740]	0.20	0.64	1.72	495
Mackenzie (Brisbane - Qld)	RR[1741]	0.17	0.42	0.96	1605
Mackenzie (Central Highlands - Qld	RR[1742]	0.32	0.91	2.78	26
Mackenzie River	RR[1743]	0.30	0.86	2.25	54
Macknade	RR[1744]	0.76	2.01	5.09	191
Maclagan	RR[1745]	0.25	0.77	2.09	155
Macleay Island	RR[1746]	**1.88**	**2.98**	**4.47**	2410
Magnolia	RR[1747]	0.26	0.82	2.36	100
Maidenhead	RR[1748]	0.27	0.93	3.19	11
Maidenwell	RR[1749]	0.36	1.02	2.60	182

Main Beach	RR[1750]	0.43	0.84	1.49	3588
Majors Creek (Qld)	RR[1751]	0.35	0.92	2.41	274
Malanda	RR[1752]	**1.33**	**2.32**	**3.78**	1666
Malarga	RR[1753]	0.31	0.84	2.47	17
Maleny	RR[1754]	0.55	1.00	1.68	3239
Malling	RR[1755]	0.25	0.81	2.71	24
Malmoe	RR[1756]	0.30	0.87	2.81	30
Malpas-Trenton	RR[1757]	0.49	1.54	4.00	8
Malu	RR[1758]	0.25	0.80	2.57	15
Mamu	RR[1759]	0.41	1.14	3.24	0
Manapouri	RR[1760]	0.25	0.75	2.13	41
Mandalay	RR[1761]	0.23	0.73	1.98	277
Maneroo	RR[1762]	0.62	1.79	6.06	31
Mango Hill	RR[1763]	0.16	0.33	0.61	6148
Manly (Qld)	RR[1764]	**1.34**	**2.06**	**3.10**	3352
Manly West	RR[1765]	0.59	0.88	1.24	9499
Mannuem	RR[1766]	0.32	0.86	2.67	71
Manoora (Qld)	RR[1767]	0.89	1.40	2.06	4710
Mansfield (Qld)	RR[1768]	0.44	0.70	1.14	6916
Mantuan Downs	RR[1769]	0.35	0.99	2.83	28
Manumbar	RR[1770]	0.29	0.81	2.21	40
Manunda	RR[1771]	**1.55**	**2.21**	**3.14**	4408
Manyung	RR[1772]	0.28	0.78	2.50	55
Mapleton	RR[1773]	0.42	0.94	1.86	1380
Mapoon	RR[1774]	0.28	0.86	2.59	232
Maramie	RR[1775]	0.47	1.34	4.09	9
Marburg	RR[1776]	0.40	0.98	2.33	698
Marcoola	RR[1777]	0.29	0.60	1.23	2641
Marcus Beach	RR[1778]	0.37	0.89	2.22	631
Mareeba	RR[1779]	**1.03**	**1.45**	**1.97**	8932
Margate (Qld)	RR[1780]	**1.03**	**1.57**	**2.28**	6054
Maria Creeks	RR[1781]	0.38	1.05	3.11	96
Marian	RR[1782]	0.23	0.50	1.00	2793
Marlborough	RR[1783]	0.31	0.93	2.65	125
Marmadua	RR[1784]	0.33	0.84	2.43	6
Marmor	RR[1785]	0.31	0.90	2.49	171
Marodian	RR[1786]	0.31	0.91	2.56	0
Maroochy River	RR[1787]	0.23	0.57	1.29	1222
Maroochydore	RR[1788]	0.78	1.07	1.41	14990
Maroon	RR[1789]	0.23	0.68	1.92	130
Maroondan	RR[1790]	0.36	0.93	2.37	287
Marsden	RR[1791]	0.57	0.85	1.22	9995
Marshlands	RR[1792]	0.30	0.87	2.56	10
Martyville	RR[1793]	0.45	1.24	3.53	95
Maryborough (Qld)	RR[1794]	**1.13**	**1.50**	**1.97**	12680
Maryborough West	RR[1795]	0.32	0.84	2.03	462
Marys Creek	RR[1796]	0.24	0.75	2.22	58
Maryvale (Livingstone - Qld)	RR[1797]	0.29	0.89	2.72	25
Maryvale (Southern Downs - Qld)	RR[1798]	0.23	0.65	1.71	258
Masig Island	RR[1799]	0.13	0.95	4.64	174

Massie	RR[1800]	0.28	0.78	2.15	82
Maudsland	RR[1801]	0.21	0.44	0.82	3989
Maxwelton	RR[1802]	0.50	1.45	4.15	19
May Downs	RR[1803]	0.27	0.81	2.38	75
McCutcheon	RR[1804]	0.38	1.17	3.70	19
McDesme	RR[1805]	0.34	0.98	2.62	219
McDowall	RR[1806]	0.39	0.70	1.12	5732
McEwens Beach	RR[1807]	0.34	1.01	2.83	172
McIlwraith	RR[1808]	0.27	0.77	2.15	155
McIntosh Creek	RR[1809]	0.25	0.70	2.18	93
McKinlay	RR[1810]	0.48	1.33	3.40	164
Meadowbrook	RR[1811]	0.25	0.54	1.09	2483
Meadowvale	RR[1812]	0.27	0.74	1.86	382
Meandarra	RR[1813]	0.30	0.81	2.06	213
Meikleville Hill	RR[1814]	0.31	0.98	2.75	390
Melawondi	RR[1815]	0.27	0.78	2.51	23
Meldale	RR[1816]	0.24	0.83	2.43	214
Melrose (Qld)	RR[1817]	0.30	0.93	2.67	0
Memerambi	RR[1818]	0.27	0.75	2.23	201
Mena Creek	RR[1819]	0.39	1.05	2.75	194
Mentmore	RR[1820]	0.35	1.05	3.37	0
Menzies (Qld)	RR[1821]	0.87	1.94	3.80	780
Mer Island	RR[1822]	0.30	1.55	5.02	318
Meridan Plains	RR[1823]	0.19	0.42	0.83	2802
Meringandan	RR[1824]	0.24	0.60	1.48	326
Meringandan West	RR[1825]	0.22	0.50	1.09	1560
Merlwood	RR[1826]	0.26	0.80	2.16	69
Mermaid Beach	RR[1827]	0.65	1.08	1.62	5870
Mermaid Waters	RR[1828]	0.99	1.39	1.82	9993
Merrimac	RR[1829]	0.28	0.54	0.92	5742
Merritts Creek	RR[1830]	0.26	0.73	2.08	65
Merryburn	RR[1831]	0.33	1.01	2.88	142
Merryvale	RR[1832]	0.21	0.65	2.35	16
Mexico	RR[1833]	0.38	1.15	3.30	19
Mia Mia (Qld)	RR[1834]	0.27	0.78	2.19	90
Miallo	RR[1835]	0.51	1.35	3.11	301
Miami	RR[1836]	0.90	1.37	2.01	5929
Miara	RR[1837]	0.27	0.83	2.38	38
Mica Creek	RR[1838]	0.44	1.26	3.52	150
Middle Park (Qld)	RR[1839]	0.28	0.57	1.08	3212
Middle Ridge	RR[1840]	0.27	0.49	0.86	5614
Middlebrook	RR[1841]	0.39	1.11	3.18	50
Middlemount	RR[1842]	0.24	0.59	1.31	1334
Middleton (Qld)	RR[1843]	0.53	1.45	4.13	6
Midge Point	RR[1844]	0.78	2.00	4.48	379
Midgee (Qld)	RR[1845]	0.42	1.10	2.92	41
Midgenoo	RR[1846]	0.38	1.11	3.69	37
Midgeree Bar	RR[1847]	0.41	1.18	3.56	39
Mighell	RR[1848]	0.50	1.39	3.42	323
Milbong	RR[1849]	0.22	0.62	1.75	128

Miles	RR[1850]	0.72	1.46	2.80	1341
Miles End	RR[1851]	0.78	2.09	5.27	194
Milford	RR[1852]	0.31	0.88	2.41	165
Millaa Millaa	RR[1853]	0.65	1.57	3.41	432
Millaroo	RR[1854]	0.34	1.00	2.81	77
Millbank (Qld)	RR[1855]	0.51	0.98	1.89	2107
Millchester	RR[1856]	0.51	1.37	3.38	413
Millmerran	RR[1857]	0.46	0.99	2.06	1249
Millmerran Downs	RR[1858]	0.26	0.77	2.37	86
Millmerran Woods	RR[1859]	0.25	0.80	2.32	82
Millstream (Qld)	RR[1860]	0.28	0.69	1.64	1012
Millwood	RR[1861]	0.28	0.82	2.44	15
Milman	RR[1862]	0.32	0.90	2.77	92
Milora	RR[1863]	0.21	0.60	1.59	96
Milton (Qld)	RR[1864]	0.52	1.08	2.01	2041
Mimosa (Qld)	RR[1865]	0.32	0.91	2.63	26
Min Min	RR[1866]	0.52	1.51	4.52	0
Minbun	RR[1867]	0.36	1.05	3.05	55
Minden	RR[1868]	0.31	0.76	1.70	921
Minerva	RR[1869]	0.31	0.91	2.62	40
Mingo	RR[1870]	0.30	0.89	2.68	8
Mingoola (Qld)	RR[1871]	0.29	0.95	3.17	13
Minnamoolka	RR[1872]	0.43	1.22	3.47	24
Minnie Downs	RR[1873]	0.36	1.15	3.29	13
Minyama	RR[1874]	0.39	0.82	1.58	2236
Mirani	RR[1875]	0.45	0.98	2.03	1185
Miriam Vale	RR[1876]	0.39	1.06	2.87	425
Mirriwinni	RR[1877]	0.55	1.43	3.26	369
Missen Flat	RR[1878]	0.26	0.81	2.36	14
Mission Beach	RR[1879]	0.63	1.61	3.51	677
Mission River	RR[1880]	0.50	1.23	2.76	695
Mistake Creek	RR[1881]	0.36	1.00	3.02	26
Mitchell (Qld)	RR[1882]	**1.08**	**2.32**	**4.37**	828
Mitchelton	RR[1883]	0.59	0.92	1.39	6755
Miva	RR[1884]	0.30	0.87	2.65	41
Moa Island	RR[1885]	0.50	1.70	4.92	316
Moffat Beach	RR[1886]	0.36	0.76	1.55	2131
Moffatdale	RR[1887]	0.30	0.79	2.13	139
Moggill	RR[1888]	0.31	0.61	1.09	3444
Molangul	RR[1889]	0.28	0.84	2.34	10
Molendinar	RR[1890]	0.34	0.64	1.11	5238
Mon Repos	RR[1891]	0.28	1.02	3.38	30
Mona Mona	RR[1892]	0.37	1.14	3.03	24
Mona Park	RR[1893]	0.35	1.03	2.74	91
Monal	RR[1894]	0.28	0.81	2.38	35
Monduran	RR[1895]	0.29	0.81	2.06	77
Mondure	RR[1896]	0.26	0.81	2.42	77
Monkland	RR[1897]	0.32	0.77	1.69	897
Monogorilby	RR[1898]	0.31	0.90	2.45	33
Mons	RR[1899]	0.30	0.79	1.91	756

Monsildale	RR[1900]	0.28	0.81	2.47	15
Monto	RR[1901]	0.58	1.27	2.57	995
Montrose (Southern Downs - Qld)	RR[1902]	0.29	0.84	2.38	0
Montrose (Western Downs - Qld)	RR[1903]	0.32	0.86	2.61	53
Montville	RR[1904]	0.33	0.78	1.90	848
Moodlu	RR[1905]	0.22	0.65	1.78	266
Mooga	RR[1906]	0.33	0.98	3.07	17
Moogerah	RR[1907]	0.23	0.65	1.62	187
Moola	RR[1908]	0.27	0.76	2.11	101
Moolboolaman	RR[1909]	0.32	0.88	2.16	361
Mooloo	RR[1910]	0.23	0.72	2.03	121
Mooloolaba	RR[1911]	0.85	1.28	1.85	6815
Mooloolah Valley	RR[1912]	0.18	0.40	0.83	2585
Moombra	RR[1913]	0.31	0.90	2.62	9
Moombria	RR[1914]	0.41	1.29	3.80	0
Moomin	RR[1915]	0.62	1.68	4.64	110
Moondooner	RR[1916]	0.26	0.82	2.38	49
Moonford	RR[1917]	0.25	0.80	2.25	124
Moongan	RR[1918]	0.35	1.12	3.19	101
Moonie	RR[1919]	0.29	0.81	2.22	140
Moorang	RR[1920]	0.22	0.69	1.97	40
Moore	RR[1921]	0.35	0.96	2.30	244
Moore Park Beach	RR[1922]	0.50	0.97	1.81	2134
Moores Pocket	RR[1923]	0.39	1.08	2.49	640
Moorina (Qld)	RR[1924]	0.22	0.57	1.47	332
Moorland (Qld)	RR[1925]	0.25	0.76	1.98	236
Mooroobool	RR[1926]	0.68	1.09	1.67	5746
Moorooka	RR[1927]	0.98	1.44	1.95	8587
Moraby	RR[1928]	0.31	0.94	2.67	34
Moranbah	RR[1929]	0.21	0.42	0.73	6234
Morayfield	RR[1930]	0.32	0.49	0.73	16455
Moregatta	RR[1931]	0.37	1.12	3.17	9
Morella	RR[1932]	0.51	1.47	3.89	25
Moresby (Qld)	RR[1933]	0.87	2.30	5.78	114
Moreton Bay	RR[1934]	0.00	0.00	21.84	0
Moreton Island	RR[1935]	0.00	0.00	0.00	227
Morgan Park	RR[1936]	0.24	0.76	2.15	72
Morganville	RR[1937]	0.25	0.85	2.40	97
Morinish	RR[1938]	0.32	0.93	2.72	74
Morinish South	RR[1939]	0.35	1.03	2.99	5
Morningside	RR[1940]	0.63	0.96	1.34	8804
Mornington (Qld)	RR[1941]	0.67	1.40	2.56	1393
Morton Vale	RR[1942]	0.31	0.91	2.65	124
Morven (Qld)	RR[1943]	0.34	1.00	2.62	174
Morwincha	RR[1944]	0.22	0.68	2.10	24
Mosman Park (Qld)	RR[1945]	0.45	1.22	2.92	296
Mosquito Creek	RR[1946]	0.30	0.89	2.47	13
Mossman	RR[1947]	**1.07**	**2.02**	**3.57**	1553
Mossman Gorge	RR[1948]	0.71	1.89	4.85	193
Mothar Mountain	RR[1949]	0.23	0.61	1.57	448

Motley	RR[1950]	0.26	0.79	2.24	9
Mount Abundance	RR[1951]	0.33	0.90	2.71	146
Mount Alford	RR[1952]	0.22	0.61	1.68	231
Mount Alma	RR[1953]	0.31	0.88	2.41	38
Mount Archer (Rockhampton - Qld)	RR[1954]	0.53	1.52	4.44	81
Mount Archer (Somerset - Qld)	RR[1955]	0.24	0.66	1.80	257
Mount Barney	RR[1956]	0.25	0.73	2.21	18
Mount Beppo	RR[1957]	0.34	0.98	2.74	182
Mount Berryman	RR[1958]	0.23	0.67	1.98	86
Mount Bindango	RR[1959]	0.36	1.00	3.07	22
Mount Binga	RR[1960]	0.28	0.82	2.24	51
Mount Britton	RR[1961]	0.29	0.86	2.40	9
Mount Byron	RR[1962]	0.25	0.75	2.13	18
Mount Carbine	RR[1963]	0.54	1.48	3.92	98
Mount Chalmers	RR[1964]	0.27	0.89	2.25	192
Mount Charlton	RR[1965]	0.53	1.47	3.63	134
Mount Colliery	RR[1966]	0.24	0.67	2.03	98
Mount Coolon	RR[1967]	0.35	0.94	2.67	54
Mount Coolum	RR[1968]	0.16	0.34	0.72	3433
Mount Coot-tha	RR[1969]	0.23	0.66	1.98	0
Mount Cotton	RR[1970]	0.28	0.54	0.96	4989
Mount Crosby	RR[1971]	0.46	1.03	2.13	1370
Mount Darry	RR[1972]	0.27	0.77	2.27	35
Mount Debateable	RR[1973]	0.29	0.89	2.90	46
Mount Delaney	RR[1974]	0.26	0.74	2.20	65
Mount Edwards	RR[1975]	0.24	0.67	1.89	55
Mount Elliot (Qld)	RR[1976]	0.41	1.12	3.28	8
Mount Emlyn	RR[1977]	0.25	0.83	2.66	9
Mount Enniskillen	RR[1978]	0.39	1.13	3.54	13
Mount Forbes	RR[1979]	0.21	0.59	1.69	198
Mount Fox	RR[1980]	0.52	1.46	4.10	71
Mount French	RR[1981]	0.26	0.72	2.07	80
Mount Gardiner	RR[1982]	0.33	0.92	2.65	28
Mount Garnet	RR[1983]	0.40	1.12	2.92	372
Mount Gipps	RR[1984]	0.23	0.78	2.65	7
Mount Glorious	RR[1985]	0.39	1.02	2.52	254
Mount Gravatt	RR[1986]	0.84	1.48	2.35	2793
Mount Gravatt East	RR[1987]	0.52	0.80	1.14	9652
Mount Hallen	RR[1988]	0.36	0.90	2.37	366
Mount Howe	RR[1989]	0.36	1.13	3.24	3
Mount Hutton (Qld)	RR[1990]	0.38	1.15	3.33	28
Mount Irving	RR[1991]	0.23	0.75	2.21	15
Mount Isa	RR[1992]	**5.31**	**11.62**	**23.95**	95
Mount Isa City	RR[1993]	0.59	1.77	4.78	81
Mount Jukes	RR[1994]	0.24	0.71	1.97	312
Mount Julian	RR[1995]	0.35	0.93	2.46	388
Mount Kelly	RR[1996]	0.39	1.15	2.94	228
Mount Kilcoy	RR[1997]	0.24	0.73	2.01	214
Mount Kynoch	RR[1998]	0.23	0.67	1.98	176
Mount Larcom	RR[1999]	0.75	1.93	4.71	282

Mount Lawless	RR[2000]	0.29	0.92	3.03	9
Mount Lindesay (Qld)	RR[2001]	0.20	0.74	2.64	0
Mount Lofty	RR[2002]	0.53	0.98	1.68	3073
Mount Louisa	RR[2003]	0.34	0.61	0.99	6689
Mount Low	RR[2004]	0.31	0.64	1.17	3352
Mount Luke	RR[2005]	0.25	0.75	2.25	31
Mount Macarthur	RR[2006]	0.26	0.77	2.57	13
Mount Mackay	RR[2007]	0.36	1.11	3.48	0
Mount Maria	RR[2008]	0.24	0.75	2.24	163
Mount Marlow	RR[2009]	0.47	1.40	3.80	105
Mount Marrow	RR[2010]	0.24	0.69	1.80	142
Mount Marshall	RR[2011]	0.42	1.18	3.11	200
Mount Martin	RR[2012]	0.28	0.82	2.23	158
Mount Mceuen	RR[2013]	0.29	0.86	2.52	33
Mount Mee	RR[2014]	0.22	0.60	1.55	406
Mount Mellum	RR[2015]	0.28	0.75	2.03	380
Mount Moffatt	RR[2016]	0.38	1.09	3.24	0
Mount Molar	RR[2017]	0.26	0.79	2.29	92
Mount Molloy	RR[2018]	0.59	1.57	4.32	215
Mount Morgan	RR[2019]	**1.81**	**3.03**	**4.73**	1656
Mount Moriah	RR[2020]	0.25	0.76	2.25	10
Mount Mort	RR[2021]	0.22	0.65	1.85	82
Mount Mulgrave	RR[2022]	0.43	1.28	3.82	0
Mount Mulligan	RR[2023]	0.42	1.25	3.40	4
Mount Murchison	RR[2024]	0.27	0.81	2.43	112
Mount Nathan	RR[2025]	0.42	1.04	2.22	952
Mount Nebo	RR[2026]	0.23	0.65	1.53	359
Mount Ommaney	RR[2027]	0.34	0.75	1.52	2011
Mount Ossa	RR[2028]	0.30	0.85	2.48	178
Mount Pelion	RR[2029]	0.29	0.89	2.67	106
Mount Perry	RR[2030]	0.33	0.87	2.14	441
Mount Peter	RR[2031]	0.40	1.07	3.02	71
Mount Pleasant (Mackay - Qld)	RR[2032]	0.42	0.81	1.43	3669
Mount Pleasant (Moreton Bay - Qlc	RR[2033]	0.23	0.61	1.52	261
Mount Pluto	RR[2034]	0.29	1.00	3.03	3
Mount Rascal	RR[2035]	0.22	0.58	1.59	318
Mount Rooper	RR[2036]	0.21	0.79	2.62	0
Mount Samson	RR[2037]	0.27	0.68	1.64	455
Mount Sheridan (Qld)	RR[2038]	0.40	0.69	1.15	6327
Mount St John	RR[2039]	0.35	1.00	2.90	62
Mount Stanley	RR[2040]	0.27	0.86	2.31	4
Mount Steadman	RR[2041]	0.31	0.93	2.86	8
Mount Stuart (Qld)	RR[2042]	0.33	0.91	2.56	0
Mount Sturt	RR[2043]	0.23	0.72	2.25	13
Mount Surprise	RR[2044]	0.54	1.47	3.59	146
Mount Surround	RR[2045]	0.39	1.08	2.77	132
Mount Sylvia	RR[2046]	0.25	0.67	1.85	81
Mount Tabor	RR[2047]	0.27	0.72	2.07	60
Mount Tarampa	RR[2048]	0.24	0.68	1.69	365
Mount Tom	RR[2049]	0.27	0.83	2.52	39

Mount Tully	RR[2050]	0.29	0.84	2.51	99
Mount Tyson	RR[2051]	0.28	0.84	2.13	222
Mount Urah	RR[2052]	0.33	0.93	2.69	21
Mount Walker (Qld)	RR[2053]	0.23	0.62	1.78	104
Mount Walker West	RR[2054]	0.23	0.68	2.13	19
Mount Warren Park	RR[2055]	0.53	0.90	1.44	4582
Mount Whitestone	RR[2056]	0.30	0.82	2.26	111
Mount Wyatt	RR[2057]	0.36	1.02	2.73	44
Mountain Camp	RR[2058]	0.27	0.82	2.63	18
Mountain Creek (Qld)	RR[2059]	0.40	0.64	1.00	8579
Moura	RR[2060]	0.63	1.33	2.48	1378
Mourilyan	RR[2061]	0.82	2.06	4.40	455
Mourilyan Harbour	RR[2062]	0.39	1.18	3.33	125
Mowbray (Qld)	RR[2063]	0.35	0.96	2.57	261
Mowbullan	RR[2064]	0.21	0.85	3.14	11
Moy Pocket	RR[2065]	0.28	0.77	2.19	83
Mp Creek	RR[2066]	0.31	0.86	2.44	24
Muckadilla	RR[2067]	0.30	0.99	3.17	47
Mudgeeraba	RR[2068]	0.37	0.58	0.91	10478
Mudjimba	RR[2069]	0.31	0.70	1.33	2042
Mudlo	RR[2070]	0.31	0.86	2.68	2
Muirlea	RR[2071]	0.28	0.80	2.15	153
Mulambin	RR[2072]	0.30	0.85	2.00	680
Mulara	RR[2073]	0.57	1.56	4.41	73
Muldu	RR[2074]	0.26	0.80	2.59	0
Mulgildie	RR[2075]	0.26	0.77	2.04	138
Mulgowie	RR[2076]	0.23	0.70	1.80	142
Mulgrave (Qld)	RR[2077]	0.37	1.10	3.00	14
Mullett Creek	RR[2078]	0.27	0.80	2.53	71
Munbilla	RR[2079]	0.23	0.67	1.86	75
Munbura	RR[2080]	0.26	0.83	2.45	101
Munderra	RR[2081]	0.45	1.21	3.50	0
Mundingburra	RR[2082]	0.94	1.55	2.42	3017
Mundoo	RR[2083]	0.56	1.53	3.94	177
Mundoolun	RR[2084]	0.43	0.95	1.95	1153
Mundowran	RR[2085]	0.31	0.90	2.44	99
Mundubbera	RR[2086]	0.68	1.53	2.88	1035
Mungabunda	RR[2087]	0.33	0.94	2.76	53
Mungallala	RR[2088]	0.33	0.96	2.97	106
Mungallala South	RR[2089]	0.32	1.08	2.93	24
Mungalli	RR[2090]	0.39	1.18	3.35	38
Mungar	RR[2091]	0.39	1.21	3.09	248
Mungindi (Qld)	RR[2092]	0.27	0.86	2.50	108
Mungungo	RR[2093]	0.24	0.79	2.37	57
Mungy	RR[2094]	0.30	0.88	2.63	3
Muniganeen	RR[2095]	0.25	0.69	2.20	42
Munna Creek	RR[2096]	0.30	0.97	2.79	23
Munro Plains	RR[2097]	0.42	1.16	3.17	60
Munruben	RR[2098]	0.24	0.58	1.10	2311
Murarrie	RR[2099]	0.30	0.59	1.08	3442

Murgon	RR[2100]	0.44	0.91	1.74	1903
Murphys Creek (Qld)	RR[2101]	0.23	0.59	1.52	485
Murray	RR[2102]	0.20	0.51	1.12	1491
Murray Upper	RR[2103]	0.59	1.54	3.82	207
Murrays Bridge	RR[2104]	0.33	0.97	2.63	81
Murrigal	RR[2105]	0.36	1.08	3.43	61
Murrumba	RR[2106]	0.28	0.89	2.63	12
Murrumba Downs	RR[2107]	0.42	0.69	1.08	8286
Murweh	RR[2108]	0.37	1.13	3.99	0
Mutarnee	RR[2109]	0.32	1.06	2.91	101
Mutchilba	RR[2110]	0.58	1.36	3.22	394
Mutdapilly	RR[2111]	0.20	0.58	1.63	254
Muttaburra	RR[2112]	0.61	1.70	4.69	75
Myall Park (Qld)	RR[2113]	0.28	0.93	2.82	14
Myrtlevale	RR[2114]	0.25	0.81	2.53	125
Mysterton	RR[2115]	0.46	1.13	2.43	660
Nahrunda	RR[2116]	0.23	0.68	1.97	152
Nambour	RR[2117]	**1.12**	**1.57**	**2.10**	9098
Nanango	RR[2118]	**1.38**	**2.15**	**3.26**	2958
Nandi	RR[2119]	0.23	0.76	2.18	80
Nandowrie	RR[2120]	0.35	1.00	3.14	31
Nangram	RR[2121]	0.33	0.92	2.71	8
Nangwee	RR[2122]	0.25	0.74	2.14	38
Nankin	RR[2123]	0.33	0.96	2.72	134
Nanum	RR[2124]	0.29	0.84	2.05	670
Narangba	RR[2125]	0.26	0.41	0.63	13597
Narbethong (Qld)	RR[2126]	0.41	1.21	3.42	0
Narko	RR[2127]	0.26	0.84	2.48	18
Nathan	RR[2128]	0.22	0.52	1.24	1059
Natural Bridge	RR[2129]	0.22	0.67	2.05	92
Nearum	RR[2130]	0.30	0.86	2.56	4
Nebine	RR[2131]	0.38	1.07	3.14	19
Nebo	RR[2132]	0.24	0.64	1.59	596
Neerdie	RR[2133]	0.30	0.83	2.19	100
Nelly Bay	RR[2134]	**1.24**	**2.61**	**4.64**	1040
Nerada	RR[2135]	0.39	1.08	3.19	72
Nerang	RR[2136]	**1.06**	**1.40**	**1.83**	13787
Neranwood	RR[2137]	0.30	0.95	2.70	55
Nerimbera	RR[2138]	0.33	0.85	2.23	249
Netherby (Qld)	RR[2139]	0.32	0.94	2.96	35
Netherdale	RR[2140]	0.28	0.84	2.55	77
Neumgna	RR[2141]	0.30	0.87	2.50	2
Neurum	RR[2142]	0.25	0.73	1.98	128
Neusa Vale	RR[2143]	0.27	0.77	2.34	44
Nevilton	RR[2144]	0.26	0.77	2.16	30
New Auckland	RR[2145]	0.20	0.44	0.85	3841
New Beith	RR[2146]	0.17	0.38	0.76	2965
New Chum	RR[2147]	0.26	0.76	2.30	0
New Farm	RR[2148]	0.83	1.15	1.52	11457
New Harbourline	RR[2149]	0.40	1.13	2.98	162

New Mapoon	RR[2150]	0.20	0.73	2.47	249
New Moonta	RR[2151]	0.28	0.84	2.56	56
Newell	RR[2152]	0.67	1.69	3.85	283
Newlands (Qld)	RR[2153]	0.35	0.95	2.75	51
Newmarket	RR[2154]	0.65	1.10	1.77	4234
Newport (Qld)	RR[2155]	0.29	0.67	1.32	2520
Newstead (Qld)	RR[2156]	0.20	0.47	1.04	2101
Newtown (Ipswich - Qld)	RR[2157]	**4.12**	**6.30**	**8.95**	1258
Newtown (Toowoomba - Qld)	RR[2158]	0.10	0.23	0.44	7825
Ngatjan	RR[2159]	0.40	1.21	3.35	0
Nicholson (Qld)	RR[2160]	0.48	1.50	5.05	0
Nikenbah	RR[2161]	0.26	0.67	1.59	485
Nindaroo	RR[2162]	0.23	0.62	1.71	192
Ninderry	RR[2163]	0.31	0.80	1.81	878
Nindooinbah	RR[2164]	0.26	0.75	2.22	62
Nine Mile (Qld)	RR[2165]	0.37	0.99	2.81	57
Nine Mile Creek	RR[2166]	0.37	1.13	3.45	17
Ningi	RR[2167]	0.70	1.22	2.01	3625
Nive	RR[2168]	0.37	1.18	3.38	28
No 4 Branch	RR[2169]	0.42	1.15	3.15	51
No 5 Branch	RR[2170]	0.44	1.20	3.69	9
No 6 Branch	RR[2171]	0.45	1.20	3.62	44
Noah	RR[2172]	0.45	1.42	4.34	0
Nobby	RR[2173]	0.37	0.95	2.27	445
Nockatunga	RR[2174]	0.39	1.19	3.96	11
Nome	RR[2175]	0.50	1.20	2.54	888
Noorama	RR[2176]	0.35	1.07	3.38	21
Noorindoo	RR[2177]	0.32	0.93	2.87	46
Noosa Heads	RR[2178]	0.44	0.85	1.43	3920
Noosa North Shore	RR[2179]	0.28	0.78	2.03	156
Noosaville	RR[2180]	0.72	1.10	1.60	7042
Norley	RR[2181]	0.36	1.22	3.39	0
Norman Gardens	RR[2182]	0.24	0.43	0.75	7814
Norman Park	RR[2183]	0.58	0.98	1.62	5007
Normanton	RR[2184]	**2.40**	**4.18**	**7.01**	933
North Aramara	RR[2185]	0.33	0.95	2.84	28
North Arm (Qld)	RR[2186]	0.37	0.90	2.16	548
North Booval	RR[2187]	0.43	0.89	1.62	2384
North Branch (Southern Downs - Ql	RR[2188]	0.21	0.69	2.32	44
North Branch (Toowoomba - Qld)	RR[2189]	0.26	0.78	2.24	30
North Bungunya	RR[2190]	0.31	0.92	2.97	32
North Deep Creek	RR[2191]	0.44	1.11	2.53	325
North Eton	RR[2192]	0.33	0.96	2.56	152
North Gregory	RR[2193]	0.45	1.34	3.96	45
North Ipswich	RR[2194]	0.51	0.94	1.60	3592
North Isis	RR[2195]	0.48	1.28	2.83	476
North Johnstone	RR[2196]	0.37	1.08	3.02	70
North Lakes	RR[2197]	0.12	0.22	0.36	15725
North Mackay	RR[2198]	**1.16**	**1.70**	**2.48**	4943
North Maclagan	RR[2199]	0.23	0.84	2.74	5

North Maclean	RR[2200]	0.47	1.03	2.08	1262
North Maleny	RR[2201]	0.22	0.57	1.49	556
North Stradbroke Island	RR[2202]	0.73	2.35	6.72	117
North Talwood	RR[2203]	0.29	0.88	2.29	134
North Tivoli	RR[2204]	0.30	0.88	2.41	67
North Toowoomba	RR[2205]	0.70	1.29	2.29	2439
North Ward	RR[2206]	0.88	1.41	2.15	4404
Northgate (Qld)	RR[2207]	0.48	0.88	1.48	3811
Northhead	RR[2208]	0.50	1.40	3.70	0
Norville	RR[2209]	**1.23**	**2.06**	**3.36**	1988
Norwell	RR[2210]	0.22	0.63	1.78	166
Norwin	RR[2211]	0.25	0.76	2.27	64
Nudgee	RR[2212]	0.60	1.16	2.03	2872
Nudgee Beach	RR[2213]	0.37	1.02	2.70	234
Nukku	RR[2214]	0.30	0.85	2.54	18
Numinbah Valley	RR[2215]	0.23	0.63	1.76	206
Nundah	RR[2216]	0.49	0.74	1.08	10591
Nundubbermere	RR[2217]	0.38	1.15	3.58	74
Nutgrove	RR[2218]	0.29	0.84	2.42	25
Nychum	RR[2219]	0.44	1.23	3.77	0
Oak Beach	RR[2224]	0.54	1.84	5.33	171
Oak Valley	RR[2225]	0.63	1.50	3.57	387
Oakdale (Qld)	RR[2226]	0.26	0.79	2.47	48
Oakenden	RR[2227]	0.26	0.71	1.86	330
Oakey	RR[2228]	0.86	1.49	2.30	3695
Oakey Creek	RR[2229]	0.36	1.11	3.39	3
Oakhurst (Qld)	RR[2230]	0.24	0.63	1.46	1146
Oakview	RR[2231]	0.27	0.80	2.42	29
Oakwood (Qld)	RR[2232]	0.51	1.29	3.06	297
Oaky Creek	RR[2233]	0.24	0.75	2.18	70
Oberina	RR[2234]	0.33	0.94	3.03	9
Obi Obi	RR[2235]	0.35	0.98	2.62	162
O'Bil Bil	RR[2220]	0.30	0.94	2.59	29
O'Briens Hill	RR[2221]	0.42	1.20	3.61	10
Obum Obum	RR[2236]	0.24	0.65	2.03	104
Ocean View	RR[2237]	0.40	0.89	2.01	780
O'Connell (Qld)	RR[2222]	0.22	0.63	1.61	237
Ogmore	RR[2238]	0.33	0.91	2.64	83
Okeden	RR[2239]	0.30	0.96	2.82	21
Old Cooranga	RR[2240]	0.29	0.94	2.67	24
Old Talgai	RR[2241]	0.29	0.87	2.50	26
Oman Ama	RR[2242]	0.30	0.92	2.47	34
One Mile (Qld)	RR[2243]	0.47	1.02	1.93	1609
Oombabeer	RR[2244]	0.30	0.94	2.74	47
Oonoonba	RR[2245]	0.79	1.58	2.88	1419
Ooralea	RR[2246]	0.52	0.99	1.79	2693
Opalton	RR[2247]	0.49	1.39	3.92	58
Orallo	RR[2248]	0.38	1.04	3.02	22
Orange Creek	RR[2249]	0.29	0.79	2.19	150
Orange Hill	RR[2250]	0.28	0.88	2.64	155

O'Reilly	RR[2223]	0.26	0.78	2.53	0
Orient	RR[2251]	0.42	1.21	3.56	0
Orion	RR[2252]	0.31	0.89	2.56	61
Ormeau	RR[2253]	0.22	0.38	0.63	10468
Ormeau Hills	RR[2254]	0.16	0.38	0.82	2316
Ormiston	RR[2255]	0.53	0.90	1.47	4808
Osborne (Qld)	RR[2256]	0.35	0.94	2.74	205
Ottaba	RR[2257]	0.28	0.83	2.52	48
Owanyilla	RR[2258]	0.28	0.83	2.17	161
Owens Creek	RR[2259]	0.27	0.84	2.42	118
Oxenford	RR[2260]	0.45	0.72	1.09	9319
Oxford	RR[2261]	0.31	0.86	2.42	33
Oxley (Qld)	RR[2262]	0.63	1.00	1.50	6582
Oyster Creek	RR[2263]	0.26	0.85	2.47	40
Pacific Haven	RR[2264]	0.42	1.11	2.64	646
Pacific Heights	RR[2265]	0.25	0.67	1.57	732
Pacific Paradise	RR[2266]	0.81	1.55	2.71	1752
Pacific Pines	RR[2267]	0.33	0.55	0.81	12395
Packers Camp	RR[2268]	0.35	1.13	3.36	72
Paddington (Qld)	RR[2269]	0.42	0.70	1.11	7203
Paddys Green	RR[2270]	0.35	0.93	2.27	296
Paget	RR[2271]	0.55	1.54	3.91	221
Palen Creek	RR[2272]	0.24	0.66	1.86	258
Palgrave	RR[2273]	0.28	0.84	2.61	17
Pallara	RR[2274]	0.30	0.78	2.00	434
Pallarenda	RR[2275]	**1.20**	**2.61**	**5.38**	634
Palm Beach (Qld)	RR[2276]	0.83	1.14	1.54	12186
Palm Cove	RR[2277]	0.43	0.99	1.89	1799
Palm Grove (Qld)	RR[2278]	0.25	0.82	2.51	73
Palm Island	RR[2279]	0.34	0.87	1.84	1674
Palmer (Qld)	RR[2280]	0.44	1.31	4.15	0
Palmerston (Qld)	RR[2281]	0.43	1.15	3.56	3
Palmtree	RR[2282]	0.27	0.77	2.25	60
Palmview	RR[2283]	0.18	0.50	1.24	700
Palmwoods (Qld)	RR[2284]	0.40	0.74	1.27	4494
Palmyra (Qld)	RR[2285]	0.27	0.73	1.93	207
Paluma	RR[2286]	0.38	1.03	2.68	56
Pampas	RR[2287]	0.26	0.78	2.06	51
Paradise Point	RR[2288]	0.64	1.05	1.63	5698
Park Avenue	RR[2289]	0.99	1.55	2.34	4126
Park Ridge	RR[2290]	0.40	0.81	1.57	2200
Park Ridge South	RR[2291]	0.25	0.60	1.30	1461
Parkhurst	RR[2292]	0.32	0.67	1.32	1818
Parkinson	RR[2293]	0.19	0.34	0.62	8383
Parklands (Qld)	RR[2294]	0.25	0.73	1.90	177
Parknook	RR[2295]	0.28	0.92	2.91	48
Parkside (Qld)	RR[2296]	0.64	1.36	2.58	1204
Parkwood (Qld)	RR[2297]	0.35	0.60	1.00	7171
Parramatta Park	RR[2298]	**1.04**	**1.72**	**2.65**	3078
Parrearra	RR[2299]	0.56	1.01	1.64	3812

Pasha	RR[2300]	0.31	0.89	2.67	39
Passchendaele	RR[2301]	0.27	0.84	2.55	24
Paterson (Qld)	RR[2302]	0.53	1.45	4.09	114
Patrick	RR[2303]	0.41	1.21	3.71	26
Patrick Estate	RR[2304]	0.26	0.77	2.19	164
Pauls Pocket	RR[2305]	0.33	1.00	2.69	28
Peachester	RR[2306]	0.25	0.61	1.36	1093
Peacock Siding	RR[2307]	0.39	1.17	3.42	67
Peak Crossing	RR[2308]	0.30	0.74	1.69	731
Peak Vale	RR[2309]	0.33	0.95	2.70	28
Pechey	RR[2310]	0.27	0.74	2.13	94
Peel Island	RR[2311]	0.00	0.00	23.18	0
Peeramon	RR[2312]	0.39	0.98	2.27	501
Pelham (Qld)	RR[2313]	0.32	0.94	2.82	8
Pelican (Qld)	RR[2314]	0.27	0.83	2.58	14
Pelican Creek	RR[2315]	0.43	1.25	3.52	17
Pelican Waters	RR[2316]	0.38	0.67	1.14	5278
Pentland	RR[2317]	0.35	0.95	2.47	249
Penwhaupell	RR[2318]	0.30	0.91	2.63	0
Peranga	RR[2319]	0.36	1.04	2.94	66
Peregian Beach	RR[2320]	0.29	0.60	1.12	3035
Peregian Springs	RR[2321]	0.19	0.38	0.69	5148
Perseverance	RR[2322]	0.26	0.79	2.50	51
Perwillowen	RR[2323]	0.33	0.90	2.50	188
Petford	RR[2324]	0.42	1.19	3.55	32
Petrie	RR[2325]	0.75	1.14	1.72	6749
Petrie Terrace	RR[2326]	0.30	0.80	1.80	1035
Pheasant Creek (Qld)	RR[2327]	0.35	0.91	2.89	35
Philpott	RR[2328]	0.31	0.95	2.88	35
Pialba	RR[2329]	0.76	1.30	2.18	3098
Pickanjinnie	RR[2330]	0.32	0.89	2.46	33
Picnic Bay	RR[2331]	**1.60**	**4.19**	**9.67**	253
Pie Creek	RR[2332]	0.25	0.67	1.42	805
Pierces Creek	RR[2333]	0.28	0.82	2.47	62
Pikedale	RR[2334]	0.30	0.88	2.43	31
Pikes Creek	RR[2335]	0.32	0.96	2.65	22
Pile Gully	RR[2336]	0.31	0.94	2.66	3
Pilerwa	RR[2337]	0.27	0.87	2.79	41
Pilton	RR[2338]	0.26	0.73	2.23	74
Pimlico (Qld)	RR[2339]	**1.44**	**2.41**	**3.98**	2060
Pimpama	RR[2340]	0.24	0.45	0.78	6645
Pimpimbudgee	RR[2341]	0.27	0.83	2.41	53
Pin Gin Hill	RR[2342]	0.38	1.10	2.97	144
Pinbarren	RR[2343]	0.22	0.65	1.69	299
Pindi Pindi	RR[2344]	0.31	0.95	2.66	75
Pine Creek (Qld)	RR[2345]	0.37	1.05	2.63	192
Pine Hill (Qld)	RR[2346]	0.35	1.02	3.06	3
Pine Hills	RR[2347]	0.33	0.94	3.22	0
Pine Mountain (Qld)	RR[2348]	0.30	0.70	1.50	1289
Pinelands (Qld)	RR[2349]	0.26	0.80	2.34	74

Pinevale	RR[2350]	0.30	0.81	2.43	43
Pinjarra Hills	RR[2351]	0.21	0.56	1.44	533
Pink Lily	RR[2352]	0.43	1.18	2.94	196
Pinkenba	RR[2353]	0.70	1.92	4.52	341
Pinnacle (Qld)	RR[2354]	0.28	0.79	2.35	173
Pinnacles	RR[2355]	0.31	0.87	2.45	74
Pioneer (Qld)	RR[2356]	0.54	1.15	2.10	1514
Pioneers Rest	RR[2357]	0.30	0.88	2.76	52
Pirrinuan	RR[2358]	0.26	0.78	2.22	146
Pittsworth	RR[2359]	0.78	1.41	2.36	2611
Piturie	RR[2360]	0.50	1.52	4.56	42
Placid Hills	RR[2361]	0.25	0.66	1.67	669
Plainby	RR[2362]	0.28	0.82	2.35	33
Plainland	RR[2363]	0.22	0.55	1.22	1249
Pleystowe	RR[2364]	0.23	0.62	1.71	301
Point Arkwright	RR[2365]	0.18	0.59	1.71	234
Point Lookout	RR[2366]	**1.15**	**3.09**	**6.29**	635
Point Vernon	RR[2367]	0.78	1.24	1.92	4702
Pomona (Qld)	RR[2368]	0.27	0.62	1.29	2370
Pony Hills	RR[2369]	0.33	1.01	2.97	7
Poona	RR[2370]	0.28	0.85	2.34	459
Porcupine	RR[2371]	0.45	1.24	3.18	29
Pormpuraaw	RR[2372]	0.81	1.91	4.06	548
Port Alma	RR[2373]	0.35	1.08	3.08	0
Port Curtis	RR[2374]	0.38	0.96	2.47	234
Port Douglas	RR[2375]	0.25	0.55	1.07	3095
Port Of Brisbane	RR[2376]	0.25	1.02	4.53	0
Port Wine	RR[2377]	0.34	1.01	3.05	24
Portsmith	RR[2378]	0.37	0.99	2.67	256
Poruma Island	RR[2379]	0.19	0.97	4.28	122
Postmans Ridge	RR[2380]	0.35	0.96	2.49	324
Pozieres	RR[2381]	0.25	0.83	2.32	137
Prairie (Qld)	RR[2382]	0.42	1.21	3.25	115
Pratten	RR[2383]	0.38	0.97	2.72	171
Prawle	RR[2384]	0.32	0.89	2.51	25
Prenzlau	RR[2385]	0.26	0.75	2.04	354
Preston (Toowoomba - Qld)	RR[2386]	0.27	0.70	1.70	448
Preston (Whitsunday - Qld)	RR[2387]	0.29	0.79	2.32	233
Priestdale	RR[2388]	0.24	0.67	1.77	117
Prince Henry Heights	RR[2389]	0.22	0.60	1.66	466
Prince Of Wales	RR[2390]	0.20	0.89	4.13	91
Promisedland	RR[2391]	0.32	0.92	2.85	20
Proserpine	RR[2392]	0.81	1.42	2.33	2865
Prospect (Qld)	RR[2393]	0.27	0.75	1.96	207
Proston	RR[2394]	0.44	1.16	3.06	326
Pullenvale	RR[2395]	0.43	0.83	1.53	2441
Punchs Creek	RR[2396]	0.28	0.84	2.29	27
Punsand	RR[2397]	0.26	1.23	4.50	17
Purga	RR[2398]	0.28	0.71	1.68	472
Purrawunda	RR[2399]	0.24	0.74	2.33	6

Queenton	RR[2400]	0.59	1.36	2.79	983
Quilpie	RR[2401]	0.96	2.17	4.79	467
Quinalow	RR[2402]	0.26	0.77	2.17	120
Qunaba	RR[2403]	0.34	0.89	2.11	659
Racecourse	RR[2404]	0.29	0.85	2.51	137
Raceview	RR[2405]	0.41	0.68	1.09	7340
Radford	RR[2406]	0.21	0.65	1.99	41
Raglan (Qld)	RR[2407]	0.30	0.92	2.36	120
Railway Estate	RR[2408]	0.90	1.63	2.74	2388
Rainbow Beach	RR[2409]	0.31	0.90	2.20	1074
Ramsay (Qld)	RR[2410]	0.22	0.59	1.58	272
Rangemore (Burdekin - Qld)	RR[2411]	0.35	1.15	3.41	3
Rangemore (Toowoomba - Qld)	RR[2412]	0.25	0.82	2.36	3
Ranges Bridge	RR[2413]	0.28	0.80	2.03	106
Rangeville	RR[2414]	0.58	0.91	1.39	6717
Rangewood	RR[2415]	0.41	0.96	2.15	901
Ransome	RR[2416]	0.24	0.62	1.71	296
Rasmussen	RR[2417]	0.93	1.54	2.43	3398
Rathdowney	RR[2418]	0.51	1.33	3.48	252
Ravensbourne	RR[2419]	0.27	0.74	1.91	216
Ravenshoe	RR[2420]	**1.14**	**2.16**	**4.00**	1148
Ravenswood (Qld)	RR[2421]	0.44	1.17	2.92	220
Ravensworth (Qld)	RR[2422]	0.47	1.30	3.80	0
Rawbelle	RR[2423]	0.31	0.83	2.33	46
Red Hill (Brisbane - Qld)	RR[2424]	0.37	0.74	1.23	4734
Red Hill (Western Downs - Qld)	RR[2425]	0.28	0.84	2.49	46
Red River	RR[2426]	0.44	1.28	4.22	0
Redbank (Qld)	RR[2427]	0.43	0.97	2.01	1420
Redbank Creek	RR[2428]	0.28	0.79	2.27	118
Redbank Plains	RR[2429]	0.21	0.34	0.55	13640
Redcliffe (Qld)	RR[2430]	**1.29**	**1.77**	**2.33**	8979
Redford	RR[2431]	0.40	1.13	3.00	7
Redgate (Qld)	RR[2432]	0.29	0.80	2.37	52
Redhill Farms	RR[2433]	0.26	0.84	2.58	29
Redland Bay	RR[2434]	0.48	0.75	1.08	11706
Redlynch	RR[2435]	0.51	0.83	1.29	7176
Redridge	RR[2436]	0.49	1.13	2.61	540
Redwood	RR[2437]	0.26	0.75	2.07	149
Reedy Creek (Qld)	RR[2438]	0.19	0.39	0.73	4991
Reesville	RR[2439]	0.22	0.60	1.43	495
Regency Downs	RR[2440]	0.22	0.48	1.08	1754
Regents Park (Qld)	RR[2441]	0.43	0.69	1.05	8358
Reid River	RR[2442]	0.33	0.99	2.81	103
Reids Creek	RR[2443]	0.30	0.89	2.67	51
Retro	RR[2444]	0.25	0.83	2.58	21
Rewan	RR[2445]	0.36	1.05	3.10	22
Rhydding	RR[2446]	0.32	0.95	2.84	43
Richlands (Qld)	RR[2447]	0.13	0.32	0.69	2759
Richmond (Mackay - Qld)	RR[2448]	0.21	0.61	1.63	352
Richmond (Richmond - Qld)	RR[2449]	0.53	1.39	3.28	524

Richmond Hill (Qld)	RR[2450]	0.64	1.16	2.18	1939
Ridgelands	RR[2451]	0.31	0.91	2.56	137
Ridgewood (Qld)	RR[2452]	0.33	0.92	2.37	247
Rifle Range	RR[2453]	0.26	0.75	2.21	136
Ringtail Creek	RR[2454]	0.25	0.72	2.04	171
Ringwood (Qld)	RR[2455]	0.25	0.71	2.15	55
Riordanvale	RR[2456]	0.33	0.96	2.56	247
Ripley	RR[2457]	0.24	0.58	1.28	1130
Rita Island	RR[2458]	0.47	1.39	3.89	127
River Heads	RR[2459]	0.20	0.58	1.29	1312
River Ranch	RR[2460]	0.29	0.82	2.40	292
Riverhills	RR[2461]	0.11	0.28	0.63	3138
Riverleigh	RR[2462]	0.40	1.17	3.38	53
Riversleigh	RR[2463]	0.37	1.13	3.20	9
Riverton (Qld)	RR[2464]	0.26	0.92	3.17	10
Riverview (Qld)	RR[2465]	0.36	0.75	1.48	2389
Roadvale	RR[2466]	0.24	0.62	1.60	232
Robertson (Qld)	RR[2467]	0.27	0.54	0.98	4275
Robina	RR[2468]	0.57	0.80	1.06	19221
Roche Creek	RR[2469]	0.31	0.96	2.54	42
Rochedale	RR[2470]	0.28	0.63	1.23	2491
Rochedale South	RR[2471]	0.65	0.94	1.31	12072
Rockhampton City	RR[2472]	**7.07**	**9.60**	**12.55**	1717
Rockingham (Qld)	RR[2473]	0.35	1.04	2.94	88
Rocklea (Qld)	RR[2474]	**1.67**	**2.91**	**4.77**	1341
Rockmount	RR[2475]	0.23	0.65	1.95	76
Rocksberg	RR[2476]	0.29	0.83	2.18	240
Rockside	RR[2477]	0.24	0.67	1.98	35
Rockville	RR[2478]	0.38	0.72	1.42	2636
Rocky Creek (Qld)	RR[2479]	0.26	0.82	2.69	34
Rocky Point (Douglas - Qld)	RR[2480]	0.42	1.27	3.66	107
Rocky Point (Weipa - Qld)	RR[2481]	0.43	1.03	2.13	1384
Rockyview	RR[2482]	0.32	0.78	1.70	1242
Rodds Bay	RR[2483]	0.35	1.06	3.37	127
Rodgers Creek	RR[2484]	0.27	0.84	2.32	4
Rolleston	RR[2485]	0.52	1.37	3.54	237
Rollingstone	RR[2486]	0.47	1.34	3.63	103
Roma	RR[2487]	0.79	1.27	1.93	5319
Rookwood (Qld)	RR[2488]	0.42	1.26	3.51	0
Ropeley	RR[2489]	0.22	0.64	1.88	152
Rosalie Plains	RR[2490]	0.27	0.76	2.35	35
Rosedale (Qld)	RR[2491]	0.24	0.70	1.72	376
Rosehill (Qld)	RR[2492]	0.44	1.26	3.70	103
Rosella	RR[2493]	0.27	0.83	2.42	67
Rosemount	RR[2494]	0.28	0.67	1.53	1319
Roseneath	RR[2495]	0.31	0.90	2.35	139
Rosenthal Heights	RR[2496]	0.24	0.53	1.15	1735
Rosevale (Qld)	RR[2497]	0.20	0.62	1.69	170
Rosewood (Qld)	RR[2498]	0.67	1.30	2.18	2233
Ross Creek (Qld)	RR[2499]	0.27	0.79	2.27	45

Ross River	RR[2500]	0.36	0.99	2.97	0
Rosslea	RR[2501]	0.61	1.22	2.31	1473
Rosslyn	RR[2502]	0.33	0.93	2.33	476
Rossmoya	RR[2503]	0.44	1.24	3.65	55
Rossvale	RR[2504]	0.26	0.77	2.29	55
Rossville	RR[2505]	0.53	1.54	4.18	170
Rothwell	RR[2506]	0.55	0.92	1.45	5765
Round Hill (Qld)	RR[2507]	0.34	1.00	2.85	115
Roundstone	RR[2508]	0.37	0.96	2.64	24
Rowes Bay	RR[2509]	0.61	1.56	3.39	510
Royston	RR[2510]	0.32	0.87	2.38	276
Rubyanna	RR[2511]	0.32	0.89	2.32	192
Rules Beach	RR[2512]	0.28	0.82	2.41	65
Runaway Bay	RR[2513]	**1.49**	**2.01**	**2.64**	7955
Runcorn	RR[2514]	0.24	0.42	0.67	11863
Rungoo	RR[2515]	0.43	1.41	4.21	12
Running Creek (Qld)	RR[2516]	0.27	0.72	2.33	115
Runnymede (Qld)	RR[2517]	0.34	1.01	2.74	141
Rural View	RR[2518]	0.19	0.41	0.81	3535
Rush Creek	RR[2519]	0.29	0.78	2.21	131
Russell Island	RR[2520]	0.85	1.64	2.80	2480
Ryan (Qld)	RR[2521]	0.48	1.42	3.76	123
Ryeford	RR[2522]	0.29	0.80	2.24	43
Rywung	RR[2523]	0.26	0.89	2.58	24
Sabine	RR[2524]	0.27	0.77	2.48	6
Sadliers Crossing	RR[2525]	0.42	1.05	2.10	1089
Saibai Island	RR[2526]	0.50	1.84	5.25	268
Salisbury (Qld)	RR[2527]	0.67	1.12	1.81	5098
Saltern Creek	RR[2528]	0.40	1.26	4.50	9
Samford Valley	RR[2529]	0.27	0.58	1.11	2425
Samford Village	RR[2530]	0.29	0.84	2.31	619
Samsonvale	RR[2531]	0.22	0.58	1.49	429
Sandgate (Qld)	RR[2532]	**2.24**	**3.11**	**4.20**	4086
Sandiford	RR[2533]	0.34	1.00	2.77	146
Sandringham (Qld)	RR[2534]	0.30	0.96	3.06	45
Sandstone Point	RR[2535]	0.55	1.03	1.73	3470
Sandy Camp	RR[2536]	0.28	0.80	2.18	75
Sandy Creek (Qld)	RR[2537]	0.24	0.64	1.57	469
Sandy Pocket	RR[2538]	0.41	1.23	3.85	33
Sandy Ridges	RR[2539]	0.30	0.84	2.41	70
Sarabah	RR[2540]	0.34	0.95	2.73	55
Sardine	RR[2541]	0.43	1.38	3.73	5
Sarina	RR[2542]	**1.18**	**1.83**	**2.67**	4387
Sarina Beach	RR[2543]	0.47	1.26	2.90	489
Sarina Range	RR[2544]	0.24	0.76	2.08	199
Saunders Beach	RR[2545]	0.40	1.20	3.16	356
Savannah	RR[2546]	0.52	1.47	4.57	0
Saxby	RR[2547]	0.45	1.41	4.38	12
Scarborough (Qld)	RR[2548]	**1.56**	**2.10**	**2.83**	7394
Scarness	RR[2549]	0.85	1.45	2.41	3049

Scotchy Pocket	RR[2550]	0.29	0.88	2.47	61
Scottville	RR[2551]	0.29	0.97	3.07	205
Scrub Creek	RR[2552]	0.29	0.84	2.52	23
Scrubby Creek (Blackall-Tambo - Ql	RR[2553]	0.38	1.21	3.71	5
Scrubby Creek (Gympie - Qld)	RR[2554]	0.23	0.74	2.17	51
Scrubby Mountain	RR[2555]	0.25	0.74	2.20	124
Seaforth (Qld)	RR[2556]	0.62	1.59	3.39	670
Sedgeford	RR[2557]	0.37	1.08	3.02	0
Seisia	RR[2558]	0.19	0.75	2.61	178
Selene	RR[2559]	0.27	0.80	2.45	19
Selwyn (Qld)	RR[2560]	0.49	1.42	4.06	50
Septimus	RR[2561]	0.27	0.81	2.16	101
Seven Hills (Qld)	RR[2562]	0.59	1.16	2.19	1676
Seventeen Mile	RR[2563]	0.27	0.76	2.22	13
Seventeen Mile Rocks	RR[2564]	0.23	0.55	1.10	2021
Seventeen Seventy	RR[2565]	0.19	0.85	3.29	66
Seventy Mile	RR[2566]	0.36	0.93	2.61	183
Severnlea	RR[2567]	0.27	0.78	2.14	277
Sexton	RR[2568]	0.28	0.81	2.26	123
Shailer Park	RR[2569]	0.28	0.49	0.77	9517
Shannonvale	RR[2570]	0.70	1.81	4.36	156
Sharon	RR[2571]	0.30	0.73	1.64	1078
Shaw	RR[2572]	0.24	0.64	1.79	429
Sheep Station Creek	RR[2573]	0.29	0.78	2.27	85
Shelburne	RR[2574]	0.33	1.13	3.20	23
Sheldon	RR[2575]	0.36	0.74	1.53	1413
Shell Pocket	RR[2576]	0.38	1.13	3.29	65
Shelly Beach (Sunshine Coast - Qld)	RR[2577]	0.24	0.76	1.94	726
Shelly Beach (Townsville - Qld)	RR[2578]	0.36	1.28	4.62	0
Sherwood (Qld)	RR[2579]	0.57	0.96	1.56	4294
Shirbourne	RR[2580]	0.38	1.11	3.07	57
Shoal Point	RR[2581]	0.20	0.61	1.54	773
Shoalwater (Qld)	RR[2582]	0.33	0.97	2.85	0
Shorncliffe	RR[2583]	0.66	1.35	2.55	1512
Shute Harbour	RR[2584]	0.15	0.71	2.86	108
Silkstone	RR[2585]	0.58	1.08	1.87	2799
Silkwood	RR[2586]	0.47	1.14	2.96	309
Silky Oak	RR[2587]	0.39	1.06	2.96	112
Silver Creek	RR[2588]	0.29	0.98	3.39	5
Silver Ridge	RR[2589]	0.23	0.68	1.83	141
Silver Spur	RR[2590]	0.31	0.94	2.96	58
Silver Valley	RR[2591]	0.50	1.30	3.74	130
Silverdale (Qld)	RR[2592]	0.24	0.67	1.88	95
Silverleaf	RR[2593]	0.28	0.80	2.31	38
Silverleigh	RR[2594]	0.29	0.75	2.19	60
Silverwood	RR[2595]	0.27	0.78	2.36	34
Simmie	RR[2596]	0.33	1.08	3.23	5
Sinnamon Park	RR[2597]	0.22	0.44	0.78	5152
Sippy Downs	RR[2598]	0.24	0.41	0.68	8192
Skyring Reserve	RR[2599]	0.28	0.84	2.52	32

Slacks Creek	RR[2600]	0.70	1.03	1.50	8060
Slade Point	RR[2601]	0.65	1.18	2.02	2670
Sladevale	RR[2602]	0.24	0.68	1.72	282
Smithfield (Qld)	RR[2603]	0.67	1.13	1.80	4250
Smithlea	RR[2604]	0.28	1.02	3.01	43
Smoky Creek	RR[2605]	0.49	1.45	3.84	128
Soldiers Hill (Qld)	RR[2606]	0.79	1.56	2.83	1456
Somerset (Qld)	RR[2607]	0.24	0.88	2.81	0
Somerset Dam	RR[2608]	0.29	0.82	2.50	65
Sommariva	RR[2609]	0.38	1.13	3.48	10
Somme	RR[2610]	0.29	0.91	2.66	43
South Bingera	RR[2611]	0.34	0.89	2.11	532
South Brisbane	RR[2612]	0.72	1.14	1.68	6576
South East Nanango	RR[2613]	0.37	0.96	2.53	240
South Gladstone	RR[2614]	0.43	0.83	1.57	2672
South Innisfail	RR[2615]	0.51	1.32	3.07	420
South Isis	RR[2616]	0.32	0.90	2.35	281
South Johnstone	RR[2617]	0.72	1.80	4.21	347
South Kolan	RR[2618]	0.26	0.63	1.48	841
South Mackay	RR[2619]	0.75	1.21	1.79	5565
South Maclean	RR[2620]	0.41	0.93	2.07	1092
South Mission Beach	RR[2621]	0.34	0.89	2.15	757
South Nanango	RR[2622]	0.28	0.76	1.76	739
South Ripley	RR[2623]	0.28	0.69	1.68	517
South Stradbroke	RR[2624]	0.19	0.69	2.95	38
South Talwood	RR[2625]	0.28	0.87	2.76	64
South Toowoomba	RR[2626]	0.94	1.49	2.21	4326
South Townsville	RR[2627]	**1.21**	**2.14**	**3.42**	2072
South Trees	RR[2628]	0.24	0.74	2.33	0
South Wellesley Islands	RR[2629]	0.00	0.00	1.65	0
South Yaamba	RR[2630]	0.31	0.92	2.75	80
Southbrook	RR[2631]	0.27	0.72	1.78	489
Southedge	RR[2632]	0.43	1.19	3.36	21
Southern Cross (Qld)	RR[2633]	0.41	1.12	2.51	417
Southern Lamington	RR[2634]	0.24	0.78	2.53	0
Southern Moreton Bay Islands	RR[2635]	0.22	0.74	2.18	0
Southport (Qld)	RR[2636]	**1.02**	**1.26**	**1.52**	27923
Southside	RR[2637]	0.45	0.80	1.32	4598
Southwood	RR[2638]	0.32	0.87	2.38	64
Speedwell	RR[2639]	0.35	0.92	3.08	21
Speewah	RR[2640]	0.33	0.90	2.01	722
Splinter Creek	RR[2641]	0.29	0.85	2.54	23
Split Yard Creek	RR[2642]	0.26	0.81	2.32	31
Spreadborough	RR[2643]	0.48	1.58	4.64	20
Spring Bluff	RR[2644]	0.23	0.68	2.01	6
Spring Creek (Banana - Qld)	RR[2645]	0.33	1.00	3.06	12
Spring Creek (Lockyer Valley - Qld)	RR[2646]	0.23	0.62	1.45	561
Spring Creek (Toowoomba - Qld)	RR[2647]	0.26	0.75	2.25	157
Spring Hill (Qld)	RR[2648]	0.94	1.43	2.05	5601
Spring Mountain (Qld)	RR[2649]	0.22	0.62	1.73	0

Springbrook	RR[2650]	0.19	0.55	1.41	551
Springdale (Qld)	RR[2651]	0.30	0.93	2.62	18
Springfield (Ipswich - Qld)	RR[2652]	0.17	0.36	0.69	4948
Springfield (Mareeba - Qld)	RR[2653]	0.44	1.27	3.84	0
Springfield Central	RR[2654]	0.18	0.53	1.56	114
Springfield Lakes	RR[2655]	0.17	0.31	0.53	10973
Springlands	RR[2656]	0.37	1.05	3.39	39
Springside (Qld)	RR[2657]	0.24	0.75	2.19	91
Springsure	RR[2658]	0.42	1.12	2.45	865
Springvale (Qld)	RR[2659]	0.26	0.76	2.15	60
Springwood (Qld)	RR[2660]	0.31	0.54	0.92	7425
Spurgeon	RR[2661]	0.44	1.29	4.14	0
St Agnes (Qld)	RR[2662]	0.29	0.88	2.50	19
St Aubyn	RR[2663]	0.27	0.85	2.38	8
St George (Qld)	RR[2664]	0.67	1.25	2.16	2358
St Helens (Fraser Coast - Qld)	RR[2665]	0.29	0.82	2.40	97
St Helens (Toowoomba - Qld)	RR[2666]	0.26	0.76	2.23	18
St Helens Beach	RR[2667]	0.30	0.90	2.51	160
St Kilda (Qld)	RR[2668]	0.28	0.81	2.34	74
St Lawrence	RR[2669]	**1.05**	**2.53**	**6.41**	210
St Lucia	RR[2670]	0.22	0.38	0.61	11498
St Mary	RR[2671]	0.31	0.89	2.41	63
St Ruth	RR[2672]	0.25	0.74	2.04	108
Staaten	RR[2673]	0.44	1.38	4.10	0
Stafford	RR[2674]	**1.07**	**1.60**	**2.34**	5483
Stafford Heights	RR[2675]	**1.01**	**1.50**	**2.11**	5593
Stalworth	RR[2676]	0.32	0.91	2.64	30
Stamford	RR[2677]	0.50	1.33	3.62	38
Stanage	RR[2678]	0.28	0.92	3.05	80
Stanmore (Qld)	RR[2679]	0.32	0.84	2.05	348
Stanthorpe	RR[2680]	0.73	1.20	1.85	4489
Stanwell	RR[2681]	0.55	1.42	3.21	259
Stapylton	RR[2682]	0.26	0.72	1.88	367
Starcke	RR[2683]	0.41	1.33	4.36	3
Steiglitz (Qld)	RR[2684]	0.34	0.89	2.16	588
Stewart Creek Valley	RR[2685]	0.40	1.30	3.65	18
Stewarton (Qld)	RR[2686]	0.30	0.93	2.73	31
Stockhaven	RR[2687]	0.31	0.92	2.64	5
Stockleigh	RR[2688]	0.34	0.82	1.89	606
Stockton (Qld)	RR[2689]	0.47	1.42	4.09	17
Stockyard (Livingstone - Qld)	RR[2690]	0.29	0.94	2.83	16
Stockyard (Lockyer Valley - Qld)	RR[2691]	0.25	0.68	2.00	44
Stokes	RR[2692]	0.53	1.48	3.89	69
Stonehenge (Barcoo - Qld)	RR[2693]	0.49	1.33	3.58	41
Stonehenge (Toowoomba - Qld)	RR[2694]	0.31	0.85	2.35	27
Stonelands	RR[2695]	0.32	0.86	2.38	34
Stoneleigh (Qld)	RR[2696]	0.25	0.71	1.93	92
Stony Creek (Qld)	RR[2697]	0.24	0.68	1.87	214
Storm King	RR[2698]	0.30	0.89	2.87	81
Stoters Hill	RR[2699]	0.43	1.18	3.54	90

Stratford (Qld)	RR[2700]	0.65	1.39	2.77	907
Strathdickie	RR[2701]	0.30	0.80	1.95	678
Strathfield (Qld)	RR[2702]	0.30	0.88	2.62	8
Strathmore (Qld)	RR[2703]	0.46	1.38	3.51	8
Strathpine	RR[2704]	0.78	1.15	1.64	7696
Stretton	RR[2705]	0.12	0.27	0.57	3524
Struck Oil	RR[2706]	0.44	1.35	3.73	158
Stuart (Qld)	RR[2707]	0.53	1.13	2.24	1305
Sturt (Qld)	RR[2708]	0.42	1.48	5.05	0
Sugarloaf (Southern Downs - Qld)	RR[2709]	0.30	0.91	2.76	98
Sugarloaf (Whitsunday - Qld)	RR[2710]	0.23	0.77	2.15	221
Sujeewong	RR[2711]	0.32	0.94	2.63	11
Summerholm	RR[2712]	0.20	0.57	1.35	541
Sumner	RR[2713]	0.20	0.60	1.56	478
Sun Valley (Qld)	RR[2714]	0.44	1.04	2.08	986
Sundown (Cassowary Coast - Qld)	RR[2715]	0.37	1.09	2.88	157
Sundown (Southern Downs - Qld)	RR[2716]	0.31	0.99	3.29	0
Sunny Nook	RR[2717]	0.27	0.83	2.37	20
Sunnybank	RR[2718]	0.47	0.77	1.18	7589
Sunnybank Hills	RR[2719]	0.34	0.52	0.77	15035
Sunnyside (Qld)	RR[2720]	0.26	0.81	2.02	115
Sunrise Beach	RR[2721]	0.41	0.82	1.59	2755
Sunset	RR[2722]	0.75	1.42	2.64	1621
Sunshine Acres	RR[2723]	0.22	0.59	1.38	757
Sunshine Beach	RR[2724]	0.48	0.98	1.95	2073
Surat	RR[2725]	0.32	0.98	2.44	332
Surbiton	RR[2726]	0.36	1.01	2.77	66
Surfers Paradise	RR[2727]	0.70	0.91	1.16	21739
Susan River	RR[2728]	0.31	0.82	2.49	101
Suttor	RR[2729]	0.32	0.94	2.87	0
Svensson Heights	RR[2730]	**1.14**	**1.92**	**3.03**	2661
Swan Creek (Qld)	RR[2731]	0.24	0.72	1.97	112
Swanbank	RR[2732]	0.26	0.77	2.05	0
Swanfels	RR[2733]	0.23	0.67	1.86	107
Swans Lagoon	RR[2734]	0.34	1.08	3.59	0
Sweers Island	RR[2735]	0.00	0.00	1.04	5
Syndicate	RR[2736]	0.44	1.40	3.93	0
Taabinga	RR[2737]	0.24	0.67	1.78	443
Tablederry	RR[2738]	0.47	1.38	3.91	26
Tablelands (Gladstone - Qld)	RR[2739]	0.30	0.88	2.33	0
Tablelands (South Burnett - Qld)	RR[2740]	0.28	0.81	2.30	88
Tabooba	RR[2741]	0.26	0.74	2.31	54
Tabragalba	RR[2742]	0.26	0.75	2.15	39
Taigum	RR[2743]	0.33	0.61	1.05	5391
Takilberan	RR[2744]	0.28	0.83	2.43	17
Takura	RR[2745]	0.34	0.90	2.27	403
Talaroo	RR[2746]	0.41	1.37	4.33	0
Taldora	RR[2747]	0.54	1.48	4.27	34
Talegalla Weir	RR[2748]	0.31	0.86	2.32	114
Talgai	RR[2749]	0.30	0.78	2.15	87

Tallai	RR[2750]	0.23	0.49	0.92	3397
Tallebudgera	RR[2751]	0.78	1.39	2.26	2934
Tallebudgera Valley	RR[2752]	0.29	0.68	1.46	1308
Tallegalla	RR[2753]	0.35	1.03	2.62	274
Tam O'Shanter	RR[2754]	0.39	1.13	3.28	0
Tamaree	RR[2755]	0.20	0.56	1.36	647
Tambo	RR[2756]	0.42	1.19	3.08	304
Tamborine	RR[2757]	0.50	0.92	1.63	3172
Tamborine Mountain	RR[2758]	0.61	0.98	1.48	6209
Tamrookum	RR[2759]	0.25	0.72	2.16	72
Tamrookum Creek	RR[2760]	0.24	0.77	2.55	29
Tanah Merah	RR[2761]	0.32	0.62	1.16	3758
Tanawha	RR[2762]	0.26	0.57	1.36	1051
Tanbar	RR[2763]	0.38	1.28	3.70	3
Tanby	RR[2764]	0.41	1.06	2.57	404
Tandora	RR[2765]	0.28	0.88	2.57	3
Tandur	RR[2766]	0.23	0.65	1.85	157
Tangorin	RR[2767]	0.43	1.32	3.69	38
Tannum Sands	RR[2768]	0.18	0.39	0.80	3904
Tannymorel	RR[2769]	0.23	0.70	1.97	132
Tansey	RR[2770]	0.29	0.82	2.19	111
Tara (Qld)	RR[2771]	0.91	1.66	2.80	1912
Tara Station	RR[2772]	0.43	1.24	3.91	10
Taragoola	RR[2773]	0.27	0.76	2.54	0
Tarampa	RR[2774]	0.32	0.85	2.52	263
Taranganba	RR[2775]	0.23	0.57	1.21	1815
Tarawera	RR[2776]	0.34	0.89	2.89	28
Targinnie	RR[2777]	0.30	0.94	2.73	55
Taringa	RR[2778]	0.20	0.38	0.65	7435
Tarome	RR[2779]	0.22	0.68	1.82	92
Taromeo	RR[2780]	0.27	0.75	1.90	285
Tarong	RR[2781]	0.27	0.84	2.24	141
Taroom	RR[2782]	0.75	1.61	3.27	695
Taroomball	RR[2783]	0.27	0.72	1.72	652
Tarragindi	RR[2784]	0.80	1.18	1.68	8351
Tarramba	RR[2785]	0.27	0.87	2.72	43
Tarzali	RR[2786]	0.33	0.90	2.21	331
Taunton (Qld)	RR[2787]	0.29	0.82	2.28	58
Taylors Beach (Qld)	RR[2788]	0.49	1.43	3.61	296
Te Kowai	RR[2789]	0.29	0.76	2.14	182
Teddington	RR[2790]	0.29	0.79	2.10	206
Teebar	RR[2791]	0.29	0.86	2.68	37
Teelah	RR[2792]	0.29	0.85	2.50	52
Teelba	RR[2793]	0.29	0.90	2.63	28
Telina	RR[2794]	0.21	0.48	1.05	1654
Tellebang	RR[2795]	0.28	0.80	2.36	52
Templin	RR[2796]	0.23	0.73	2.07	80
Teneriffe	RR[2797]	0.14	0.31	0.62	4962
Tennyson (Qld)	RR[2798]	0.32	0.78	1.75	853
Terrica	RR[2799]	0.30	0.91	2.75	14

Teviotville	RR[2800]	0.23	0.72	1.94	99
Tewantin	RR[2801]	1.00	1.40	1.90	9238
Texas (Qld)	RR[2802]	0.56	1.42	3.10	710
Thabeban	RR[2803]	0.47	0.94	1.80	2222
Thagoona	RR[2804]	0.20	0.51	1.25	813
Thallon	RR[2805]	0.25	0.81	2.27	200
Thane	RR[2806]	0.28	0.86	2.73	27
Thanes Creek	RR[2807]	0.27	0.85	2.71	30
Thangool	RR[2808]	0.30	0.79	1.82	588
Thargomindah	RR[2809]	0.30	0.97	2.94	225
The Bluff (Ipswich - Qld)	RR[2810]	0.23	0.71	1.97	35
The Bluff (Toowoomba - Qld)	RR[2811]	0.31	0.86	2.53	0
The Caves	RR[2812]	0.42	1.02	2.43	573
The Common	RR[2813]	0.42	1.27	4.03	0
The Dawn	RR[2814]	0.20	0.59	1.50	436
The Dimonds	RR[2815]	0.30	0.93	2.99	0
The Falls	RR[2816]	0.23	0.71	2.18	53
The Gap (Brisbane - Qld)	RR[2817]	0.83	1.15	1.51	13134
The Gap (Mount Isa - Qld)	RR[2818]	0.37	0.91	2.29	672
The Gemfields	RR[2819]	0.46	1.04	2.10	1288
The Glen (Qld)	RR[2820]	0.28	0.82	2.46	27
The Gums	RR[2821]	0.35	1.09	2.99	126
The Head	RR[2822]	0.20	0.70	2.32	7
The Hermitage	RR[2823]	0.26	0.75	2.19	46
The Keppels	RR[2824]	0.21	0.90	3.61	47
The Leap	RR[2825]	0.28	0.76	1.78	532
The Limits	RR[2826]	0.28	0.91	2.64	4
The Mine	RR[2827]	0.38	1.13	3.36	41
The Narrows (Qld)	RR[2828]	0.37	0.97	2.70	3
The Palms	RR[2829]	0.20	0.51	1.23	845
The Percy Group	RR[2830]	0.19	0.94	4.12	0
The Pines (Qld)	RR[2831]	0.27	0.81	2.55	45
The Range (Qld)	RR[2832]	0.69	1.16	1.84	4275
The Summit	RR[2833]	0.31	0.94	2.41	338
Theebine	RR[2834]	0.40	1.18	3.23	87
Theodore (Qld)	RR[2835]	0.56	1.55	3.73	372
Theresa Creek (Qld)	RR[2836]	0.32	0.91	2.76	33
Thinoomba	RR[2837]	0.31	0.91	2.47	31
Thompson Point	RR[2838]	0.34	1.03	3.28	10
Thoopara	RR[2839]	0.35	0.99	2.88	31
Thornborough	RR[2840]	0.42	1.16	3.69	6
Thorndale	RR[2841]	0.36	1.11	3.12	120
Thorneside	RR[2842]	0.38	0.73	1.31	3079
Thornlands	RR[2843]	0.48	0.73	1.04	11620
Thornton (Qld)	RR[2844]	0.29	0.82	2.15	165
Thornton Beach	RR[2845]	0.41	1.42	4.88	5
Thornville	RR[2846]	0.38	1.08	3.34	23
Three Moon	RR[2847]	0.29	0.82	2.28	119
Three Rivers	RR[2848]	0.65	2.10	5.94	27
Thulimbah	RR[2849]	0.42	1.23	3.34	272

Thuringowa Central	RR[2850]	0.21	0.52	1.19	1637
Thursday Island	RR[2851]	0.74	1.50	2.73	2117
Tiaro	RR[2852]	0.57	1.41	3.00	646
Tieri	RR[2853]	0.15	0.47	1.46	766
Tin Can Bay	RR[2854]	**1.45**	**2.52**	**4.01**	2020
Tinana	RR[2855]	0.29	0.57	0.96	4449
Tinana South	RR[2856]	0.25	0.69	1.73	409
Tinaroo	RR[2857]	0.42	1.15	2.82	268
Tinbeerwah	RR[2858]	0.73	1.57	3.12	835
Tingalpa	RR[2859]	0.53	0.83	1.33	6738
Tingoora	RR[2860]	0.25	0.75	1.96	227
Tingun	RR[2861]	0.34	0.90	2.36	116
Tinnanbar	RR[2862]	0.22	0.84	2.67	115
Tipton	RR[2863]	0.26	0.78	2.25	26
Tirroan	RR[2864]	0.24	0.76	2.22	152
Tivoli	RR[2865]	0.72	1.45	2.78	1182
Tocal (Qld)	RR[2866]	0.45	1.38	4.16	14
Togara	RR[2867]	0.30	0.91	2.70	7
Toko	RR[2868]	0.47	1.55	4.75	4
Tolga	RR[2869]	0.54	1.06	1.92	2181
Toll	RR[2870]	0.49	1.27	2.82	530
Toobanna	RR[2871]	0.40	1.06	2.68	227
Toobeah	RR[2872]	0.36	1.07	2.92	144
Toogoolawah	RR[2873]	0.33	0.80	1.80	1077
Toogoom	RR[2874]	0.58	1.15	2.19	1743
Toolakea	RR[2875]	0.19	0.76	2.86	166
Toolara Forest	RR[2876]	0.31	0.88	2.49	0
Toolburra	RR[2877]	0.26	0.80	2.34	34
Toolooa	RR[2878]	0.24	0.67	1.64	701
Toomulla	RR[2879]	0.34	1.27	3.92	164
Toondahra	RR[2880]	0.30	0.90	2.75	8
Toonpan	RR[2881]	0.33	1.00	3.01	53
Toorbul	RR[2882]	0.35	0.88	2.15	829
Toowong	RR[2883]	0.47	0.73	1.07	9669
Toowoomba City	RR[2884]	**3.19**	**4.91**	**6.98**	1839
Top Camp	RR[2885]	0.20	0.54	1.33	665
Topaz	RR[2886]	0.45	1.38	3.78	126
Torbanlea	RR[2887]	0.44	1.05	2.32	623
Torquay (Qld)	RR[2888]	**1.18**	**1.75**	**2.46**	5434
Torrens Creek	RR[2889]	0.41	1.14	3.28	67
Torres Strait	RR[2890]	0.35	1.34	4.37	0
Torrington (Qld)	RR[2891]	0.32	0.81	2.01	726
Towen Mountain	RR[2892]	0.24	0.69	1.87	239
Towers Hill	RR[2893]	0.39	1.11	2.71	190
Town Common	RR[2894]	0.36	1.00	2.86	71
Townson	RR[2895]	0.24	0.68	1.98	38
Townsville City	RR[2896]	**1.62**	**2.51**	**3.76**	2682
Townview	RR[2897]	0.77	1.53	2.79	1565
Traveston	RR[2898]	0.20	0.59	1.53	395
Trebonne	RR[2899]	0.46	1.22	3.10	350

Tregony	RR[2900]	0.32	0.90	2.69	28
Trinity Beach	RR[2901]	0.50	0.88	1.46	4424
Trinity Park	RR[2902]	0.38	0.79	1.45	2403
Trotter Creek	RR[2903]	0.37	1.13	3.57	10
Trunding	RR[2904]	0.24	0.64	1.58	752
Tuan	RR[2905]	0.36	1.10	3.26	136
Tuan Forest	RR[2906]	0.30	0.91	2.54	0
Tuchekoi	RR[2907]	0.26	0.73	2.00	155
Tuckerang	RR[2908]	0.29	0.82	2.34	52
Tuen	RR[2909]	0.35	1.13	3.59	12
Tugun	RR[2910]	0.99	1.52	2.21	5515
Tully	RR[2911]	**2.08**	**3.30**	**5.01**	1960
Tully Heads	RR[2912]	0.32	0.91	2.46	305
Tummaville	RR[2913]	0.29	0.80	2.35	54
Tumoulin	RR[2914]	0.35	1.05	3.08	93
Tungamull	RR[2915]	0.28	0.83	2.24	355
Turallin	RR[2916]	0.29	0.82	2.32	54
Turkey Beach	RR[2917]	0.30	1.08	3.99	165
Turrawulla	RR[2918]	0.32	0.94	2.75	7
Twin Waters	RR[2919]	0.14	0.36	0.86	2357
Two Mile	RR[2920]	0.26	0.74	2.13	78
Tyrconnel	RR[2921]	0.37	1.11	3.25	26
Ugar Island	RR[2922]	0.21	1.03	4.91	50
Ulogie	RR[2923]	0.33	0.99	2.72	21
Umagico	RR[2924]	0.23	0.76	2.10	253
Umbiram	RR[2925]	0.24	0.67	1.97	108
Underwood (Qld)	RR[2926]	0.23	0.47	0.85	4865
Undullah	RR[2927]	0.23	0.64	2.03	35
Upland	RR[2928]	0.44	1.14	3.98	12
Upper Barron	RR[2929]	0.33	0.89	2.21	357
Upper Brookfield	RR[2930]	0.27	0.71	1.75	616
Upper Caboolture	RR[2931]	0.18	0.42	0.85	3190
Upper Coomera	RR[2932]	0.16	0.27	0.41	18254
Upper Cooyar Creek	RR[2933]	0.27	0.85	2.24	8
Upper Cornish Creek	RR[2934]	0.41	1.23	3.52	36
Upper Daintree	RR[2935]	0.43	1.40	4.29	6
Upper Daradgee	RR[2936]	0.51	1.53	4.17	85
Upper Dawson	RR[2937]	0.36	1.06	3.27	17
Upper Flagstone	RR[2938]	0.24	0.63	1.73	146
Upper Freestone	RR[2939]	0.23	0.68	1.94	76
Upper Glastonbury	RR[2940]	0.26	0.78	2.14	36
Upper Haughton	RR[2941]	0.37	1.05	2.86	62
Upper Kandanga	RR[2942]	0.28	0.81	2.18	54
Upper Kedron	RR[2943]	0.25	0.52	0.95	2972
Upper Lockyer	RR[2944]	0.21	0.56	1.43	431
Upper Mount Gravatt	RR[2945]	0.76	1.16	1.63	7912
Upper Pilton	RR[2946]	0.25	0.72	2.19	58
Upper Pinelands	RR[2947]	0.28	0.84	2.62	23
Upper Stone	RR[2948]	0.43	1.12	3.01	77
Upper Tenthill	RR[2949]	0.20	0.62	1.67	183

Upper Warrego	RR[2950]	0.34	1.09	3.25	0
Upper Wheatvale	RR[2951]	0.24	0.83	2.52	45
Upper Yarraman	RR[2952]	0.29	0.82	2.21	83
Urangan	RR[2953]	0.83	1.21	1.71	8228
Urraween	RR[2954]	0.27	0.50	0.88	5850
Utchee Creek	RR[2955]	0.34	0.98	2.72	203
V Gate	RR[2956]	0.38	1.02	3.05	20
Valdora	RR[2957]	0.20	0.60	1.56	426
Vale View	RR[2958]	0.19	0.58	1.49	348
Valentine Plains	RR[2959]	0.28	0.74	1.82	313
Valkyrie	RR[2960]	0.26	0.79	2.17	96
Valley Of Lagoons	RR[2961]	0.42	1.08	3.21	45
Varsity Lakes	RR[2962]	0.57	0.81	1.14	12134
Vasa Views	RR[2963]	0.42	1.11	3.03	109
Ventnor (Qld)	RR[2964]	0.26	0.82	2.37	14
Veradilla	RR[2965]	0.22	0.65	1.80	85
Veresdale	RR[2966]	0.23	0.62	1.60	322
Veresdale Scrub	RR[2967]	0.27	0.72	1.85	371
Vergemont	RR[2968]	0.51	1.45	4.15	26
Vernor	RR[2969]	0.25	0.72	1.92	192
Verrierdale	RR[2970]	0.34	0.89	1.95	615
Veteran	RR[2971]	0.27	0.68	1.66	708
Victoria Hill	RR[2972]	0.27	0.81	2.46	24
Victoria Plains	RR[2973]	0.24	0.65	1.76	301
Victoria Plantation	RR[2974]	0.58	1.60	4.08	137
Victoria Point (Qld)	RR[2975]	0.64	0.90	1.25	12419
Victoria Vale	RR[2976]	0.51	1.45	4.44	0
Victory Heights (Qld)	RR[2977]	0.29	0.75	1.84	453
Villeneuve	RR[2978]	0.46	1.19	3.47	142
Vincent	RR[2979]	0.60	1.16	2.17	1752
Vinegar Hill	RR[2980]	0.28	0.76	2.04	47
Virginia (Qld)	RR[2981]	0.77	1.48	2.63	1762
Wacol	RR[2982]	0.50	0.94	1.60	3651
Wahoon	RR[2983]	0.29	0.92	2.72	0
Waikola	RR[2984]	0.32	0.96	2.89	22
Wainui	RR[2985]	0.24	0.78	2.48	0
Wairuna	RR[2986]	0.40	1.23	3.55	0
Wakerley	RR[2987]	0.23	0.44	0.78	5980
Walhallow	RR[2988]	0.35	1.09	3.01	10
Walkamin	RR[2989]	0.55	1.31	3.27	393
Walkers Point	RR[2990]	0.31	0.91	2.48	91
Walkerston	RR[2991]	0.40	0.82	1.53	2661
Walkervale	RR[2992]	0.77	1.37	2.36	2368
Wallaces Creek	RR[2993]	0.23	0.68	1.95	58
Wallaman	RR[2994]	0.39	1.22	3.85	0
Wallangarra	RR[2995]	0.52	1.57	3.72	384
Wallaroo (Qld)	RR[2996]	0.31	0.98	2.97	6
Wallaville	RR[2997]	0.43	1.15	2.84	316
Walligan	RR[2998]	0.47	1.15	2.84	305
Walloon	RR[2999]	0.20	0.47	1.06	1316

Wallu	RR[3000]	0.29	0.93	2.95	66
Wallumbilla	RR[3001]	0.24	0.74	2.11	300
Wallumbilla North	RR[3002]	0.32	0.87	2.29	114
Wallumbilla South	RR[3003]	0.32	0.88	2.49	50
Walmul	RR[3004]	0.36	1.09	3.31	25
Walter Hill	RR[3005]	0.44	1.20	3.12	0
Walter Lever Estate	RR[3006]	0.40	1.23	3.59	45
Walterhall	RR[3007]	0.36	1.10	3.20	117
Wamuran	RR[3008]	0.55	0.99	1.77	2576
Wamuran Basin	RR[3009]	0.31	0.90	2.60	112
Wandal	RR[3010]	0.62	1.16	1.87	3331
Wandoan	RR[3011]	0.65	1.62	3.40	483
Wangan	RR[3012]	**1.06**	**2.32**	**4.67**	508
Wangaratta (Qld)	RR[3013]	0.37	1.17	3.52	17
Wangetti	RR[3014]	0.34	1.07	3.29	47
Wanjuru	RR[3015]	0.43	1.26	4.56	0
Wanora	RR[3016]	0.26	0.70	1.80	234
Warana	RR[3017]	0.87	1.46	2.38	2981
Warburton (Qld)	RR[3018]	0.49	1.56	4.62	0
Ward	RR[3019]	0.42	1.15	3.33	55
Warenda	RR[3020]	0.50	1.52	4.33	0
Warkon	RR[3021]	0.32	0.93	2.71	33
Warner	RR[3022]	0.33	0.56	0.89	8530
Warnoah	RR[3023]	0.32	0.93	2.70	39
Warnung	RR[3024]	0.25	0.85	2.54	11
Warra	RR[3025]	0.47	1.28	3.81	153
Warraber Islet	RR[3026]	0.63	2.12	6.57	163
Warrami	RR[3027]	0.33	1.11	3.87	48
Warrill View	RR[3028]	0.22	0.60	1.75	198
Warroo (Qld)	RR[3029]	0.29	0.92	2.66	25
Warrubullen	RR[3030]	0.40	1.23	3.53	51
Warwick (Qld)	RR[3031]	0.79	1.14	1.60	9741
Washpool (Qld)	RR[3032]	0.22	0.64	1.80	75
Watalgan	RR[3033]	0.26	0.85	2.68	38
Wateranga	RR[3034]	0.32	0.93	2.96	3
Waterford (Qld)	RR[3035]	0.30	0.57	1.05	4105
Waterford West	RR[3036]	0.39	0.70	1.20	5142
Waterloo (Qld)	RR[3037]	0.27	0.76	2.23	131
Watsons Crossing	RR[3038]	0.25	0.91	3.40	12
Watsonville	RR[3039]	0.39	1.02	2.83	158
Wattle Camp	RR[3040]	0.33	0.86	2.12	422
Wattle Grove (Qld)	RR[3041]	0.29	0.85	2.43	68
Wattle Ridge (Qld)	RR[3042]	0.27	0.87	2.51	33
Wattlebank	RR[3043]	0.33	0.96	3.05	64
Waugh Pocket	RR[3044]	0.49	1.55	4.41	38
Wavell Heights	RR[3045]	0.93	1.35	1.93	7606
Waverley (Qld)	RR[3046]	0.59	1.58	4.55	17
Wealwandangie	RR[3047]	0.33	0.99	2.77	25
Webb	RR[3048]	**1.03**	**2.67**	**5.86**	301
Weengallon	RR[3049]	0.30	0.91	2.78	30

Weerriba	RR[3050]	0.25	0.97	3.15	0
Weipa Airport	RR[3051]	0.26	1.06	4.44	0
Weir River	RR[3052]	0.30	0.83	2.64	21
Welcome Creek	RR[3053]	0.27	0.73	1.97	354
Wellcamp	RR[3054]	0.30	0.84	2.22	243
Wellesley (Qld)	RR[3055]	0.30	0.96	2.88	57
Wellesley Islands	RR[3056]	0.30	1.26	3.34	793
Wellington Point	RR[3057]	0.56	0.85	1.26	9925
Welsby	RR[3058]	0.31	1.30	5.09	5
Wengenville	RR[3059]	0.31	0.87	2.40	36
Wenlock	RR[3060]	0.35	1.18	3.64	3
Weranga	RR[3061]	0.34	0.98	2.77	168
Weribone	RR[3062]	0.33	0.97	2.88	9
West End (Brisbane - Qld)	RR[3063]	0.77	1.16	1.63	8256
West End (Townsville - Qld)	RR[3064]	0.66	1.16	1.93	3468
West Gladstone	RR[3065]	0.56	0.99	1.69	3875
West Haldon	RR[3066]	0.24	0.69	1.92	57
West Ipswich	RR[3067]	0.59	1.46	3.52	418
West Mackay	RR[3068]	**1.24**	**1.84**	**2.61**	5097
West Point	RR[3069]	0.74	2.61	8.42	32
West Prairie	RR[3070]	0.26	0.74	1.93	65
West Rockhampton	RR[3071]	0.87	1.69	3.08	1459
West Stowe	RR[3072]	0.25	0.70	1.78	245
West Wellesley Islands	RR[3073]	0.14	1.17	8.04	0
West Woombye	RR[3074]	0.36	0.83	1.83	832
Westbrook (Qld)	RR[3075]	0.28	0.59	1.15	2798
Westcourt	RR[3076]	**1.29**	**2.04**	**3.15**	3382
Western Creek (Qld)	RR[3077]	0.25	0.82	2.31	15
Westgrove	RR[3078]	0.33	1.06	3.13	3
Westlake	RR[3079]	0.22	0.47	0.95	3564
Westmar	RR[3080]	0.28	0.90	2.77	41
Westvale	RR[3081]	0.30	0.82	2.31	0
Westwood (Qld)	RR[3082]	0.54	1.50	3.82	138
Wetheron	RR[3083]	0.33	0.93	2.72	37
Weyba Downs	RR[3084]	0.29	0.88	2.30	246
Wharps	RR[3085]	0.40	1.25	3.65	0
Wheatlands	RR[3086]	0.32	0.87	2.23	76
Wheatvale	RR[3087]	0.28	0.82	2.30	49
Whetstone	RR[3088]	0.36	0.93	2.99	53
Whichello	RR[3089]	0.27	0.80	2.60	25
White Mountain	RR[3090]	0.25	0.74	2.21	18
White Patch	RR[3091]	0.32	1.19	4.19	127
White Rock (Cairns - Qld)	RR[3092]	0.70	1.20	1.87	3684
White Rock (Ipswich - Qld)	RR[3093]	0.24	0.66	1.92	3
Whiteside	RR[3094]	0.24	0.64	1.60	606
Whitfield (Qld)	RR[3095]	0.82	1.36	2.21	3406
Whitsundays	RR[3096]	0.10	0.32	0.84	2107
Whyanbeel	RR[3097]	0.45	1.18	3.18	142
Widgee	RR[3098]	0.46	1.05	2.29	668
Widgee Crossing North	RR[3099]	0.25	0.73	2.09	18

Widgee Crossing South	RR[3100]	0.23	0.71	2.26	35
Widgeegoara	RR[3101]	0.34	1.06	3.09	26
Wieambilla	RR[3102]	0.29	0.88	2.56	70
Wights Mountain	RR[3103]	0.21	0.55	1.48	641
Wigton	RR[3104]	0.34	0.92	2.62	4
Wildash	RR[3105]	0.35	0.99	2.75	54
Wilkesdale	RR[3106]	0.32	0.90	2.34	96
Willawong	RR[3107]	0.27	0.74	1.87	157
Willow Vale (Qld)	RR[3108]	0.13	0.37	0.89	1595
Willowbank	RR[3109]	0.18	0.47	1.20	1063
Willows	RR[3110]	0.52	1.52	3.69	133
Willowvale (Qld)	RR[3111]	0.27	0.77	2.04	78
Wills	RR[3112]	0.47	1.49	4.69	14
Wilson Beach	RR[3113]	0.34	1.13	3.71	49
Wilson Valley	RR[3114]	0.29	0.94	3.10	14
Wilsons Plains	RR[3115]	0.22	0.63	1.83	44
Wilsons Pocket	RR[3116]	0.34	1.04	2.96	163
Wilsonton	RR[3117]	0.81	1.23	1.93	4798
Wilsonton Heights	RR[3118]	0.34	0.72	1.41	2091
Wilston	RR[3119]	0.85	1.40	2.27	3124
Winchester	RR[3120]	0.29	0.84	2.54	5
Windaroo	RR[3121]	0.30	0.64	1.32	2236
Windera (Qld)	RR[3122]	0.28	0.81	2.37	70
Windermere (Qld)	RR[3123]	0.36	0.91	2.47	139
Windeyer (Qld)	RR[3124]	0.39	1.15	3.22	22
Windorah	RR[3125]	0.39	1.22	3.35	94
Windsor (Qld)	RR[3126]	1.00	1.49	2.15	5958
Winfield	RR[3127]	0.45	1.30	3.37	137
Winston	RR[3128]	0.64	1.39	2.83	908
Winton (Qld)	RR[3129]	**2.11**	**4.12**	**6.95**	727
Winwill	RR[3130]	0.22	0.63	1.92	129
Winya	RR[3131]	0.27	0.77	2.31	58
Wishart (Qld)	RR[3132]	0.24	0.42	0.69	8563
Withcott	RR[3133]	0.19	0.46	1.00	1447
Witheren	RR[3134]	0.29	0.75	2.02	422
Witta	RR[3135]	0.27	0.62	1.37	1036
Wivenhoe Hill	RR[3136]	0.27	0.82	2.70	4
Wivenhoe Pocket	RR[3137]	0.33	0.85	2.18	355
Wiyarra	RR[3138]	0.22	0.76	2.13	16
Woleebee	RR[3139]	0.35	0.88	2.60	95
Wolfang	RR[3140]	0.28	0.88	2.43	59
Wolffdene	RR[3141]	0.21	0.59	1.46	237
Wolvi	RR[3142]	0.25	0.67	1.72	375
Womalilla	RR[3143]	0.36	1.02	3.15	36
Womblebank	RR[3144]	0.38	1.09	3.10	10
Womina	RR[3145]	0.25	0.72	1.96	185
Wonbah	RR[3146]	0.26	0.79	2.38	87
Wonbah Forest	RR[3147]	0.27	0.85	2.64	5
Wondai	RR[3148]	0.90	1.72	2.86	1626
Wondalli	RR[3149]	0.31	0.96	2.67	52

Wondecla	RR[3150]	0.31	0.83	2.03	534
Wondunna	RR[3151]	0.40	0.82	1.52	2042
Wonga Beach	RR[3152]	0.69	1.59	3.27	766
Wongabel	RR[3153]	0.36	0.99	2.77	182
Wongaling Beach	RR[3154]	0.44	1.12	2.45	1035
Wongawallan	RR[3155]	0.42	0.95	2.07	989
Wonglepong	RR[3156]	0.31	0.82	2.09	286
Woocoo	RR[3157]	0.31	0.87	2.56	44
Woodbine (Qld)	RR[3158]	0.24	0.69	2.00	20
Woodbury (Qld)	RR[3159]	0.28	0.76	1.94	358
Woodend (Qld)	RR[3160]	0.53	1.18	2.43	1060
Wooderson	RR[3161]	0.26	0.74	2.17	161
Woodford (Qld)	RR[3162]	0.67	1.17	1.96	3033
Woodgate	RR[3163]	0.66	1.39	2.78	1074
Woodhill (Qld)	RR[3164]	0.20	0.54	1.41	523
Woodlands (Qld)	RR[3165]	0.23	0.67	1.99	89
Woodleigh (Qld)	RR[3166]	0.23	0.85	2.83	3
Woodmillar	RR[3167]	0.29	0.89	2.65	83
Woodridge (Qld)	RR[3168]	0.64	0.94	1.34	9565
Woodstock (Qld)	RR[3169]	0.70	1.88	4.56	194
Woodwark	RR[3170]	0.25	0.73	2.04	295
Woody Point	RR[3171]	0.80	1.32	2.09	3793
Woolein	RR[3172]	0.29	0.91	3.03	7
Woolgar	RR[3173]	0.49	1.31	4.14	5
Woolloongabba	RR[3174]	0.64	1.05	1.67	5079
Woolmar	RR[3175]	0.26	0.69	1.70	334
Woolmer	RR[3176]	0.22	0.68	1.94	31
Woolooga	RR[3177]	0.29	0.79	2.16	200
Woolooman	RR[3178]	0.20	0.66	2.20	20
Wooloowin	RR[3179]	**1.01**	**1.65**	**2.58**	3340
Woolshed	RR[3180]	0.25	0.72	2.08	5
Woombye	RR[3181]	0.53	1.00	1.82	2541
Woondum	RR[3182]	0.22	0.68	2.08	63
Woongarra	RR[3183]	0.63	1.45	3.04	460
Woongoolba	RR[3184]	0.50	1.33	3.28	224
Woopen Creek	RR[3185]	0.38	1.05	2.89	125
Woorabinda	RR[3186]	0.40	1.02	2.51	638
Woorim	RR[3187]	**1.05**	**1.98**	**3.41**	1631
Wooroolin	RR[3188]	0.34	0.94	2.36	262
Wooroona	RR[3189]	0.34	0.95	2.90	7
Wooroonden	RR[3190]	0.28	0.82	2.44	45
Wooroonooran	RR[3191]	0.43	1.18	3.13	0
Wootha	RR[3192]	0.29	0.86	2.62	168
Woowoonga	RR[3193]	0.31	0.88	2.72	84
Woree	RR[3194]	0.57	1.02	1.65	3850
Worongary	RR[3195]	0.49	0.86	1.39	4493
Wowan	RR[3196]	0.31	0.90	2.34	184
Wrattens Forest	RR[3197]	0.30	0.83	2.37	3
Wrights Creek (Qld)	RR[3198]	0.37	1.04	3.01	132
Wrotham	RR[3199]	0.44	1.30	3.73	0

Wujal Wujal	RR[3200]	**1.06**	**3.09**	**7.79**	220
Wulguru	RR[3201]	0.41	0.76	1.34	3772
Wulkuraka	RR[3202]	0.36	0.87	1.94	880
Wunjunga	RR[3203]	0.35	1.24	3.65	9
Wura	RR[3204]	0.36	1.05	3.34	8
Wurdong Heights	RR[3205]	0.22	0.58	1.55	370
Wurtulla	RR[3206]	0.52	0.93	1.47	4737
Wuruma Dam	RR[3207]	0.31	0.82	2.43	32
Wutul	RR[3208]	0.27	0.83	2.48	37
Wyaga	RR[3209]	0.31	0.83	2.68	71
Wyalla	RR[3210]	0.31	0.86	2.45	31
Wyandra	RR[3211]	0.37	1.06	2.93	88
Wyaralong	RR[3212]	0.24	0.67	1.96	13
Wyberba	RR[3213]	0.27	0.96	3.04	67
Wycarbah	RR[3214]	0.44	1.39	3.60	39
Wychie	RR[3215]	0.30	0.83	2.45	14
Wycombe	RR[3216]	0.32	0.98	2.86	32
Wynnum	RR[3217]	**1.19**	**1.59**	**2.08**	10583
Wynnum West	RR[3218]	**1.01**	**1.44**	**1.88**	9939
Wyreema	RR[3219]	0.33	0.75	1.67	1337
Wyuna (Qld)	RR[3220]	0.28	0.83	2.42	35
Yaamba	RR[3221]	0.34	0.96	2.73	54
Yabulu	RR[3222]	0.32	0.81	1.88	592
Yagaburne	RR[3223]	0.28	0.85	3.00	13
Yagoonya	RR[3224]	0.46	1.40	4.14	20
Yalangur	RR[3225]	0.26	0.75	1.92	57
Yalboroo	RR[3226]	0.32	0.93	2.65	124
Yamanto	RR[3227]	0.20	0.43	0.85	3704
Yandaran	RR[3228]	0.43	1.10	2.67	370
Yandarlo	RR[3229]	0.38	1.10	3.13	14
Yandilla	RR[3230]	0.26	0.77	2.56	43
Yandina	RR[3231]	0.55	1.09	2.02	1933
Yandina Creek	RR[3232]	0.24	0.57	1.39	581
Yangan	RR[3233]	0.30	0.79	1.90	318
Yargullen	RR[3234]	0.25	0.75	2.18	22
Yaroomba	RR[3235]	0.20	0.50	1.21	1241
Yarrabah	RR[3236]	**1.81**	**3.06**	**4.90**	1692
Yarrabilba	RR[3237]	0.12	0.32	0.72	2455
Yarraden	RR[3238]	0.42	1.30	3.93	11
Yarraman (Qld)	RR[3239]	0.43	1.00	2.14	902
Yarranlea	RR[3240]	0.26	0.74	2.15	73
Yarrol	RR[3241]	0.30	0.85	2.25	5
Yarwun	RR[3242]	0.36	1.05	2.96	97
Yatala	RR[3243]	0.23	0.55	1.21	1072
Yeerongpilly	RR[3244]	0.45	1.01	1.99	1617
Yelarbon	RR[3245]	0.78	2.03	4.90	283
Yenda (Qld)	RR[3246]	0.32	0.88	2.63	20
Yengarie	RR[3247]	0.24	0.70	1.76	364
Yeppoon	RR[3248]	**1.84**	**2.55**	**3.40**	5367
Yeronga	RR[3249]	0.99	1.52	2.23	5629

Yerra	RR[3250]	0.31	0.83	2.20	89
Yimbun	RR[3251]	0.30	0.81	2.51	30
Yorkeys Knob	RR[3252]	0.95	1.69	2.74	2365
Yowah	RR[3253]	0.37	1.11	3.03	129
Yugar	RR[3254]	0.20	0.59	1.71	290
Yulabilla	RR[3255]	0.32	0.93	2.71	26
Yuleba	RR[3256]	0.27	0.85	2.37	164
Yuleba North	RR[3257]	0.31	0.91	2.44	58
Yuleba South	RR[3258]	0.30	0.93	2.60	27
Yungaburra	RR[3259]	0.30	0.79	1.77	990
Yuruga	RR[3260]	0.38	1.09	3.10	61
Zillmere	RR[3261]	0.89	1.33	1.84	7402
Zilzie	RR[3262]	0.49	1.02	1.97	2041
